# Peripheral Templation Generates an M^II^_6_L_4_ Guest‐Binding Capsule

**DOI:** 10.1002/anie.201602135

**Published:** 2016-04-20

**Authors:** Felix J. Rizzuto, Wen‐Yuan Wu, Tanya K. Ronson, Jonathan R. Nitschke

**Affiliations:** ^1^ Department of ChemistryUniversity of CambridgeLensfield RoadCambridgeCB2 1EWUK; ^2^ College of Chemistry and Molecular EngineeringNanjing Tech UniversityNanjing211800P.R. China).

**Keywords:** host–guest chemistry, platonic solids, self-assembly, supramolecular chemistry, templation

## Abstract

Pseudo‐octahedral M^II^_6_L_4_ capsules result from the subcomponent self‐assembly of 2‐formylphenanthroline, threefold‐symmetric triamines, and octahedral metal ions. Whereas neutral tetrahedral guests and most of the anions investigated were observed to bind within the central cavity, tetraphenylborate anions bound on the outside, with one phenyl ring pointing into the cavity. This binding configuration is promoted by the complementary arrangement of the phenyl rings of the intercalated guest between the phenanthroline units of the host. The peripherally bound, rapidly exchanging tetraphenylborate anions were found to template an otherwise inaccessible capsular structure in a manner usually associated with slow‐exchanging, centrally bound agents. Once formed, this cage was able to bind guests in its central cavity.

Abiological molecules that can recognize, process, and transport chemical species provide insight into the fundamentals of complex natural chemical systems.^1^ Just as enzymes bind substrates in a pocket, capsular supramolecular structures can bind guests: the possession of an internal void allows for the uptake and storage of molecular payloads,^2^ which can subsequently be released or transformed upon the receipt of appropriate signals.^3^ Modifying the size and shape of a capsule enables the targeted sequestration of specific molecules, with form creating function at the molecular level.

Although most often designed to encapsulate guests centrally, a few cages have the ability to bind molecules in more than one mode or location.^4^ Several strategies—closing off faces,^5^ using hydrophobic substituents,^6^ and manipulating ligand configuration^7^ and electronics^8^—have been used to promote the encapsulation of specific guests within an internal cavity; however, design principles to generate architectures that can bind guests peripherally remain largely elusive. The solvophobicity of guests, the size complementarity between a guest and the cavity of a host, and the cooperative enthalpic interactions that result from having multiple ligands enclosing a central species tend to favor internal encapsulation, thereby making it the predominant mode of binding observed in supramolecular systems.^9^

Templation, likewise, typically involves a series of subunits assembling around a central structure‐defining feature.^10^ When this feature remains bound within the product structure, no cavity is available for storing other molecules; only when the template is displaced can the structure bind a different guest.^11^

Herein, we present the syntheses and host–guest chemistry of a new class of supramolecular pseudo‐octahedra capable of binding neutral and anionic guests, in both internal and peripheral pockets. These new M^II^_6_L_4_ architectures were generated by employing 2‐formylphenanthroline (a tridentate component) in place of 2‐formylpyridine (a bidentate component) during the subcomponent self‐assembly process. Binding investigations of these hosts with a range of tetrahedral, octahedral, and icosahedral guests revealed that one cage could simultaneously bind two guests in two different locations, and that peripheral guests bound more strongly than internal ones.

In one case, peripheral guests were observed to template the formation of a Cd^II^_6_L_4_ structure, despite the failure of this structure to form either directly by subcomponent self‐assembly or through the use of guest templation with centrally encapsulated guests. Once formed by peripheral templation, this capsule was capable of encapsulating guests centrally. This observation highlights the importance of peripheral templation in the generation of otherwise inaccessible host–guest species.

The reaction of 2‐formylphenanthroline (12 equiv), zinc(II) trifluoromethanesulfonate (triflate, OTf^−^; 6 equiv), and either triamine **A** or **B** in CH_3_CN led to the formation of Zn^II^_6_L_4_ assemblies **1** or **2**, respectively, after heating for 16 h at 70 °C (Scheme 1), as confirmed by ESI‐MS. The ^1^H NMR spectra indicated the formation of highly symmetrical products in solution. DOSY NMR spectroscopy confirmed that the aromatic signals corresponded to a single species in both cases (see Sections 2 and 3 in the Supporting Information for full characterization).

**Scheme 1:**
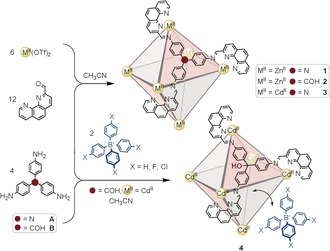
Syntheses of M^II^_6_L_4_ capsules **1**–**3** (top) compared to the synthesis of **4** (bottom), which only formed in the presence of tetraphenylborates. Red faces are occupied by ligands, gray faces are open.

Under all conditions tried, **4** was never observed to assemble from Cd^II^, **B**, and 2‐formylphenanthroline; only in the presence of a peripherally bound tetraphenylborate template was **4** observed. Subcomponent **A**, however, was observed to assemble with Cd^II^ and 2‐formylphenanthroline to yield **3** (see Section 4 in the Supporting Information). ESI‐MS confirmed the 4:6 metal/ligand ratio of the structure, while NMR spectroscopy revealed a highly symmetric species, as with **1** and **2**. HR‐ESI‐MS unambiguously established the stoichiometry in all cases.

Single crystals of **1** and **2** were grown by slow diffusion of Et_2_O or *i*Pr_2_O, respectively, into CH_3_CN solutions.^12^ X‐ray diffraction analyses confirmed that both Zn^II^_6_L_4_ complexes comprise an octahedral framework of metal ions, in which the octahedron faces are alternately occupied by a ligand or an open aperture (Figure 1 a–c). The connectivity of **1** and **2** is thus akin to those of the hexanuclear architectures reported by the groups of Fujita^13^ and Yan.^14^ Both unit cells contain a racemic mixture of the all‐Δ and all‐Λ configurations exclusively. This observation is consistent with their NMR spectra in solution, which indicate that both **1** and **2** contain metal centers of a single handedness, with approximate *T* (chiral tetrahedral) point symmetry.

**Figure 1:**
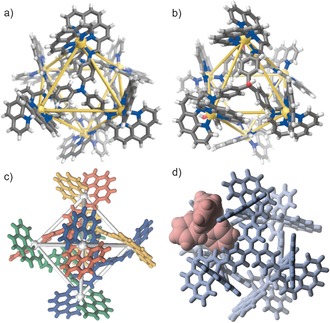
Crystal structures of: a) **1** and b) **2**, viewed down the *C*_3_ axis (Zn: yellow, N: blue, O: red, C: gray, H: white, anions and solvent are omitted for clarity). c) View down the *C*_2_ axis of **1**, showing the complexation of the imino‐phenanthroline moieties about the metal centers (each ligand is rendered in a different color, Zn: gray). d) MM2 molecular model of BPh_4_^−^⋅**1**, showing the *exo* binding mode of BPh_4_^−^.

The crystal structure of **2** presents a more distorted octahedral Zn^II^ coordination environment than in **1**; the angles between the chelate planes of the imino‐phenanthroline units were observed to be 82–90° in **1**, versus 71–90° in **2**. Similarly, whereas **1** displayed uniform diagonal Zn^II^–Zn^II^ distances of about 17 Å, the corresponding diagonals in **2** measured approximately 15×16×19 Å,^15^ thus reflecting a significant axial elongation and equatorial compression in **2**. Although of similar areas (20–30 Å^2^), the open triangular apertures of **2** resultantly present a less equilateral surface than **1**. Void volumes, calculated using VOIDOO,^16^ revealed cavities of 282 Å^3^ for **1** and 423 Å^3^ for **2**. These volumes are approximately ten times larger than those of the M^II^_4_L_4_ tetrahedra formed from the same triamines using 2‐formylpyridine.^17^

We reasoned that, given the tetrahedrally arranged apertures of the hosts, we could employ the voids of **1**–**3** to bind tetrahedral guests. Three classes of tetrahedral prospective guests were investigated: neutral molecules, smaller anions, and larger anions incorporating aromatic units. Whereas the anions BF_4_^−^, ClO_4_^−^, SO_4_^2−^, and PO_4_^3−^ were not observed by ^1^H or ^19^F NMR spectroscopy to bind within **1**–**3** at 25 °C, the addition of polyhalogenated CI_4_, CBr_4_, or CI_3_H led to shifts in the ^1^H NMR spectrum of **2**, consistent with binding in fast exchange on the NMR timescale (Figures S51–S54). As the most pronounced downfield shifts were observed for the phenylene protons of **2**, we infer that binding occurs centrally. No binding was observed for the smaller CHBr_3_, CCl_4_, or less symmetric CH_2_BrI, CHCl_3_, or CH_2_Cl_2_ molecules (Figure 2).

**Figure 2:**
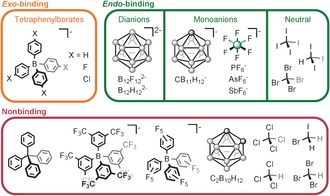
Summary of the host–guest chemistry of capsule **2** (all boxes) and capsule **1** and **3** (*exo*‐bound guests only).

Tetraphenylborate (BPh_4_^−^) was observed to bind in intermediate exchange on the NMR timescale (as indicated by significant broadening of cage resonances, followed by the appearance of sharp signals) with **1** and **3**, whereas this anion was observed by ^1^H NMR spectroscopy to bind in fast exchange with **2** (Figures S25, S68, and S38, respectively). Significant shielding of the phenylene protons of the ligand was observed in all cases, indicative of guest interaction at the face of the structure; however, substantial shifts for signals attributed to the imine and its nearest phenanthroline protons were also observed. In both **1** and **3**, upfield shifts exceeding 2 ppm were observed, consistent with strong electronic perturbation at the windows of the architecture.

MM2 molecular modeling studies revealed two potential binding modes of BPh_4_^−^ within **1**–**3**: internal encapsulation, where the phenyl rings branch outwards through the apertures of the structure (Figure S26), or peripheral binding, where only one phenyl ring enters the cavity and the remaining three sit in pockets formed by the bisphenanthroline corners (Figure 1 d).

Tandem 1D selective ^1^H NOESY and ROESY spectra collected on the BPh_4_^−^ adducts of **1**–**3** (Figures S27, S42, S69, and S70) indicated strong NOE correlations between the *ortho* protons of BPh_4_^−^ and the phenylene, imine, and adjacent phenanthroline protons of **1**–**3**. Irradiation of the *meta* BPh_4_^−^ protons revealed considerably weaker NOE interactions with the phenylene protons of **1**–**3** and the two phenanthroline protons closest to the imine; no NOE signals were observed between the *para* BPh_4_^−^ protons and **1**–**3**. Consideration of the molecular models of BPh_4_^−^⋅**1** revealed that only in the peripherally bound structure were the *ortho* protons of BPh_4_^−^ sufficiently close to the phenanthroline corners to produce the observed NOE correlations (Figures S26 and S27). Although adjacent to the phenylene rings of the ligand, the *ortho* protons of an internally bound BPh_4_^−^ would be too far away for NOE interactions to be observed to the imine or phenanthroline protons. Consequently, a peripheral binding model, in which a phenyl ring of the guest protrudes through a window of the architecture, best describes the binding of BPh_4_^−^ ions to **1**–**3**. This mode is distinct from the external binding described by Raymond and co‐workers, wherein guests undergo nonspecific interactions with the exterior of the cage and are not directly bound in a cavity.^18^

To further probe the binding abilities of **1**–**3**, we treated these hosts with tetra‐*p*‐F‐ and tetra‐*p*‐Cl‐substituted tetraphenylborate anions (B(C_6_H_4_F)_4_^−^ and B(C_6_H_4_Cl)_4_^−^), both of which were observed to bind in fast exchange on the NMR timescale to **1**–**3** (Figures S31–S37, S44–50, and S75–S82). No binding was observed for pentafluoro‐ or bis‐*m*‐CF_3_‐substituted tetraphenylborates, or for the structurally analogous tetraphenylmethane (Figure 2). We infer the increased steric bulk of the polysubstituted tetraphenylborates to prevent the outward‐pointing phenyl rings from resting on the corners of the structure.

A ^1^H–^19^F HOESY spectrum of B(C_6_H_4_F)_4_^−^⋅**1** (Figure S77) furthermore revealed NOE interactions between the *para*‐fluorine substituent of the guest and the protons H5 and H6 of the phenanthroline moiety. These NOE interactions are consistent with the phenyl rings of B(C_6_H_4_F)_4_^−^ resting on the triangular corners of **1**. No such NOE interactions are possible should the guest be internally bound.

Binding constants for the tetraphenylborates (measured by UV/Vis spectroscopy for **1** and **3**, and ^1^H NMR spectroscopy for **2**) were fitted using 1:1 binding isotherms (Table 1). In most cases, we infer that rapid exchange of the tetraphenylborates between sites serves to block the binding of more than one equivalent simultaneously; we infer the high residuals of some fitting profiles to be due to the interaction of more than one anion with the cages at higher guest concentrations (Figure S50). Notably, **1** and **3** bound all peripheral guests more strongly than **2**. The more regular shapes of the apertures of **1** and **3**, compared to those of **2** (Figure 1), may account for this difference in binding strength. No Hammett relationship was observed between the *para* substituents of the tetraphenylborates and their binding strength with **1**–**3**, although BPh_4_^−^ universally bound more strongly than its *para*‐halogenated analogues.

**Table 1:** Summary of the binding constants (*K*_a_, m ^−1^) of monoanionic guests with capsules **1**–**3**.

Guest	**1** ^[a]^	**2** ^[b]^	**3** ^[a]^
*n*Bu_4_NBPh_4_	1.8±0.2×10^6^	1.7±0.1×10^3^	9±2×10^5^
Na[B(C_6_H_4_F)_4_]	3.6±0.2×10^5^	1.2±0.2×10^3^ ^[d]^	2.3±0.2×10^5^
K[B(C_6_H_4_Cl)_4_]	3.3±0.4×10^5^	1.4±0.3×10^3^ ^[d]^	2.4±0.2×10^5^
CsCB_11_H_12_	–^[c]^	1.18±0.02×10^2^	–^[c]^
*n*Bu_4_NPF_6_	–^[c]^	2.99±0.06×10^1^	–^[c]^
KAsF_6_	–^[c]^	2.41±0.03×10^1^	–^[c]^
NaSbF_6_	–^[c]^	1.53±0.09×10^1^	–^[c]^

[a] Measured by UV/Vis spectroscopy. [b] Measured by ^1^H NMR spectroscopy. [c] No binding observed. [d] Higher residuals indicate that a second, weak binding event may be occurring at high concentrations of guest.

Although **2** did not display any significant optical change during titration with any of the tetraphenylborates, UV/Vis titrations of these anions into acetonitrile solutions of **1** and **3** consistently gave a blue‐shift of the π→π* transition of the triphenylamine chromophore as the titration progressed (see Sections 7 and 9 in the Supporting Information). Monitoring the titration of BPh_4_^−^ into **3** by cyclic voltammetry (Figure S74) resulted in a positive shifting of the first and second reduction waves, attributed to the reduction of the imino‐phenanthroline motif. These observations are consistent with a donation of electron density from the guest to the host, and a narrowing of the band gap upon anion binding.

Having established that neutral tetrahedral molecules bind internally and that tetraphenylborates bind peripherally, we investigated the binding abilities of guests of different shapes and sizes. Octahedral hexafluorinated monoanions were observed to bind in fast exchange on the NMR timescale with **2**; downfield shifting of its phenylene protons, along with movement of the imine and adjacent phenanthroline protons, indicated proximity of the anions to the central cavity of the cage (Figures S61–S67). Broadening of the ^19^F signals for AsF_6_^−^ and SbF_6_^−^ was likewise observed, consistent with binding being in fast exchange on the NMR timescale. These anions exhibited only weak association, however, with binding affinities <40 m ^−1^, as determined through ^1^H NMR titrations (Table 1).

A higher affinity for **2** was exhibited by the larger carborate CB_11_H_12_^−^ (*K*_a_=1.18±0.02×10^2^ m ^−1^), which bound in fast exchange with **2** on the NMR timescale (Figure S55). The B_12_H_12_^2−^ and B_12_F_12_^2−^ dianions were also observed to bind within **2** in fast exchange by ^1^H NMR spectroscopy (Figures S58–S60), although their limited solubilities precluded the quantification of their binding affinities. Nevertheless, splitting and downfield shifting of the overlapping signals corresponding to the phenylene protons of **2** upon titration with B_12_F_12_^2−^ and CB_11_H_12_^−^ ions indicated their uptake within the cage cavity. Treatment of **1** or **3** with the same octahedral and icosahedral anions led either to no significant change in their ^1^H NMR spectra or to broadening, which could not be resolved even at −40 °C.

Having thus established that host **2** possesses multiple binding sites that each bind specific anionic guests, we explored the allosteric effects engendered by treating **2** simultaneously with a guest specific to each site (see Section 10 in the Supporting Information). The titration of CsCB_11_H_12_ into a solution of BPh_4_^−^⋅**2** in CD_3_CN did not lead to a significant shift in the ^1^H NMR signals of the bound BPh_4_^−^ (Δ*δ_ortho_*=−0.02 ppm), thus suggesting that concurrent binding of *exo*‐BPh_4_^−^ and *endo*‐CB_11_H_12_^−^ had occurred. Furthermore, the presence of peripherally bound BPh_4_^−^ had no significant effect on the binding affinity of CB_11_H_12_^−^ to the inside of **2** (*K*_a_=1.59±0.08×10^2^ m ^−1^), nor did the presence of *endo*‐bound CB_11_H_12_^−^ considerably alter the binding strength of BPh_4_^−^ to the periphery of the cage (*K*_a_=2.8±0.4×10^3^ m ^−1^). Increasing the concentration of the other guest in both cases did not change the binding constant of either the *endo*‐ or *exo*‐bound guest.

B_12_F_12_^2−^ and BPh_4_^−^ were likewise observed to bind to **2** simultaneously. The addition of K_2_B_12_F_12_ to a solution of BPh_4_^−^⋅**2** led to ^1^H and ^19^F NMR shifts consistent with the encapsulation of B_12_F_12_^2−^. Reversing the order of titration by adding *n*Bu_4_NBPh_4_ to B_12_F_12_^2−^⊂**2** did not significantly alter the ^19^F NMR chemical shift of encapsulated B_12_F_12_^2−^, thereby indicating that B_12_F_12_^2−^ was not ejected from the capsule upon the peripheral binding of BPh_4_^−^. The system thus appears to bind internal and peripheral guests concurrently, with neither allosteric inhibition nor enhancement of binding affinity.

Similar conditions to those used for the syntheses of **1**–**3** proved ineffective for the synthesis of the marginally larger structure **4**. Heating 2‐formylphenanthroline (12 equiv), Cd(OTf)_2_ (6 equiv), and **B** (4 equiv) in CH_3_CN to 60 °C overnight resulted in a broad, ill‐defined aromatic region in the ^1^H NMR spectrum; only free **B** could be positively identified. Having elucidated the unique binding abilities of **2**, we sought to use this knowledge to generate **4** through guest templation. Although the addition of internally binding guests resulted in no significant change in the broad ^1^H NMR spectrum of the precursors of **4**, the addition of *n*Bu_4_NBPh_4_ (2 equiv) led to the development of signals corresponding to the host–guest species BPh_4_^−^⋅**4** during 12 h of heating at 50 °C (Figure 1). BPh_4_^−^⋅**4** could also be synthesized in a single pot from its subcomponents with BPh_4_^−^ (see Section 11 in the Supporting Information). The diffusion coefficient of capsule **4** was measured to be 9.2×10^−8^ m^2^ s^−1^ by DOSY NMR spectroscopy (Figure S102), similar to the values found for **1** and **2**; BPh_4_^−^ bound to **4** was observed to diffuse more slowly than free BPh_4_^−^. HR‐ESI‐MS confirmed the expected M^II^_6_L_4_ stoichiometry of the resulting species (Figure S101). Notably, no MS signals corresponding to the free cage (without BPh_4_^−^) could be identified, which reflects the strong binding of this anion to **4**.

The use of *para*‐substituted tetraphenylborate templates likewise generated **4**, although two or more equivalents of template were consistently necessary for the formation of the host in all cases (Figures S104–S106). Templation did not, however, occur in the presence of any centrally binding guest; the addition of CB_11_H_12_^−^ or B_12_F_12_^2−^ to its precursors did not produce **4**.

NOE measurements on BPh_4_^−^⋅**4** revealed the same mode of peripheral binding as was observed in the cases of **1**–**3** (Figure S103). The ability of BPh_4_^−^ to template the formation of **4** is thus highly unusual—a template typically sits at the center of the chemical structure that it brings into being, forming a symmetrical host–guest adduct. Furthermore, the high symmetry reflected in the ^1^H NMR spectra of the adducts of **4** with tetraphenylborate and its halogenated congeners indicates that the guest remains in fast exchange on the NMR timescale between different sites on the host at 25 °C. We are not aware of another example of a rapidly exchanging, *exo*‐bound agent templating the formation of a metal–organic capsule.

Although carborate did not serve as a competent template for the formation of **4**, once formed, **4** was observed to bind CB_11_H_12_^−^. The titration of CsCB_11_H_12_ into a solution of BPh_4_^−^⋅**4** in CD_3_CN resulted in ^1^H NMR shifts consistent with the encapsulation of the carborane anion, with a simultaneous shift in the protons of BPh_4_^−^ (Δ*δ_ortho_*=+0.15 ppm; Figure S108). The binding affinity of CB_11_H_12_^−^ for BPh_4_^−^⋅**4** was calculated to be 1.37±0.03×10^2^ m ^−1^, similar to its Zn^II^ analogue **2**.

Employing 2‐formylphenanthroline in place of 2‐formylpyridine during subcomponent self‐assembly thus led to the formation of larger structures with significantly expanded cavities. Understanding the host–guest chemistry of these structures enabled the development of a new mode of templation. The addition of a rapidly exchanging, peripherally bound guest engendered the formation of **4**, which could not be achieved in the template's absence. Moreover, the inclusion complex BPh_4_^−^⋅**4** could still participate in host–guest chemistry. This new mode of templation, which is allosteric in nature, enables the exploration of more complex chemical systems that are capable of responding to multiple stimuli.

## Supporting information

As a service to our authors and readers, this journal provides supporting information supplied by the authors. Such materials are peer reviewed and may be re‐organized for online delivery, but are not copy‐edited or typeset. Technical support issues arising from supporting information (other than missing files) should be addressed to the authors.

Supplementary
