# Supplementary material for: Peripheral Templation Generates an MII 6L4 Guest‐Binding Capsule
Source: Angew Chem Int Ed Engl. 2016 Apr 20;55(28):7958–62. doi: 10.1002/anie.201602135 (PMC4999047; doi:10.1002/anie.201602135)
Supplement: Supplementary file 1 — Supplementary [file ANIE-55-7958-s001.pdf]

Angewandte Chemie
Eine Zeitschrift der Gesellschaft Deutscher Chemiker

Supporting Information

Peripheral Templation Generates an $M^{II}_6L_4$ Guest-Binding Capsule

*Felix J. Rizzuto, Wen-Yuan Wu, Tanya K. Ronson, and Jonathan R. Nitschke**

anie_201602135_sm_miscellaneous_information.pdf

Table of Contents

1. Materials and methods 3
  1.1 Nuclear Magnetic Resonance (NMR) 3
  1.2 Mass spectrometry (MS) 3
  1.3 UV-Vis spectroscopy 4
  1.4 Titrations and Job Plots 4
  1.5 Cyclic voltammetry 5
2. Preparation and characterisation of **1** 6
3. Preparation and characterisation of **2** 10
4. Preparation and characterisation of **3** 14
5. Crystallography 18
  5.1 Crystal structure of **$1·12BF_4·5.25MeCN·0.5Et_2O$** 19
  5.2 Crystal structre of **$2·12(CF_3SO_3)·0.5MeCN$** 20
6. VOIDOO calculations 21
7. Host-guest chemistry of **1** 22
  7.1 $^{n}Bu_4NBPh_4$ 22
  7.2 $Na[B(C_6H_4F)_4]$ 25
  7.3 $K[B(C_6H_4Cl)_4]$ 27
8. Host-guest chemistry of **2** 29
  8.1 $^{n}Bu_4NBPh_4$ 29
  8.2 $Na[B(C_6H_4F)_4]$ 32
  8.3 $K[B(C_6H_4Cl)_4]$ 34
  8.4 CI_4 (tetraiodomethane) 36
  8.5 CBr_4 (tetrabromomethane) 37
  8.6 CHI_3 (iodoform) 37
  8.7 Other halogenated guests 37
  8.8 $CsCB_{11}H_{12}$ 38
  8.9 $K_2B_{12}F_{12}$ 40
  8.10 $Cs_2B_{12}H_{12}$ 41
  8.11 $^{n}Bu_4NPF_6$ 42
  8.12 $KAsF_6$ 43
  8.13 $NaSbF_6$ 45
9. Host-guest chemistry of **3** 46
  9.1 $^{n}Bu_4NBPh_4$ 46
  9.2 $Na[B(C_6H_4F)_4]$ 49

9.3 K[B(C_6H_4Cl)$_4$] 51
10. Dual binding experiments on **2** 53
10.1 $CsCB_{11}H_{12}$ into BPh_4^-•**2** containing 1 equiv. of BPh_4^- 53
10.2 $CsCB_{11}H_{12}$ into BPh_4^-•**2** containing 4 equiv. of BPh_4^- 55
10.3 $^nBu_4NBPh_4$ into $CB_{11}H_{12}^-$⊂**2** containing 1 equiv. of $CB_{11}H_{12}^-$ 56
10.4 $^nBu_4NBPh_4$ into $CB_{11}H_{12}^-$⊂**2** containing 4 equiv. of CB11H12– 57
10.5 $K_2B_{12}F_{12}$ into BPh_4^-•**2** 58
10.6 nBu4NBPh4 into $B_{12}F_{12}^{2-}$⊂**2** 59
11. Synthesis and characterisation of BPh_4^-•**4** 60
12. Host-guest chemistry of BPh_4^-•4 66
12.1 $CsCB_{11}H_{12}$ 66
13. References 67

1. Materials and methods

All reagents were purchased from commercial sources and used as received. For electrochemical experiments, dry solvents were purchased from Sigma-Aldrich and purged with argon before use. $^{n}Bu_4NPF_6$ was recrystallised three times from ethanol before use. 2-Formylphenanthroline[1] and $Cd(OTf)_2$[2] were prepared by literature procedures.

1.1 Nuclear Magnetic Resonance (NMR)

NMR spectra were recorded using a 400 MHz Avance III HD Smart Probe (routine ^{1}H NMR), DCH 500 MHz dual cryoprobe (high-resolution ^{13}C and 2D experiments), DPX S5 500 MHz BB ATM (variable temperature NMR) and 500 MHz TCI-ATM cryo (1D selective NOESY and ROESY experiments, performed by Derrick Roberts at the University of Cambridge) NMR spectrometers. Chemical shifts for ^{1}H, ^{13}C and ^{19}F are reported in ppm on the δ scale; ^{1}H and ^{13}C were referenced to the residual solvent peak and ^{19}F was referenced to an internal standard of C_6F_6 in CD_3CN at −164.9 ppm. Coupling constants (J) are reported in hertz (Hz). The following abbreviations are used to describe signal multiplicity for ^{1}H, ^{13}C and ^{19}F NMR spectra: s: singlet, d: doublet, t: triplet, dd: doublet of doublets; dt: doublet of triplets; m: multiplet, b: broad.

DOSY NMR experiments were performed on a Bruker 500 MHz TCI-ATM cryo NMR spectrometer by Anna McConnell at the University of Cambridge. Maximum gradient strength was 6.57 G/cm A. The standard Bruker pulse program, ledbpgp2s, employing a stimulated echo and longitudinal eddy-current delay (LED) using bipolar gradient pulses for diffusion using 2 spoil gradients was utilised. Rectangular gradients were used with a total duration of 1.5 ms. Gradient recovery delays were 875-1400 µs. Individual rows of the S4 quasi-2D diffusion databases were phased and baseline corrected.

Through-space ^{1}H-^{1}H NMR and variable temperature NMR experiments were performed on cages with 2-4 equivalents of guest.

1.2 Mass spectrometry (MS)

Low resolution electrospray ionisation (LR-ESI) mass spectrometry was undertaken on a Micromass Quattro LC mass spectrometer (cone voltage 10-30 eV; desolvation temp. 313 K;

ionization temp. 313 K) infused from a Harvard syringe pump at a rate of 10 μL min^{-1}. High resolution electrospray ionisation mass spectrometry (HRMS-ESI) was performed on a Waters LCT Premier Mass Spectrometer featuring a Z spray source with electrospray ionisation and modular LockSpray interface.

1.3 UV-Vis spectroscopy

UV-Visible absorption spectroscopy was performed using a Perkin Elmer Lambda 750 UV-Vis-NIR spectrophotometer fitted with a PTP-1 Peltier temperature controller accessory. Spectra were obtained in double beam mode using only the (front) analyte beam to record spectra, with air in the (rear) reference path. A background spectrum of the neat solvent was recorded using the analyte beam prior to each experiment and baseline correction applied using the Perkin Elmer WinLab software suite. Samples were analysed using quartz cuvettes with optical path lengths of 10 mm.

1.4 Titrations and Job Plots

Procedure for UV-Vis titrations: A solution of host ($2.0 – 3.2 \times 10^{-6}$ M in CH_3CN) in a UV-Vis cuvette was titrated with a solution of the same concentration of host and excess guest such that the concentration of the host remained constant with each addition of guest. Upon each addition, the solution was manually stirred for 1 min before acquiring the UV-Vis spectrum.

Procedure for NMR titrations: A 0.6 mL solution of host ($1.2 – 1.4 \times 10^{-3}$ M) in CD_3CN was titrated with a concentrated solution of guest. The total change in concentration of the host was 5.3 – 9.6 % over the course of the titration, and the error involved was assumed to be negligible. Upon each addition, the solution was manually stirred for 1 min before acquiring the spectrum, which allowed equilibrium to be reached between the host and guest.

Procedure for Job Plots:[3] A series of solutions containing **1, 2** or **3** and guest were prepared such that the sum of the total guest and host concentration remained constant ($1.6–2.3 \times 10^{-6}$ M). The mole fraction of the guest was varied from 0.1 to 1.0. The corrected absorbance (mole fraction * absorbance) at 387 nm was plotted against the molar fraction of the guest solution.

1.5 Cyclic voltammetry

Solution state cyclic voltammetry (CV) was performed using a BioLogic SP-150 potentiostat with ferrocene (Fc) as an internal reference. Measurements were conducted under an Ar atmosphere using a conventional three-electrode cell: a glassy carbon working electrode, a Pt wire auxiliary electrode, and a Ag/Ag^+ quasi-reference electrode. A 0.1 M $^{n}Bu_4NPF_6/CH_3CN$ electrolyte was used, with scan rates in the range 25-1000 mV s^{-1}.

2. Preparation and characterisation of **1**

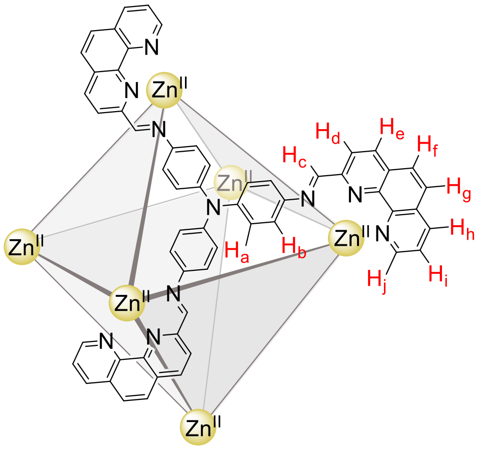

Tris(4-aminophenyl)amine (13.9 mg, 4.80 × 10^{-5} mol, 4 equiv.), 2-formylphenanthroline (30.0 mg, 1.44 × 10^{-5} mol, 12 equiv.) and either $Zn(BF_4)_2 \cdot xH_2O$ (17.2 mg, 7.20 × 10^{-5} mol, 6 equiv. based on anhydrous base) or $Zn(OTf)_2$ (26.2 mg, 7.20 × 10^{-5} mol, 6 equiv.) were combined in CH_3CN and stirred at 70 °C overnight. The solvent was evaporated and the solid triturated with Et_2O to yield **1** as a dark purple crystalline solid ([**1**]$(BF_4)_{12}$: 53.2 mg, 1.08 × 10^{-5} mol, 90%; [**1**]OTf_{12}: 62.5 mg, 1.11 × 10^{-5} mol, 93%).

Note: The ^{1}H and ^{13}C spectral data for **1**$[BF_4]_{12}$ and **1**$[OTf]_{12}$ were identical.

^{1}H NMR (500 MHz, 298 K, CD_3CN): δ 9.50 (s, 12H, H_c), 9.24 (d, J = 8.3 Hz, 12H, H_e), 8.74 (d, J = 8.3 Hz, 12H, H_d), 8.51 (dd, J = 8.1, 1.7 Hz, 12H, H_h), 8.28 (d, J = 9.2 Hz, 12H, H_f), 8.17 (d, J = 9.2 Hz, 12H, H_g), 7.61 – 7.49 (m, 24H, H_i & H_j), 7.11 (d, J = 8.9 Hz, 24H, H_b), 6.59 (d, J = 8.9 Hz, 24H, H_a) ppm.

^{13}C NMR (125 MHz, 298 K, CD_3CN): δ 155.3, 149.1, 147.8, 145.9, 143.5, 140.2, 140.0, 139.9, 139.7, 131.5, 129.9, 129.6, 127.0, 126.6, 126.5, 125.2, 124.1 ppm.

LR-ESI-MS: [charge, calculated for $C_{288}H_{144}N_{40}Zn_6(BF_4)_{12}$]: m/z = 1538.4 [**1**$(BF_4)_9{}^{3+}$, 1539.2], 1132.1 [**1**$(BF_4)_8{}^{4+}$, 1132.7], 888.4 [**1**$(BF_4)_7{}^{5+}$, 888.8], 725.8 [**1**$(BF_4)_6{}^{6+}$, 726.2], 609.8 [**1**$(BF_4)_5{}^{7+}$, 610.0], 522.6 [**1**$(BF_4)_4{}^{8+}$, 522.9].

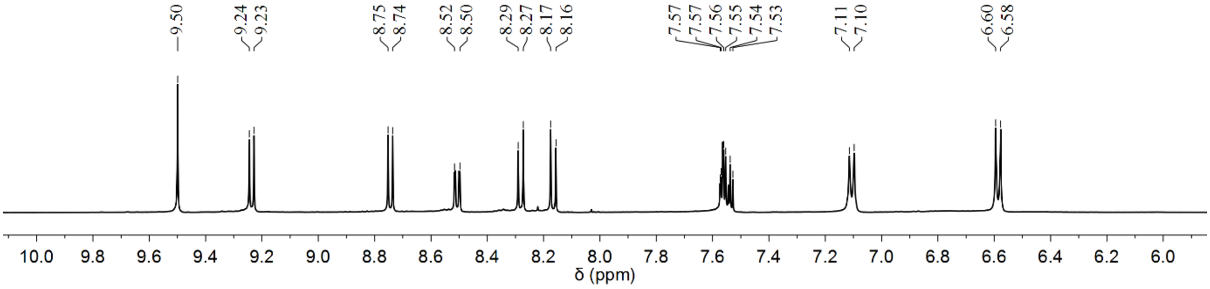

Figure S1. Aromatic region of the ^{1}H NMR spectrum (500 MHz, 298 K, CD_3CN) of **1**$(BF_4)_{12}$.

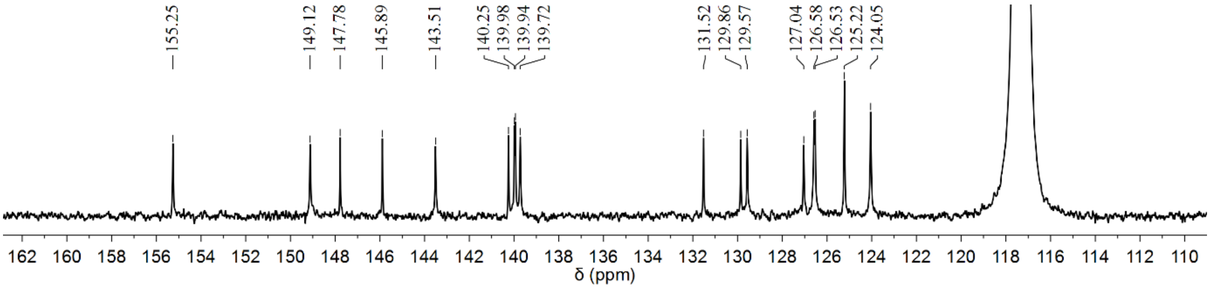

Figure S2. Aromatic region of the ^{13}C NMR spectrum (125 MHz, 298 K, CD_3CN) of **1**$(BF_4)_{12}$.

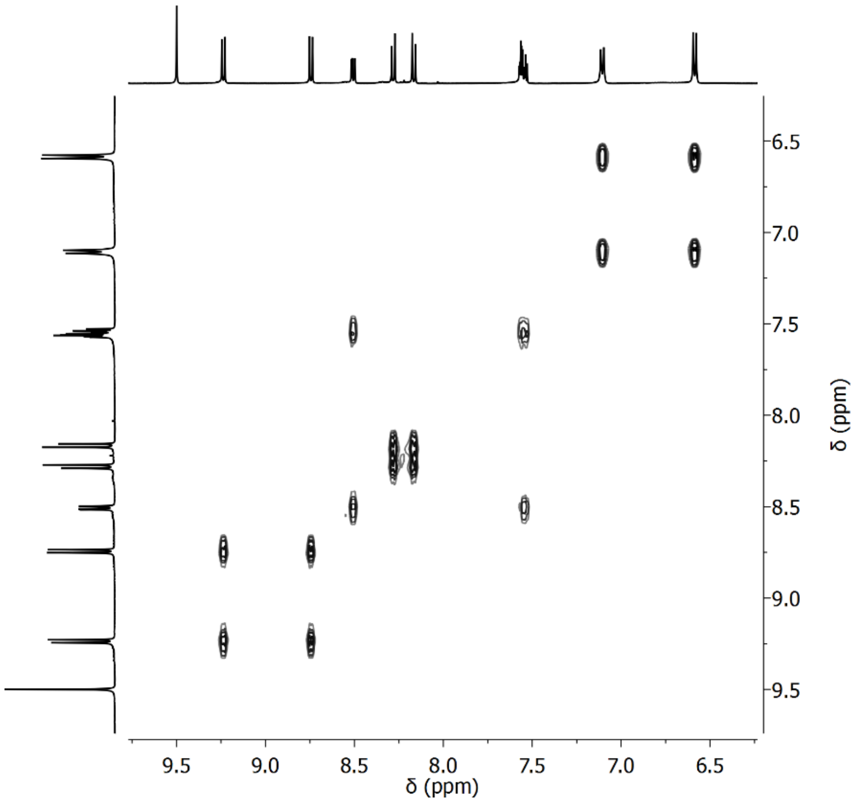

Figure S3. Aromatic region of the ^{1}H-^{1}H COSY spectrum (500 MHz, 298 K, CD_3CN) of **1**$(BF_4)_{12}$.

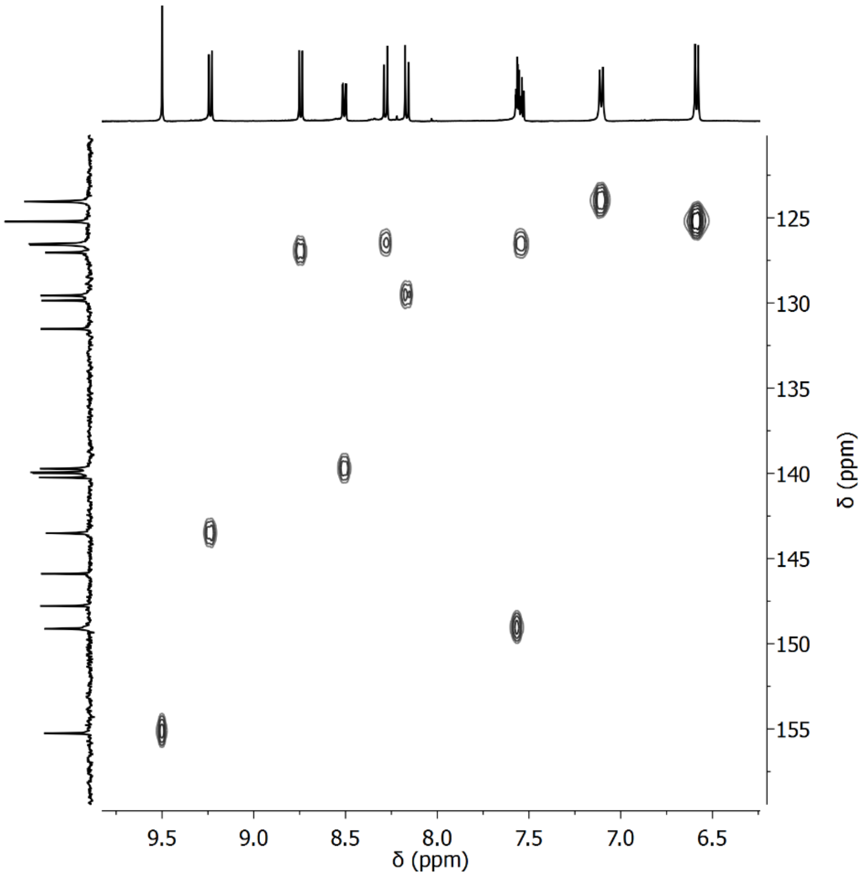

Figure S4. Aromatic region of the ^{1}H-^{13}C HSQC spectrum (500 MHz, 298 K, CD_3CN) of **1**$(BF_4)_{12}$.

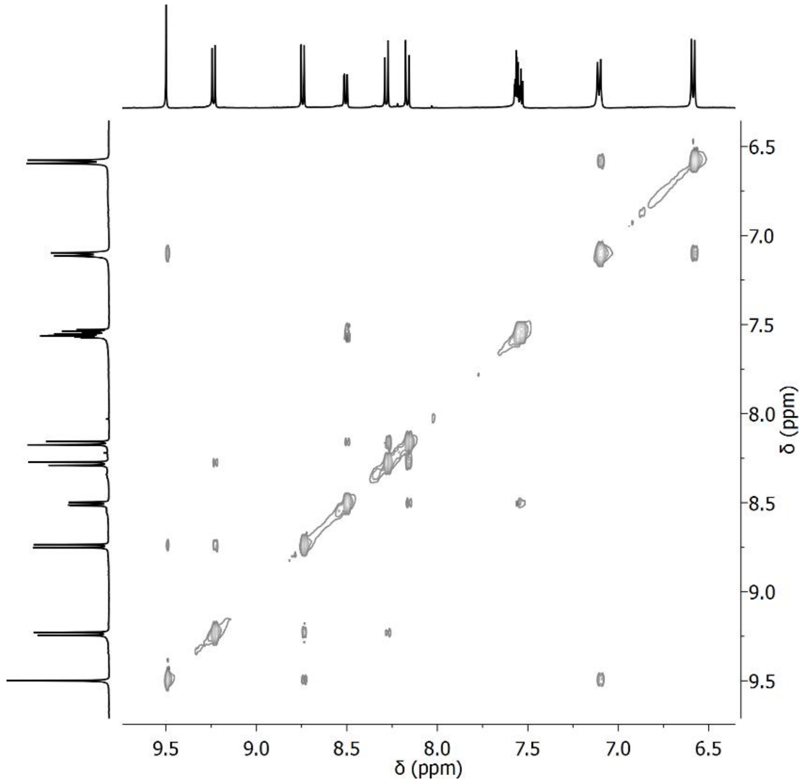

Figure S5. Aromatic region of the ^{1}H-^{1}H NOESY spectrum (500 MHz, 298 K, CD_3CN) of **1**$(BF_4)_{12}$.

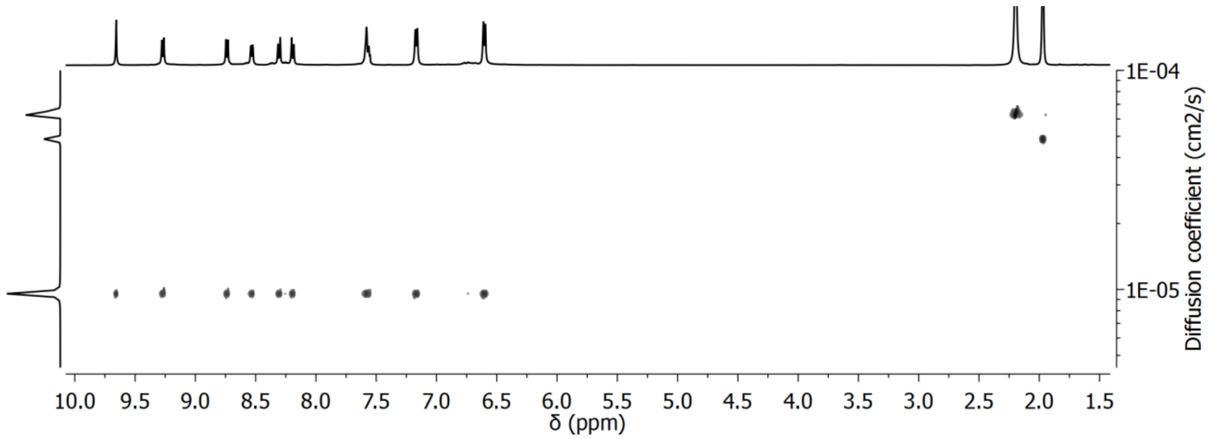

Figure S6. ^{1}H DOSY spectrum (500 MHz, 298 K, CD_3CN) of **1**$(BF_4)_{12}$. The diffusion coefficient was measured to be 9.6×10^{-6} cm^2 s^{-1}.

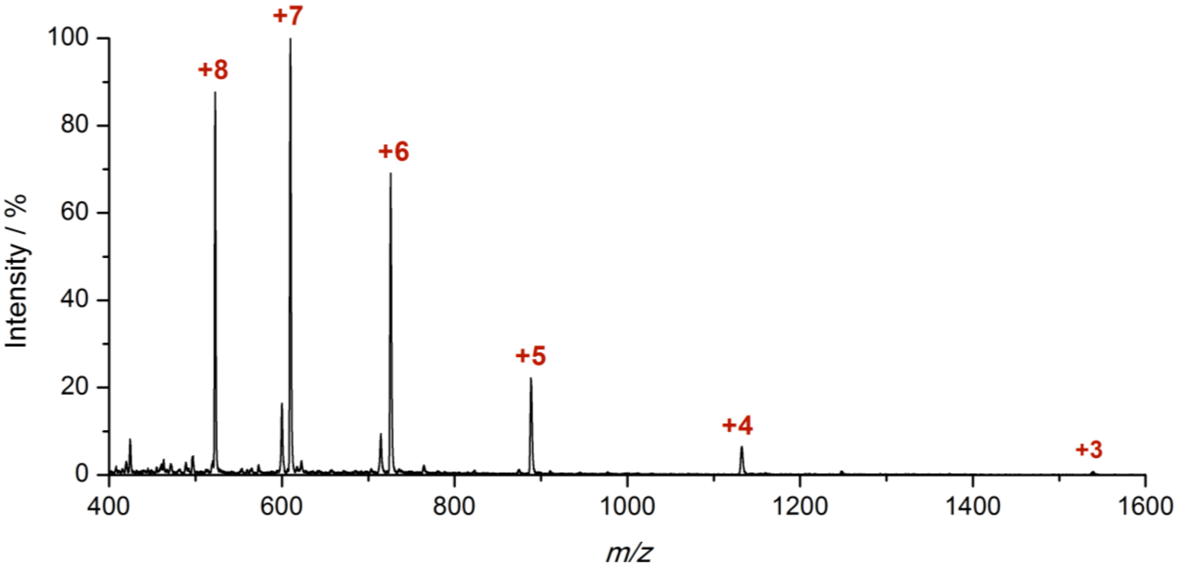

Figure S7. Low resolution ESI mass spectrum of **1**$(BF_4)_{12}$.

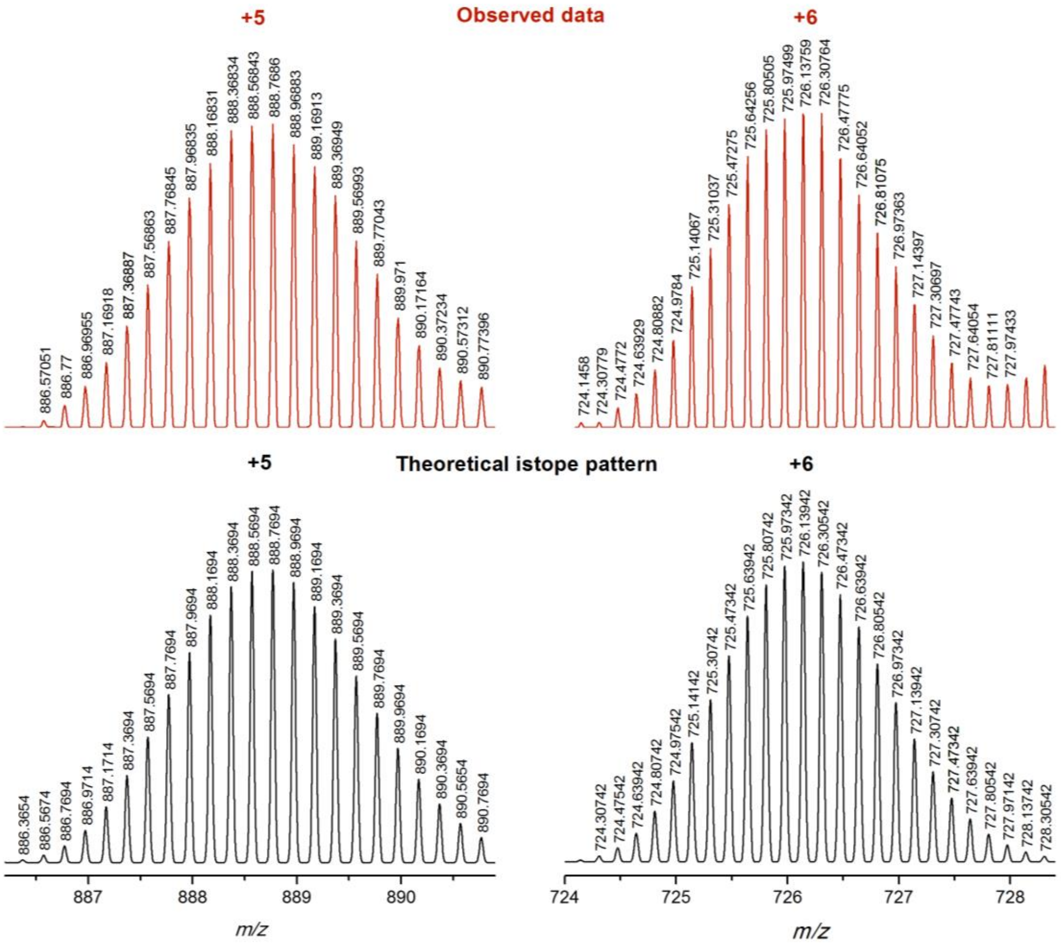

Figure S8. High resolution ESI mass spectra of **1**$(BF_4)_{12}$, showing the observed z = +5 and +6 charges (top), compared to the theoretical isotope patterns for each (bottom).

3. Preparation and characterisation of **2**

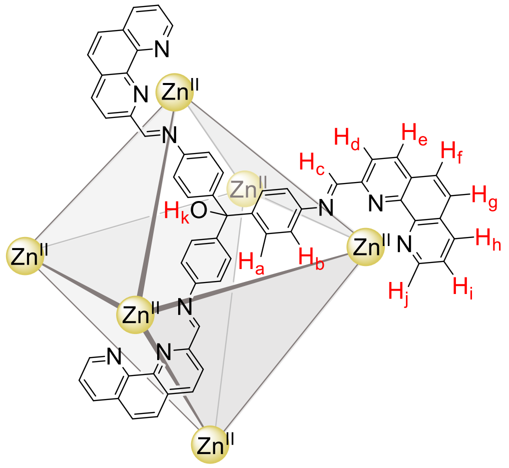

Pararosaniline base (14.7 mg, 4.80×10^{-5} mol, 4 equiv.), 2-formylphenanthroline (30.0 mg, 1.44×10^{-5} mol, 12 eqiv.) and $Zn(OTf)_2$ (26.2 mg, 7.20×10^{-5} mol, 6 equiv.) were combined in CH_3CN and stirred at 70 °C overnight. The complex was precipitated by adding the crude solution to excess Et_2O dropwise. The suspension was centrifuged and filtered to retrieve a purple solid. This was washed with CH_2Cl_2 (2 × 10 mL), EtOH (1 × 10 mL) and $CHCl_3$ (2 × 10 mL) and filtered. The remaining solid was dried *in vacuo* to yield a dark pink solid (55.3 mg, 9.73×10^{-6} mol, 82%).

^{1}H NMR (500 MHz, 298 K, CD_3CN): δ 9.22 (s, 12H, H_c), 9.04 (d, J = 8.3 Hz, 12H, H_e), 8.72 (dd, J = 8.3, 1.5 Hz, 12H, H_h), 8.51 (d, J = 8.3 Hz, 12H, H_d), 8.27 (m, 24H, H_f & H_g), 8.19 (dd, J = 4.8, 1.5 Hz, 12H, H_j), 7.77 (dd, J = 8.3, 4.8 Hz, 12H, H_i), 6.55 (m, 48H, H_a & H_b), 3.96 (s, 4H, H_k) ppm.

^{13}C NMR (125 MHz, 298 K, CD_3CN): δ 160.7, 151.0, 147.5, 146.7, 146.0, 143.9, 141.3, 141.0. 132.0, 130.9, 130.5, 129.4, 128.0, 127.7, 127.5, 123.2, 121.0, 120.6 ppm.

LR-ESI-MS [charge, calculated for $C_{232}H_{148}N_{36}O_4Zn_6(CF_3SO_3)_{12}$]: m/z = 1272.2 [**2**$(OTf)_8^{4+}$, 1272.2], 987.8 [**2**$(OTf)_7^{5+}$, 988.0], 798.3 [**2**$(OTf)_6^{6+}$, 798.4], 663.0 [**2**$(OTf)_5^{7+}$, 663.1], 561.4 [**2**$(OTf)_4^{8+}$, 561.6], 482.5 [**2**$(OTf)_3^{9+}$, 482.6].

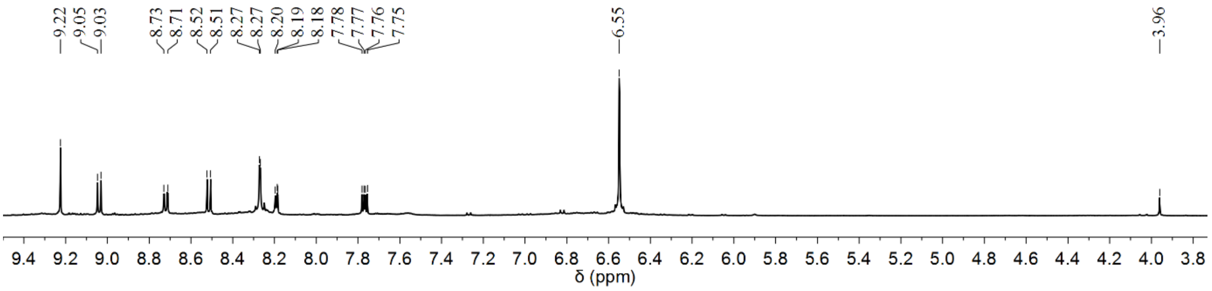

Figure S9. ^{1}H NMR spectrum (500 MHz, 298 K, CD_3CN) of **2**.

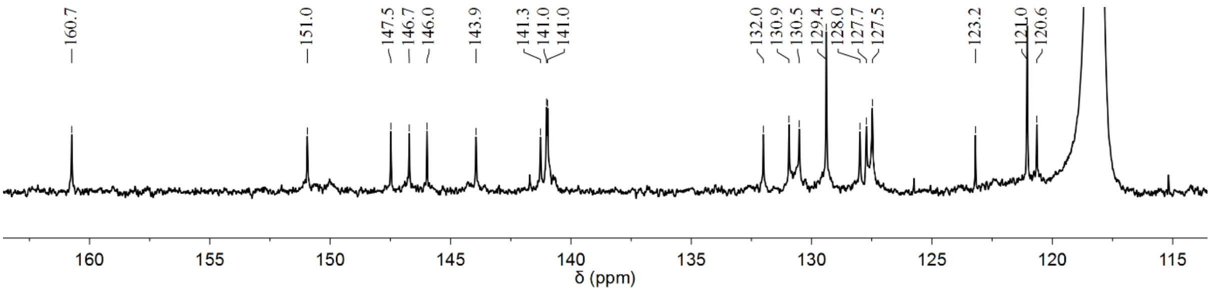

Figure S10. Aromatic region of the ^{13}C NMR spectrum (125 MHz, 298 K, CD_3CN) of **2**.

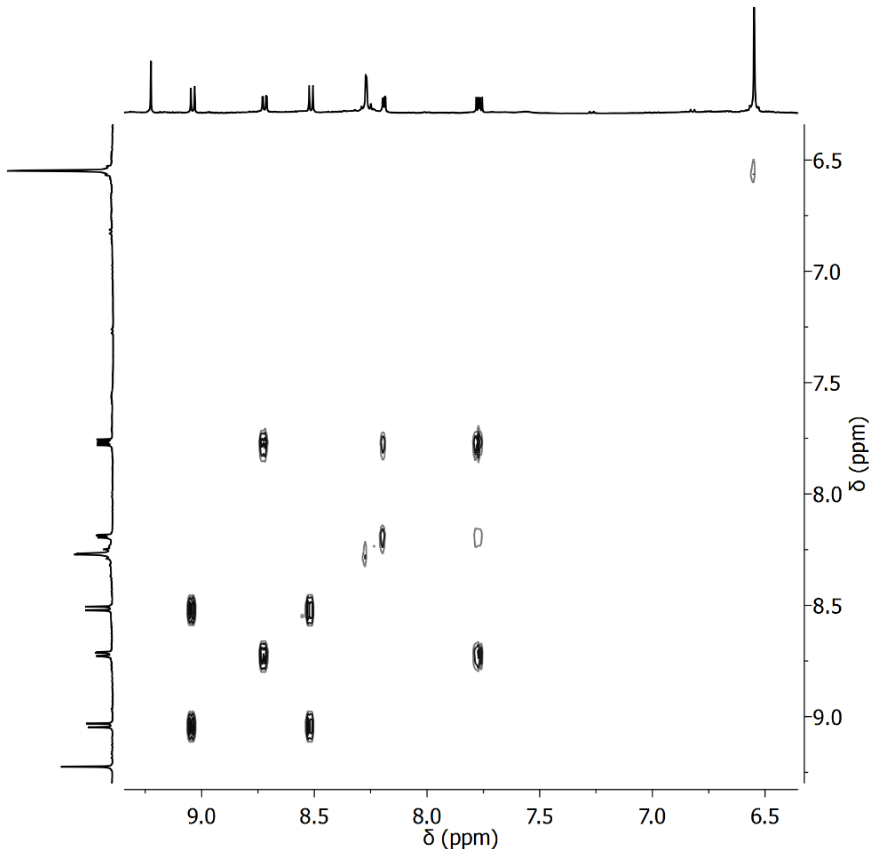

Figure S11. Aromatic region of the ^{1}H-^{1}H COSY spectrum (500 MHz, 298 K, CD_3CN) of **2**.

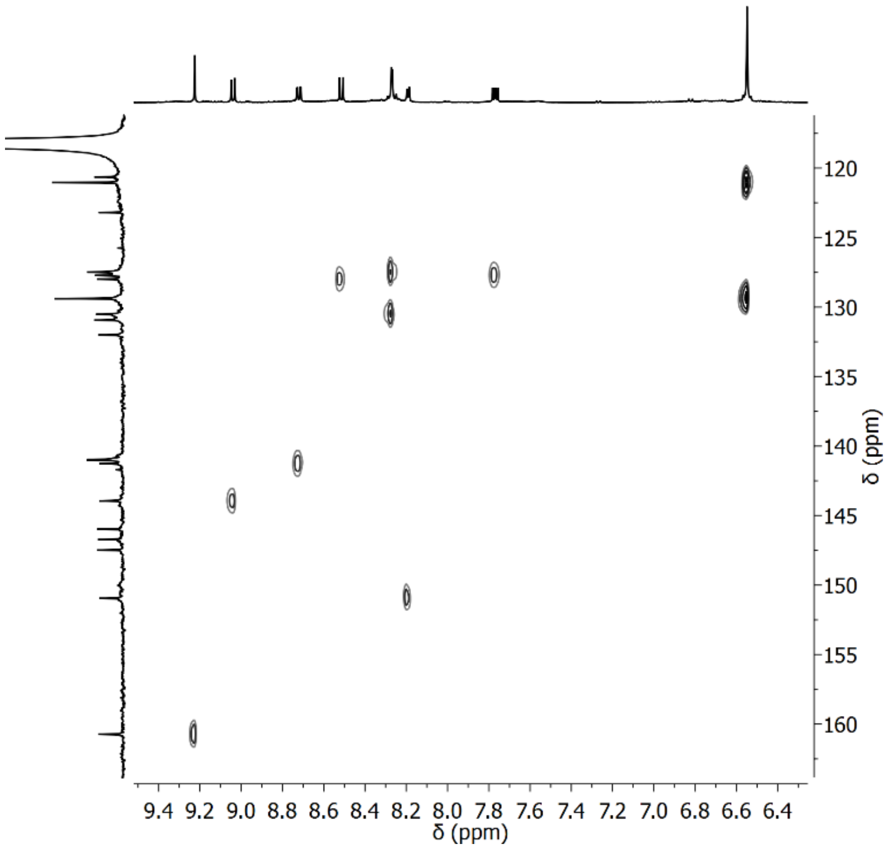

Figure S12. Aromatic region of the ^{1}H-^{13}C HSQC spectrum (500 MHz, 298 K, CD_3CN) of **2**.

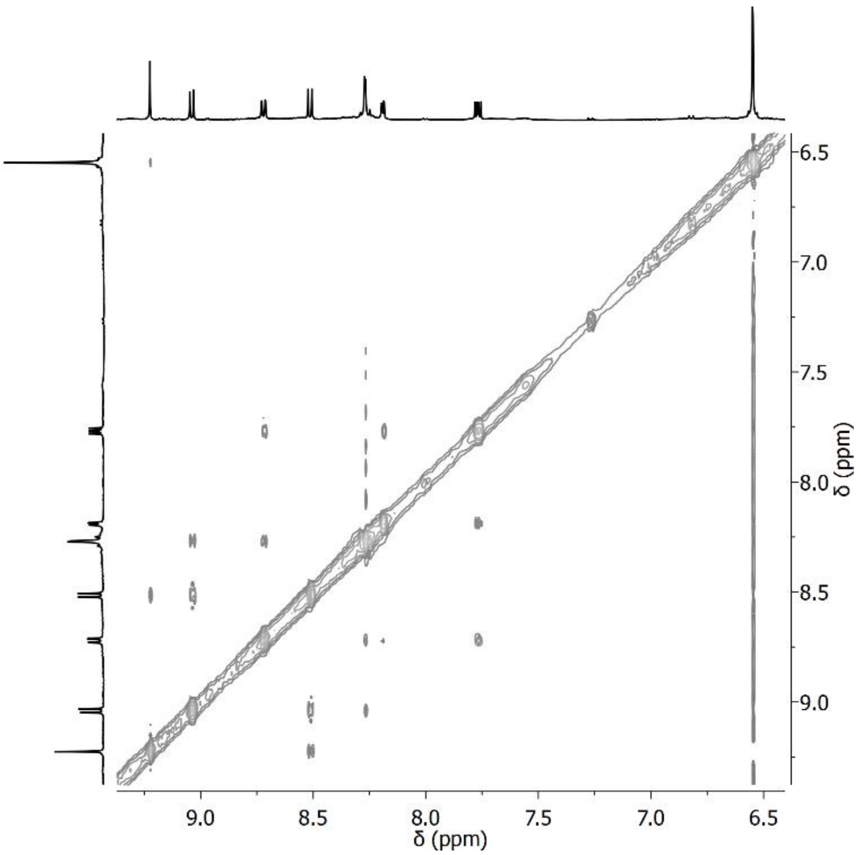

Figure S13. Aromatic region of the ^{1}H-^{1}H NOESY spectrum (500 MHz, 298 K, CD_3CN) of **2**.

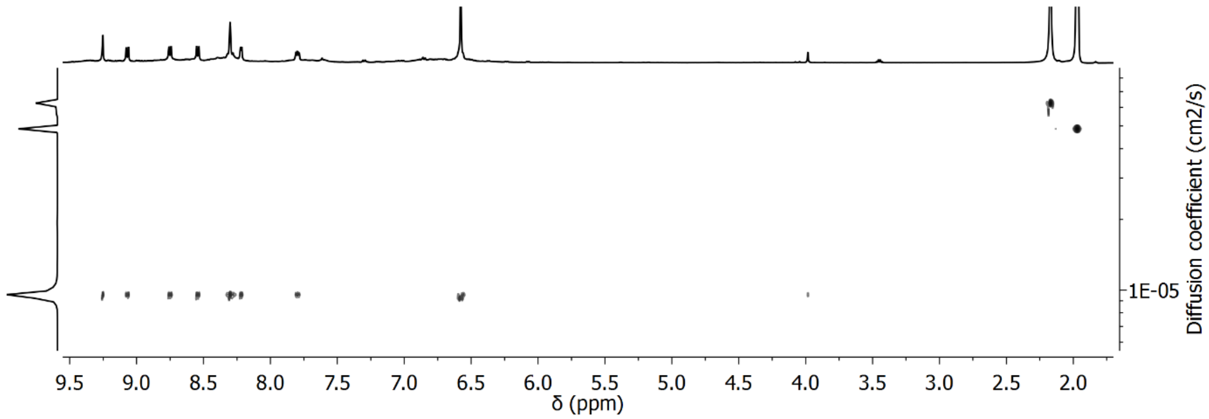

Figure S14. ^{1}H DOSY spectrum (500 MHz, 298 K, CD_3CN) of **2**. The diffusion coefficient was measured to be 9.5×10^{-6} cm^2 s^{-1}.

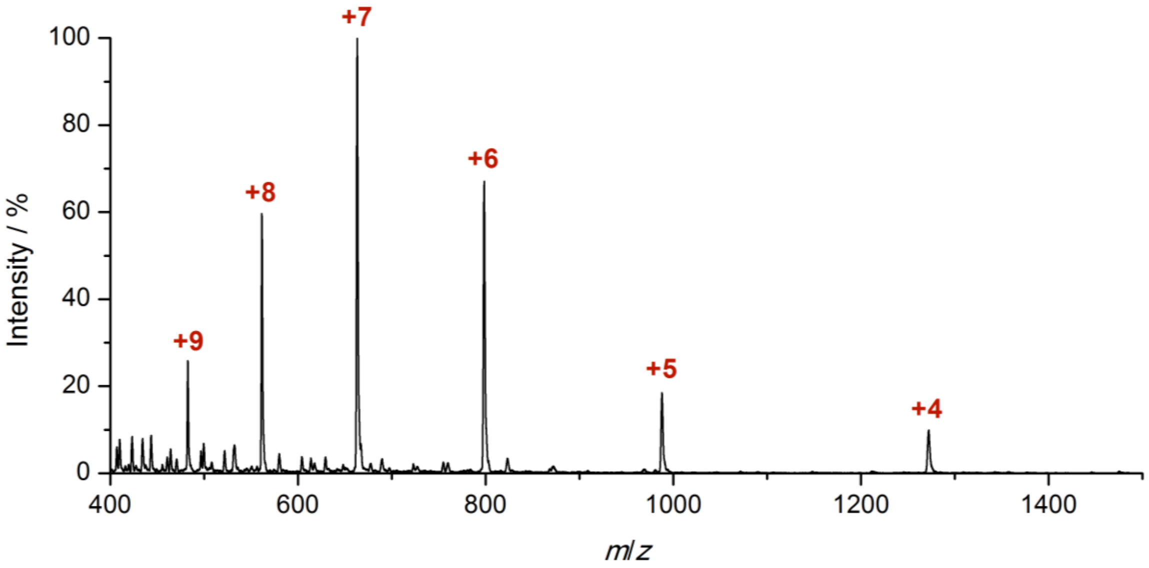

Figure S15. Low resolution ESI mass spectrum of **2**.

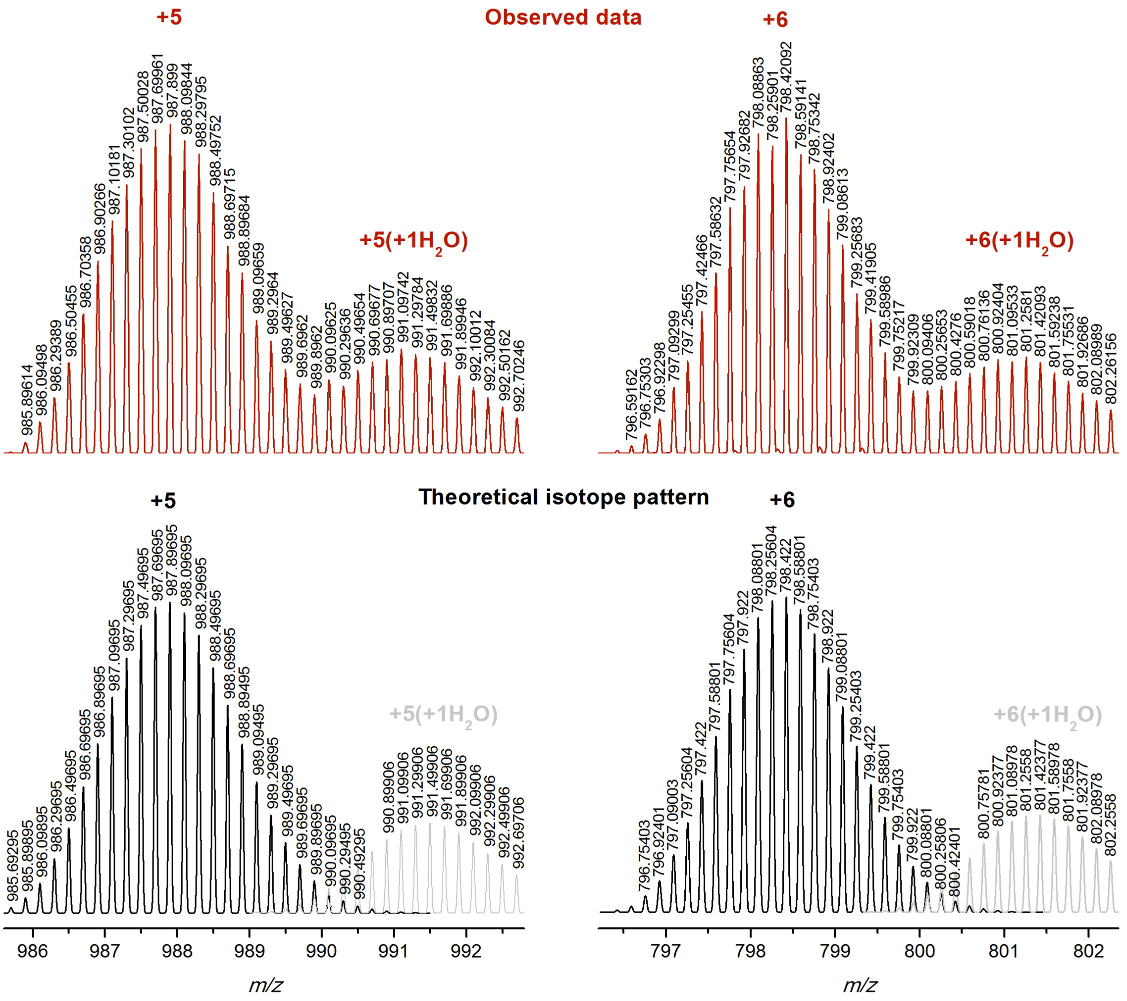

Figure S16. High resolution ESI mass spectra of **2**, showing the observed z = +5 and +6 charges (top), compared to the theoretical isotope patterns for each (bottom).

4. Preparation and characterisation of **3**

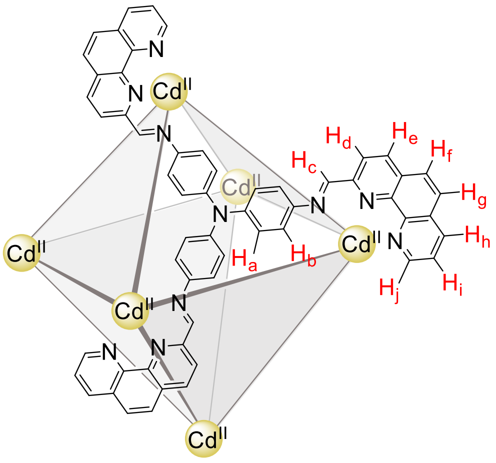

Tris(4-aminophenyl)amine (13.9 mg, 4.80 × 10^{-5} mol, 4 equiv.), 2-formylphenanthroline (30.0 mg, 1.44 × 10^{-5} mol, 12 eqiv.) and $Cd(OTf)_2$ (29.6 mg, 7.20 × 10^{-5} mol, 6 equiv.) were combined in CH_3CN and stirred at room temperature for 16 h. The solvent was evaporated and the solid triturated with THF (2 × 20 mL, or until the supernatant was colorless). The mixture was filtered and the residue dried *in vacuo* to yield a dark pink microcrystalline solid (58.5 mg, 9.91 × 10^{-6} mol, 83%).

^{1}H NMR (500 MHz, 298 K, CD_3CN): δ 9.71 (s, 12H, H$_c$), 9.18 (d, *J* = 8.3 Hz, 12H, H$_e$), 8.71 (d, *J* = 8.3 Hz, 12H, H$_d$), 8.54 (dd, *J* = 8.3, 1.6 Hz, 12H, H$_h$), 8.27 (d, *J* = 9.2 Hz, 12H, H$_f$), 8.17 (d, *J* = 9.2 Hz, 12H, H$_g$), 7.97 (dd, *J* = 4.8, 1.6 Hz, 12H, H$_j$), 7.57 (dd, *J* = 8.3, 4.8 Hz, 12H, H$_i$), 7.35 (d, *J* = 8.5 Hz, 24H, H$_b$), 6.69 (d, *J* = 8.5 Hz, 24H, H$_a$) ppm.

^{13}C NMR (125 MHz, 298 K, CD_3CN): *δ* 157.1, 151.3, 148.3, 147.3, 143.8, 141.5, 141.4, 141.1, 140.9, 132.8, 131.1, 130.4, 128.7, 127.7, 127.1, 125.7, 125.4, 123.3, 120.8 ppm.

LR-ESI-MS [charge, calculated for $C_{288}H_{144}N_{40}Cd_6(CF_3SO_3)_{12}$]: *m/z* = 1327.3 [**3**$(OTf)_8^{4+}$, 1327.7], 1032.3 [**3**$(OTf)_7^{5+}$, 1032.4], 835.4 [**3**$(OTf)_6^{6+}$, 835.5], 694.7 [**3**$(OTf)_5^{7+}$, 694.8], 589.2 [**3**$(OTf)_4^{8+}$, 589.3].

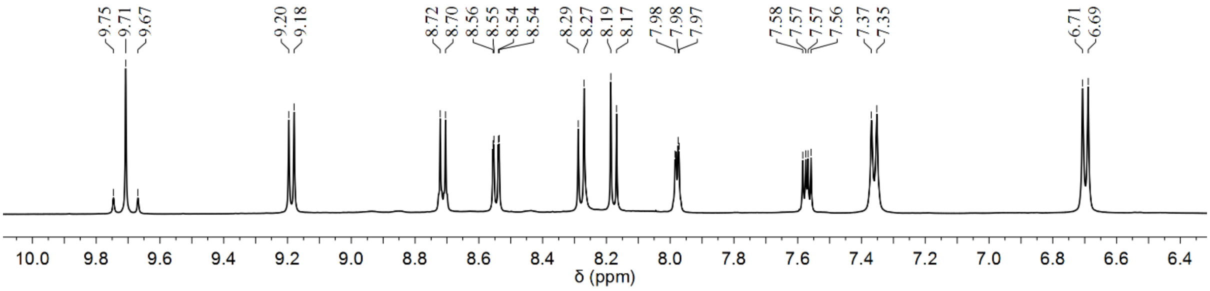

Figure S17. Aromatic region of the ^{1}H NMR spectrum (500 MHz, 298 K, CD_3CN) of **3**.

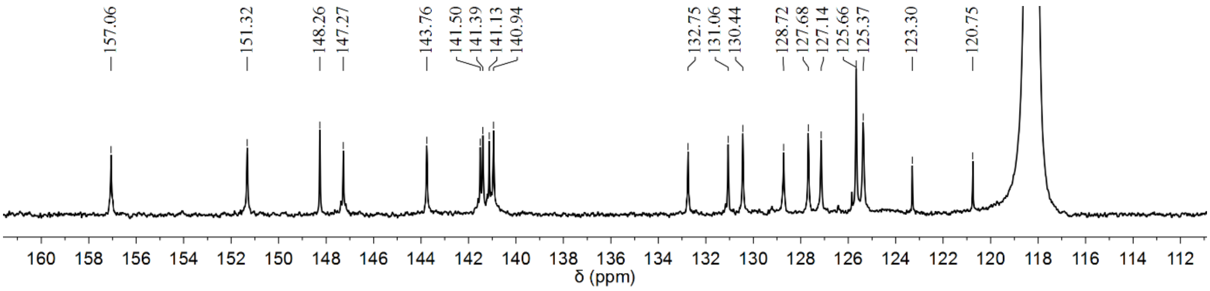

Figure S18. Aromatic region of the ^{13}C NMR spectrum (125 MHz, 298 K, CD_3CN) of **3**.

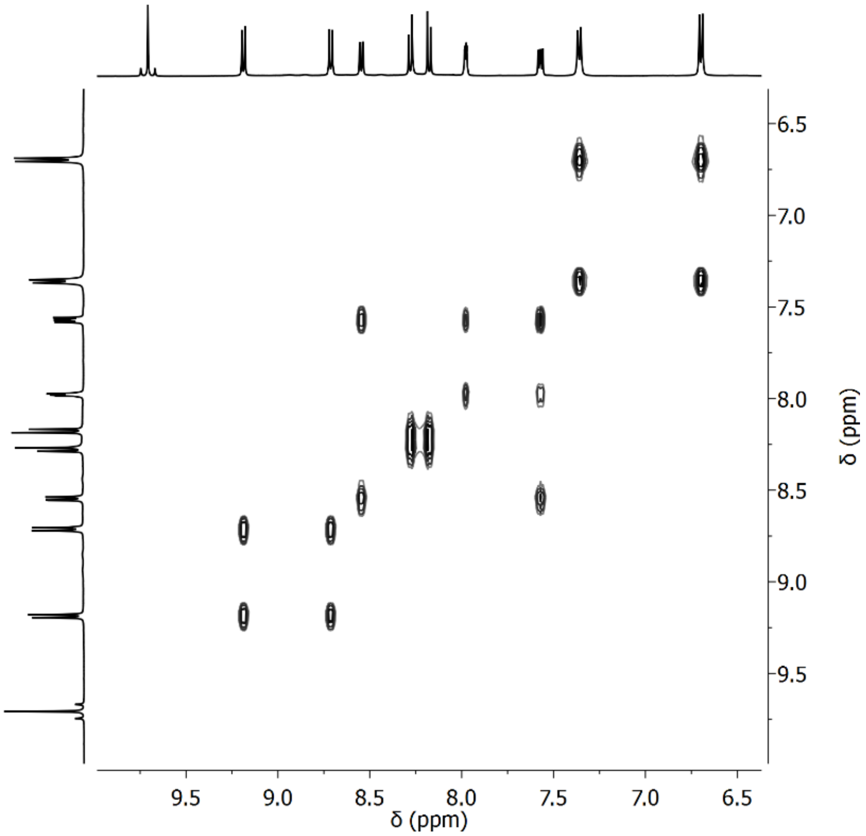

Figure S19. Aromatic region of the ^1H-^1H COSY spectrum (500 MHz, 298 K, CD_3CN) of **3**.

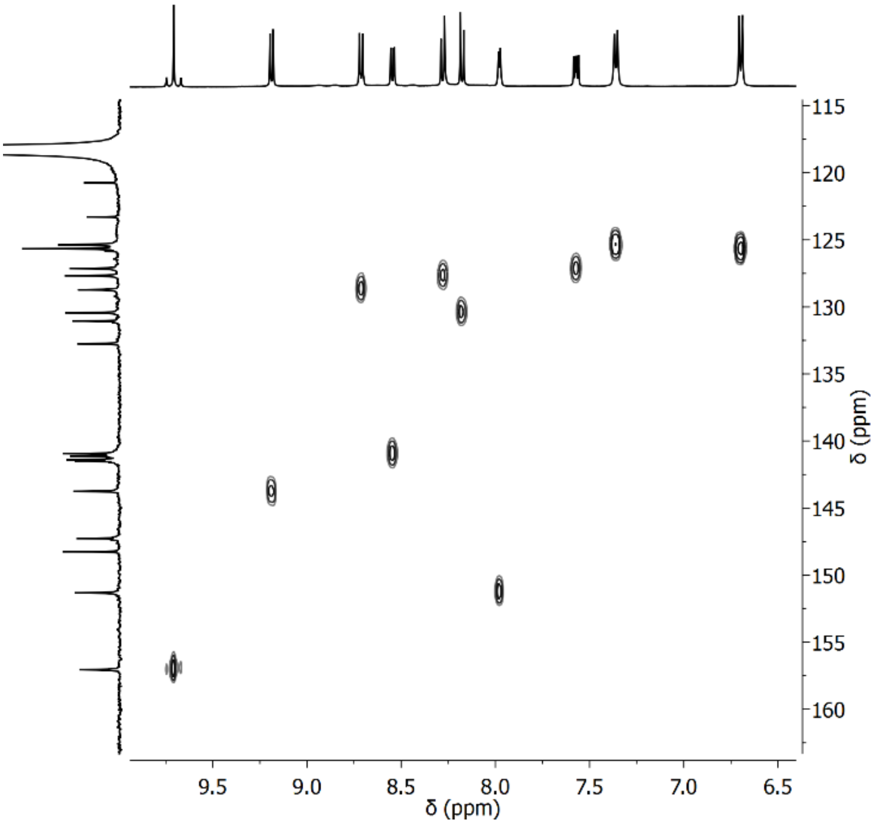

Figure S20. Aromatic region of the ^1H-^{13}C HSQC spectrum (500 MHz, 298 K, CD_3CN) of **3**.

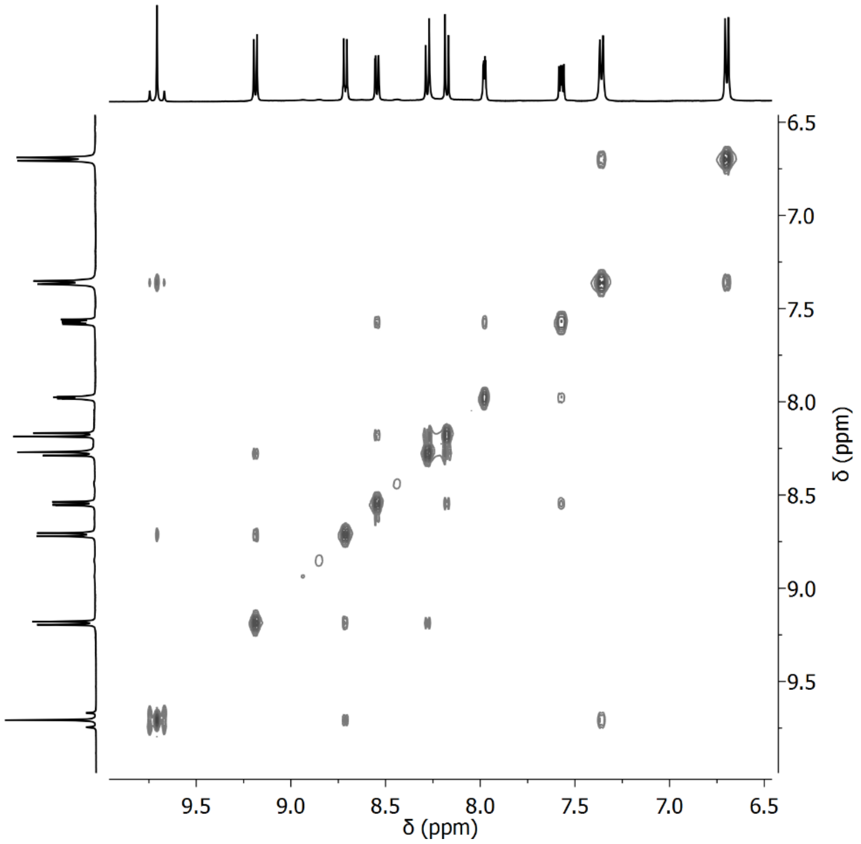

Figure S21. Aromatic region of the ^{1}H-^{1}H NOESY spectrum (500 MHz, 298 K, CD_3CN) of **3**.

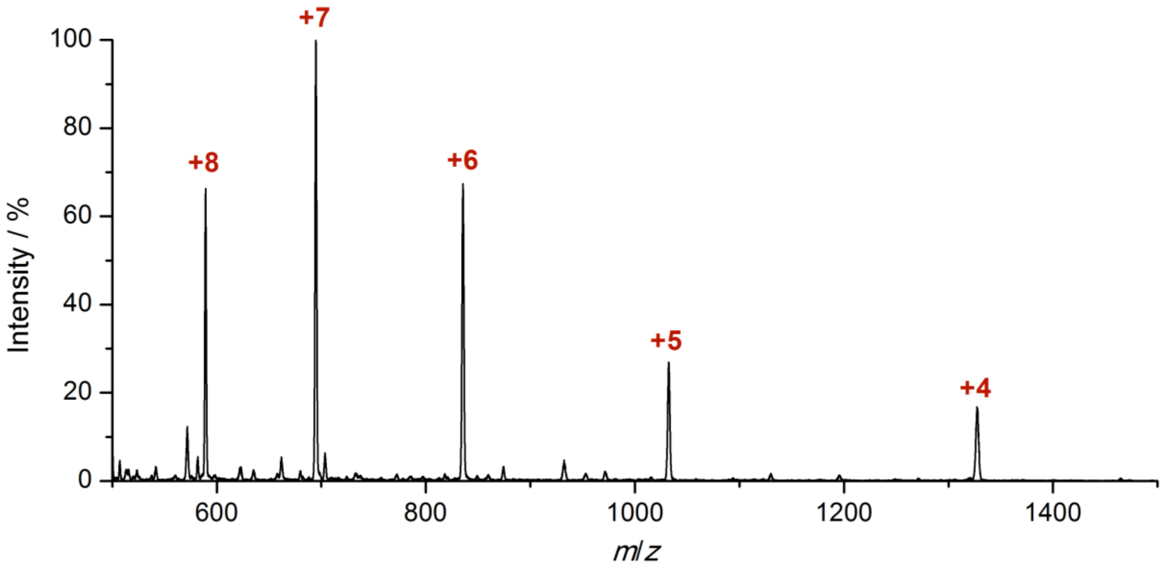

Figure S22. Low resolution ESI mass spectrum of **3**.

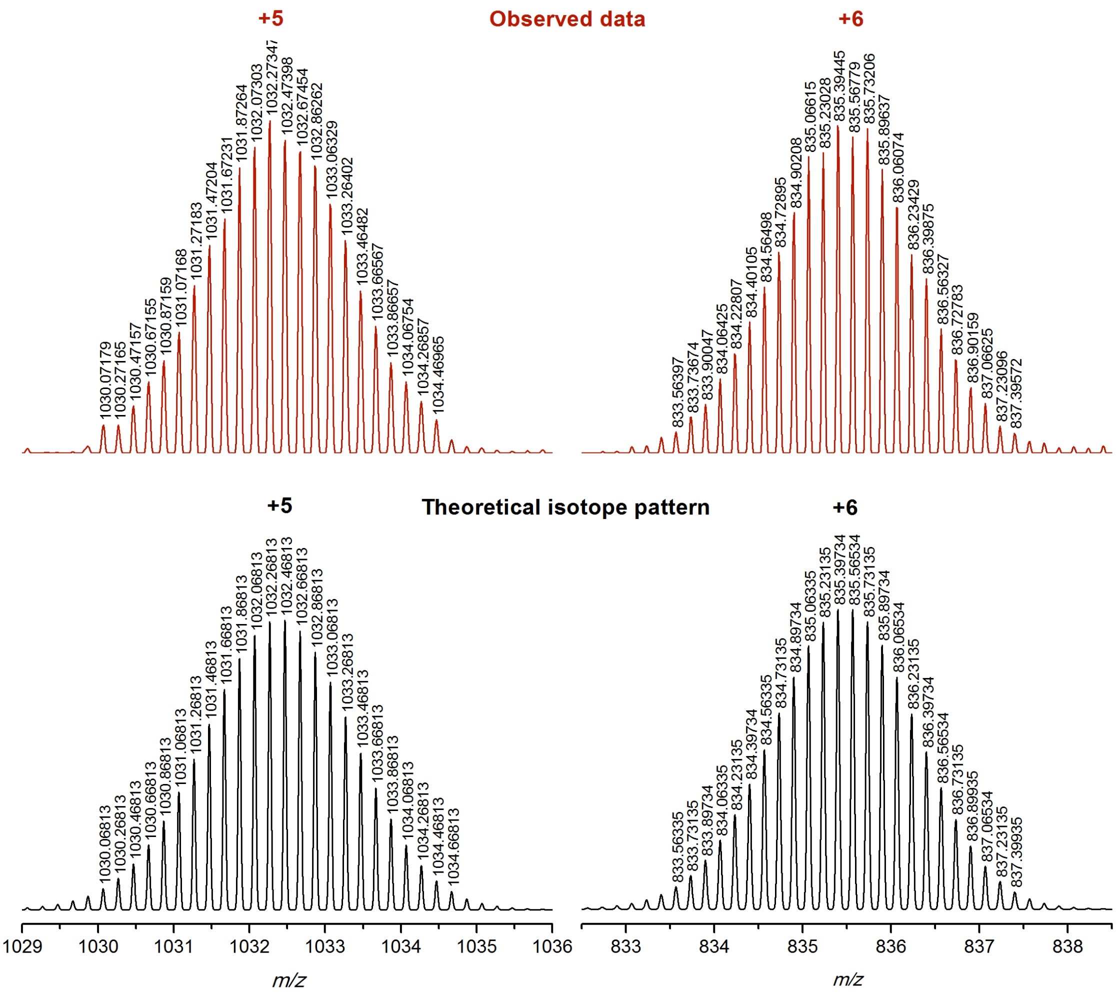

Figure S23. High resolution ESI mass spectra of **3**, showing the observed z = +5 and +6 charges (top), compared to the theoretical isotope patterns for each (bottom).

5. Crystallography

Data for **1** were collected using a Bruker D8 VENTURE diffractometer equipped with high-brilliance IμS Cu-Kα radiation (1.54178 Å), with ω and ψ scans at 180(2) K. Data for **2** were collected at Beamline I19 of Diamond Light Source employing silicon double crystal monochromated synchrotron radiation (0.6889 Å) with ω scans at 100(2) K. Data integration and reduction for **1** were undertaken with SAINT[4] in the APEX3 software suite; data integration and reduction for **2** were undertaken with Crysalis PRO.[5] In both instances, multi-scan empirical absorption corrections were applied to the data using SADABS.[6] Subsequent computations were carried out using the WinGX-32 graphical user interface.[7] Structures were solved by direct methods using SHELXT-2013[8] then refined and extended with SHELXL-2013.[8] In general, non-hydrogen atoms with occupancies greater than 0.5 were refined anisotropically. Carbon-bound hydrogen atoms were included in idealised positions and refined using a riding model. Disorder was modelled using standard crystallographic methods including constraints, restraints and rigid bodies where necessary. Crystallographic data along with specific details pertaining to the refinement follow. Crystallographic data have been deposited with the CCDC (CCDC 1456932 and 1456933).

5.1 Crystal structure of 1·12BF$_4$·5.25MeCN·0.5Et$_2$O

Formula $C_{240.50}H_{164.75}B_{12}F_{48}N_{45.25}O_{0.50}Zn_6$, *M* 5130.36, Triclinic, space group P-1 (#2), *a* 21.2908(17), *b* 34.428(3), *c* 40.373(3) Å, *α* 96.128(4), *β* 94.327(4), *γ* 101.218(5)°, *V* 28721(4) Å^3, D_c 1.186 g cm^{-3}, *Z* 4, crystal size 0.550 by 0.150 by 0.050 mm, colour purple, habit block, temperature 180(2) Kelvin, λ(CuKa) 1.54178 Å, μ(CuKa) 1.279 mm^{-1}, T(SADABS)$_{min,max}$ 0.5715, 0.7475, $2\theta_{max}$ 69.64, *hkl* range –15 15, –25 25, –29 29, *N* 134350, N_{ind} 24167(R_{merge} 0.0821), N_{obs} 16347(I > 2σ(I)), N_{var} 5805, residuals* $R1(F)$ 0.1598, $wR2(F^2)$ 0.4286, GoF(all) 1.141, $\Delta\rho_{min,max}$ –0.532, 0.966 e$^-$ Å^{-3}.

*$R1 = S||F_o| - |F_c||/S|F_o|$ for $F_o > 2s(F_o)$; $wR2 = (Sw(F_o^2 - F_c^2)^2/S(wF_c^2)^2)^{1/2}$ all reflections $w=1/[s^2(F_o^2)+(0.2000P)^2 +750.0000P]$ where $P=(F_o^2+2F_c^2)/3$

Specific refinement details

Crystals of **1·12BF$_4$·5.25MeCN·0.5Et$_2$O** were grown by slow diffusion of diethyl ether into an acetonitrile solution of **1**$(BF_4)_{12}$. The crystals employed rapidly lost solvent after removal from the mother liquor and rapid handling prior to flash cooling in the cryostream was required to collect data. Despite these measures and the use of high intensity laboratory source, few reflections at greater than 1.35 Å resolution were observed. Nevertheless, the quality of the data is more than sufficient to establish the connectivity of the structure. Due to the less than ideal resolution bond lengths and angles within pairs of organic ligands were restrained to be similar to each other and thermal parameter restraints (SIMU, DELU) were applied to all non-metal atoms to facilitate anisotropic refinement. Most tetrafluoroborate anions showed a significant amount of thermal motion; bond length and thermal parameter restraints were required for the realistic modelling of these anions. Some anions displayed positional disorder and were modelled over two (sometimes three) locations. All anions and solvent molecules were refined with isotropic thermal parameters. Three of the twenty-four anions present in the asymmetric unit could not be successfully resolved despite numerous attempts at modelling, including the use of rigid bodies. Consequently, the SQUEEZE[9] function of PLATON[10] was employed to remove the contribution of the electron density associated with the remaining anions and further highly disordered solvent molecules.

5.2 Crystal structure of $2\cdot12(CF_3SO_3)\cdot0.5MeCN$

Formula $C_{245}H_{149.50}F_{36}N_{36.50}O_{40}S_{12}Zn_6$, *M* 5705.44, Monoclinic, space group $P2_1/c$ (#14), *a* 39.1746(9), *b* 37.7597(8), *c* 44.3076(9) Å, *β* 103.755(2), *V* 63661(2) $Å^3$, D_c 1.191 g cm^{-3}, *Z* 8, crystal size 0.600 × 0.100 × 0.020 mm, colour purple, habit needle, temperature 100(2) Kelvin, *λ*(synchrotron) 0.6889 Å, *μ*(synchrotron) 0.556 mm^{-1}, $T(\text{SADABS})_{min,max}$ 0.2787, 0.7440, $2\theta_{max}$ 31.99, *hkl* range –31 31, –30 30, –30 31, *N* 370024, N_{ind} 31456(R_{merge} 0.1150), N_{obs} 23888(I > 2σ(I)), N_{var} 5123, residuals* $R1(F)$ 0.1986, $wR2(F^2)$ 0.4765, GoF(all) 1.066, $\Delta\rho_{min,max}$ –0.476, 0.862 e^- $Å^{-3}$.

*$R1 = S||F_o| - |F_c||/S|F_o|$ for $F_o > 2s(F_o)$; $wR2 = (Sw(F_o^2 - F_c^2)^2/S(wF_c^2)^2)^{1/2}$ all reflections $w=1/[s^2(F_o^2)+(0.2000P)^2+750.0000P]$ where $P=(F_o^2+2F_c^2)/3$

Specific refinement details

Crystals of **$2\cdot12(CF_3SO_3)\cdot0.5MeCN$** were grown by slow diffusion of diisopropylether into an acetonitrile solution of **2**$(OTf)_{12}$. The crystals employed rapidly lost solvent after removal from the mother liquor and rapid handling prior to flash cooling in the cryostream was required to collect data. Despite these measures and the use of synchrotron radiation, few reflections at greater than 1.2 Å resolution were observed. In addition, the crystals appeared to decay further during data collection, resulting in lower than ideal completeness and high residuals. Nevertheless, the quality of the data is more than sufficient to establish the connectivity of the structure. Due to the less than ideal resolution bond lengths and angles within pairs of organic ligands were restrained to be similar to each other and thermal parameter restraints (SIMU, DELU) were applied to all non-metal atoms to facilitate anisotropic refinement. Bond length and thermal parameter restraints were required for the realistic modelling of all triflate anions and solvent molecules; these were refined with isotropic thermal parameters. Only 5 anions could be successfully resolved in the asymmetric unit, despite numerous attempts at modelling, including the use of rigid bodies. Consequently, the SQUEEZE[9] function of PLATON[10] was employed to remove the contribution of the electron density associated with the remaining anions and further highly disordered solvent molecules.

6. VOIDOO calculations

In order to determine the available void space within **1-4**, VOIDOO calculations[11] were performed using MM2 minimized CACHE models (based on the crystal structures of **1** and **2**). A virtual probe with the minimum radius such that it would not exit the cavity of the largest structure (**4**) was employed for all cages. The following parameters were changed from their default values, following a previously published procedure.[12]

Probe radius: 3.3 Å

Primary grid spacing: 0.1

Maximum number of volume-refinement cycles: 30

Minimum size of secondary grid: 1

Grid for plot files: 0.2

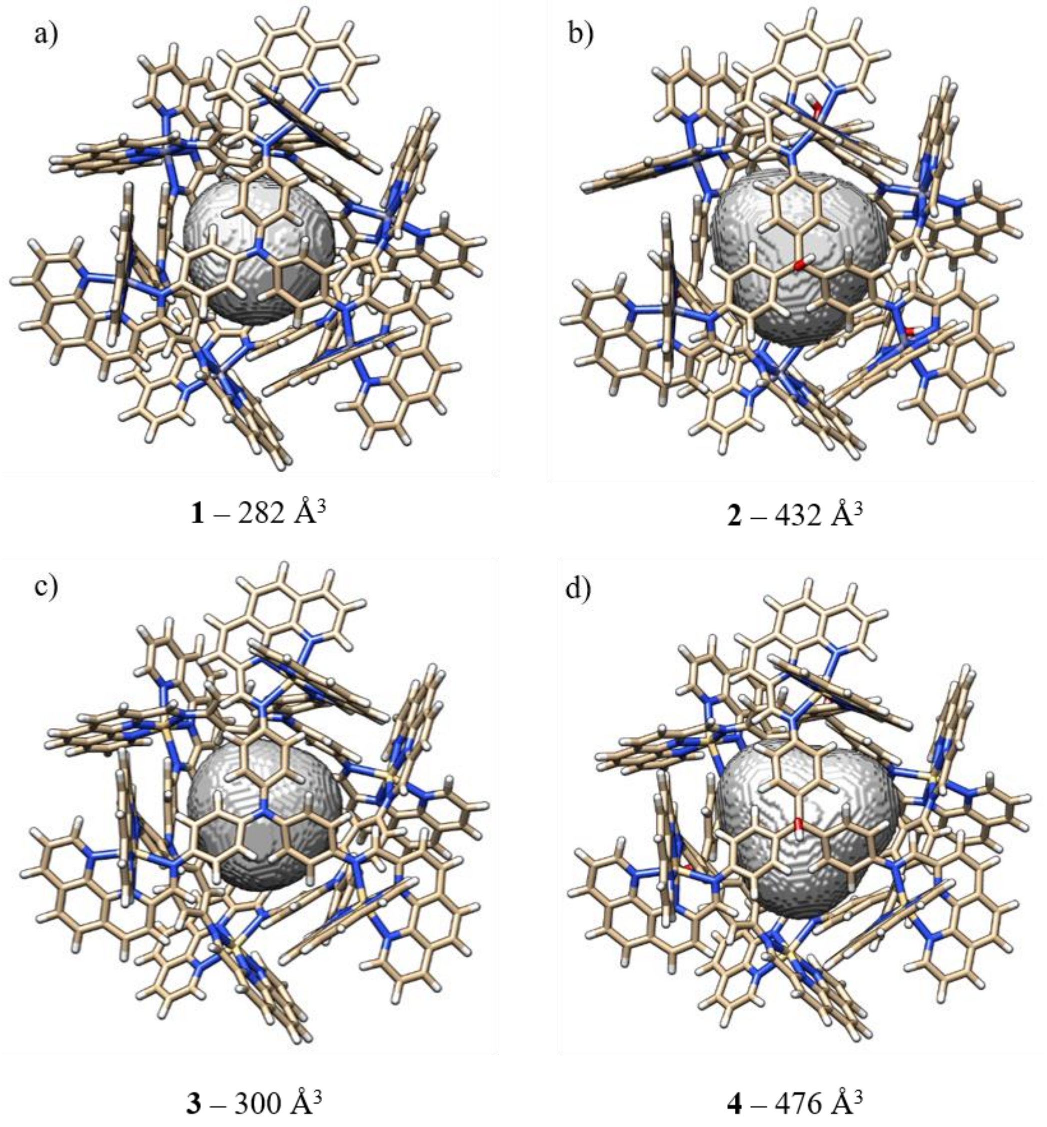

Figure S24. Void volumes calculated by VOIDOO (a) **1**, (b) **2**, (c) **3**, (d) **4**.

7. Host-guest chemistry of **1**

7.1 ^{n}Bu$_4$NBPh$_4$

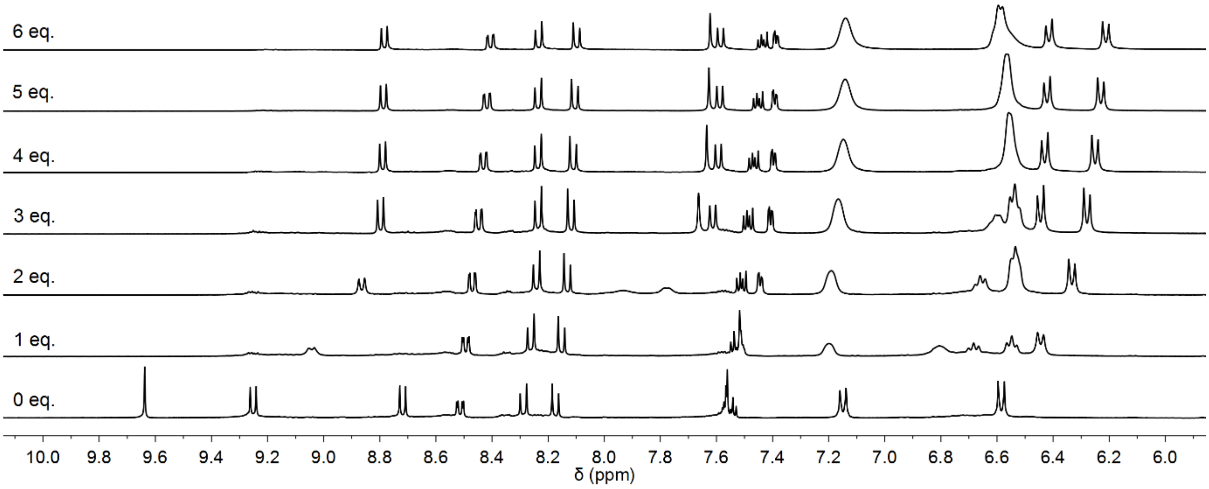

Figure S25. ^{1}H NMR titration (400 MHz, 298 K) of ^{n}Bu$_4$NBPh$_4$ into a solution of **1** in CD$_3$CN (equivalents of anion are labeled on individual spectra).

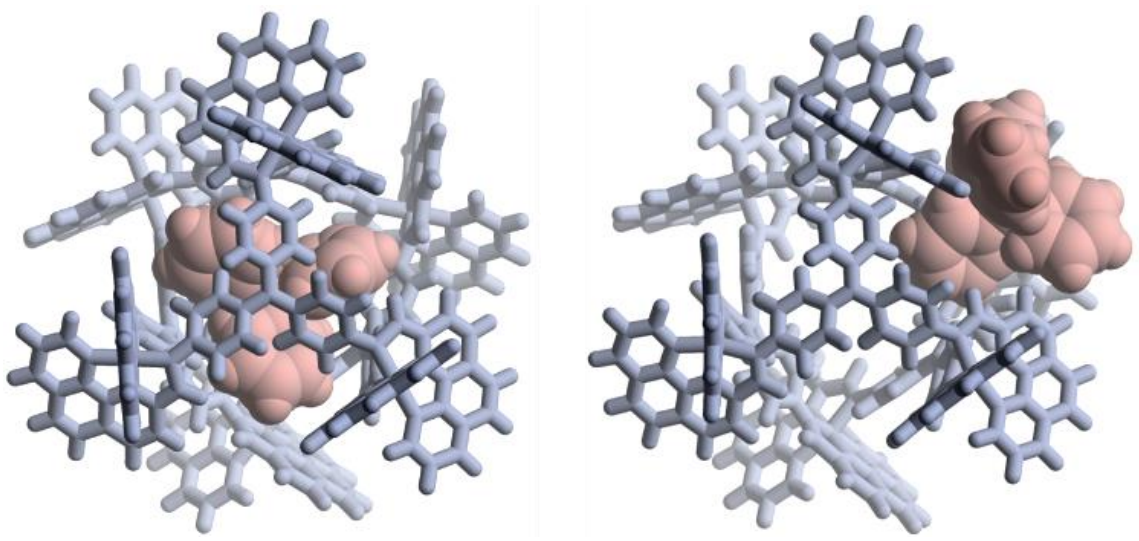

Figure S26. MM2 molecular models of BPh$_4^{-}$•**1** showing the two potential binding sites for BPh$_4^{-}$ guests: interior (left) and exterior (right). During interior binding, the *ortho* proton of BPh$_4^{-}$ would not exhibit NOEs to the imine or phenanthroline protons. Conversely, close contacts (2.5-4 Å) can be observed between the *ortho* proton of BPh$_4^{-}$ and the phenyl, imine and proximal phenanthroline protons of **1** during exterior binding (left). These are reflected in the NOESY/ROESY experiments (Figure S27).

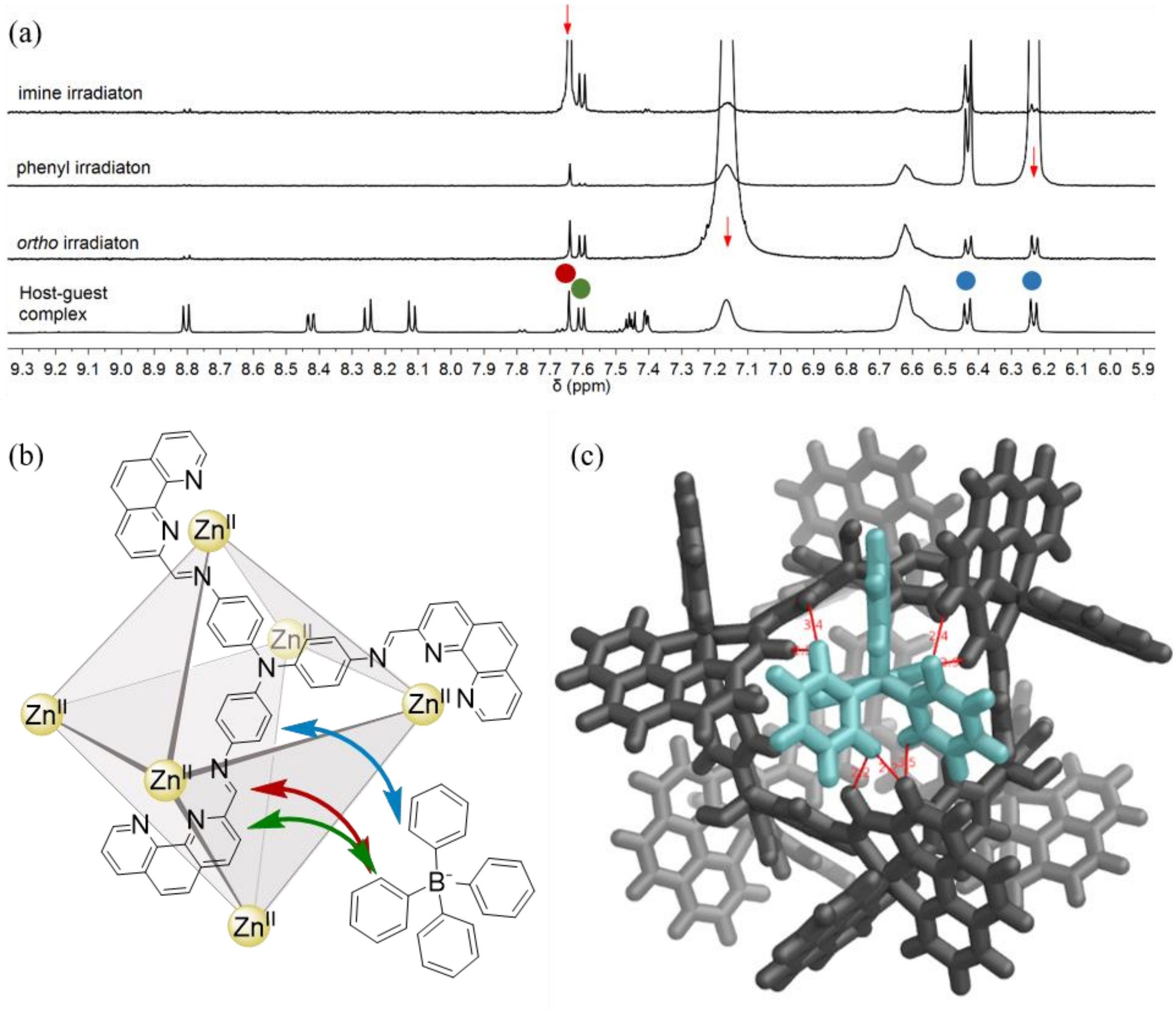

Figure S27. (a) 1D selective ^1H NOESY spectra (500 MHz, CD_3CN, 298 K) for BPh_4^-•**1**, individually irradiating the *ortho* proton of the BPh_4^- guest, the phenyl protons of **A**, or the imine protons of **1**. Red arrows indicate the excitation resonance for each spectrum. Colored circles indicate the cage environments that display NOEs to the *ortho* proton of BPh_4^-, which correspond to the colored arrows marked in (b). (c) MM2 molecular model of BPh_4^-•**1** highlighting the closest cage environments to the *ortho* proton of BPh_4^- (both interiorly and exteriorly orientated phenyl rings) during exterior binding. The closest interactions (2.3-3.5 Å, marked in red) are to the imine, phenyl and proximal phenanthroline protons, indicating an exterior binding mode of BPh_4^-.

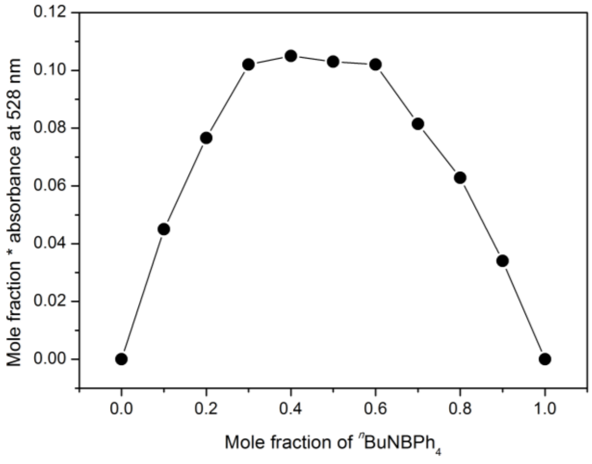

Figure S28. Job plot of $^nBu_4NBPh_4$ with **1** in CH_3CN to determine the 1:1 binding stoichiometry, measured by UV-Vis spectroscopy.

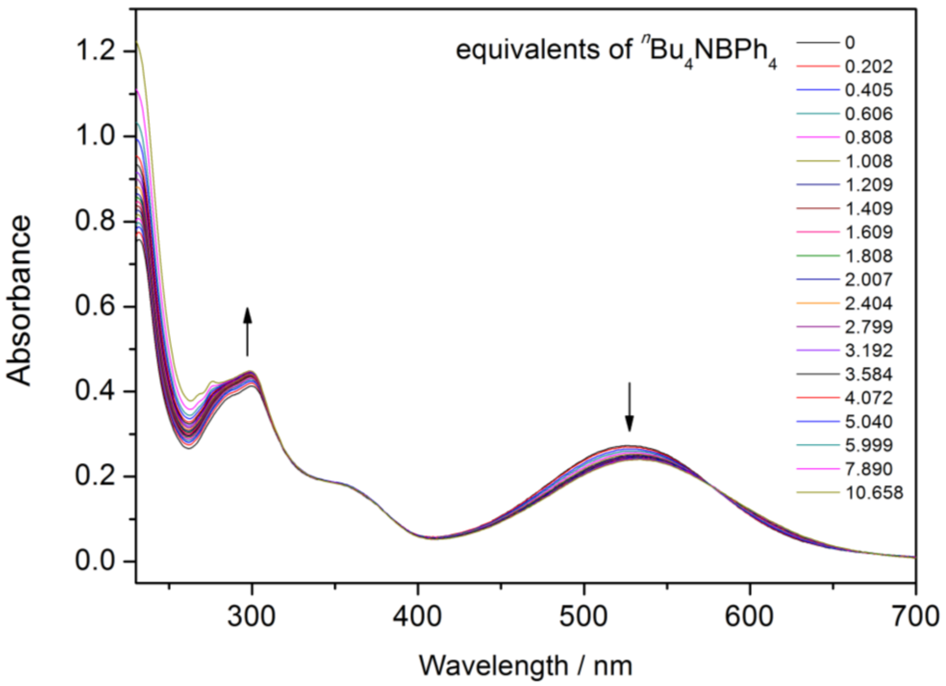

Figure S29. UV-Vis titration of ^{n}Bu$_4$NBPh$_4$ into a solution of **1** in CH_3CN at 298 K.

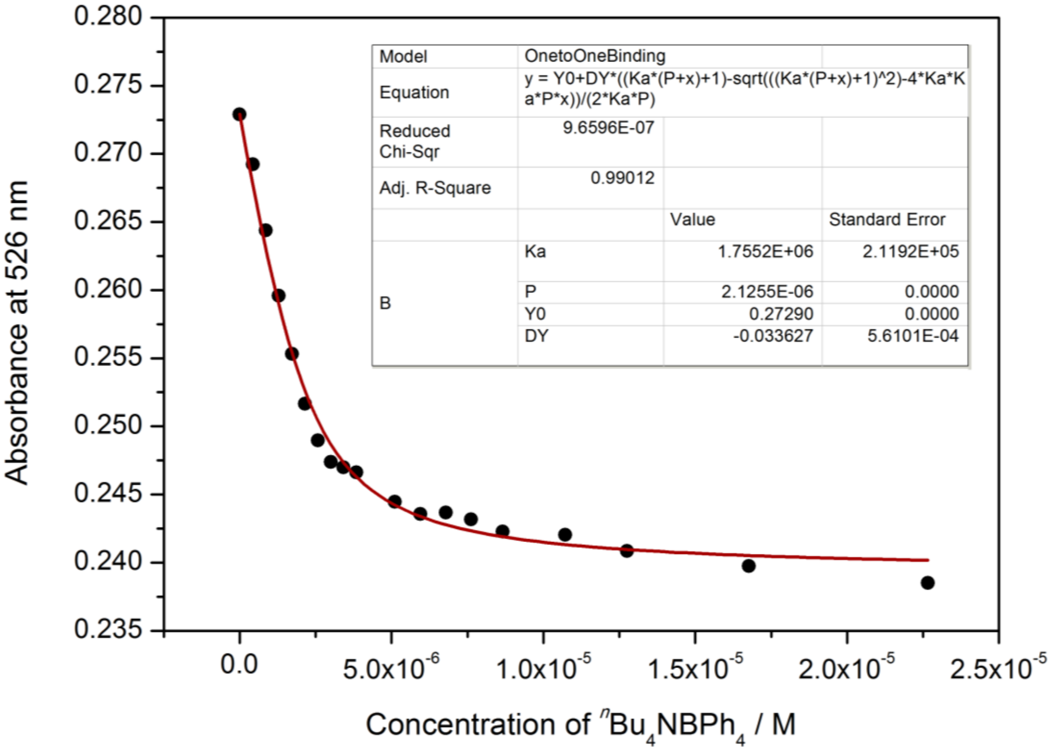

Figure S30. Binding isotherm (1:1 system) fit to the absorbance of **1** at 526 nm *vs.* the concentration of ^{n}Bu$_4$NBPh$_4$ added to determine binding affinity (K_a).

7.2 Na[B(C_6H_4F)$_4$]

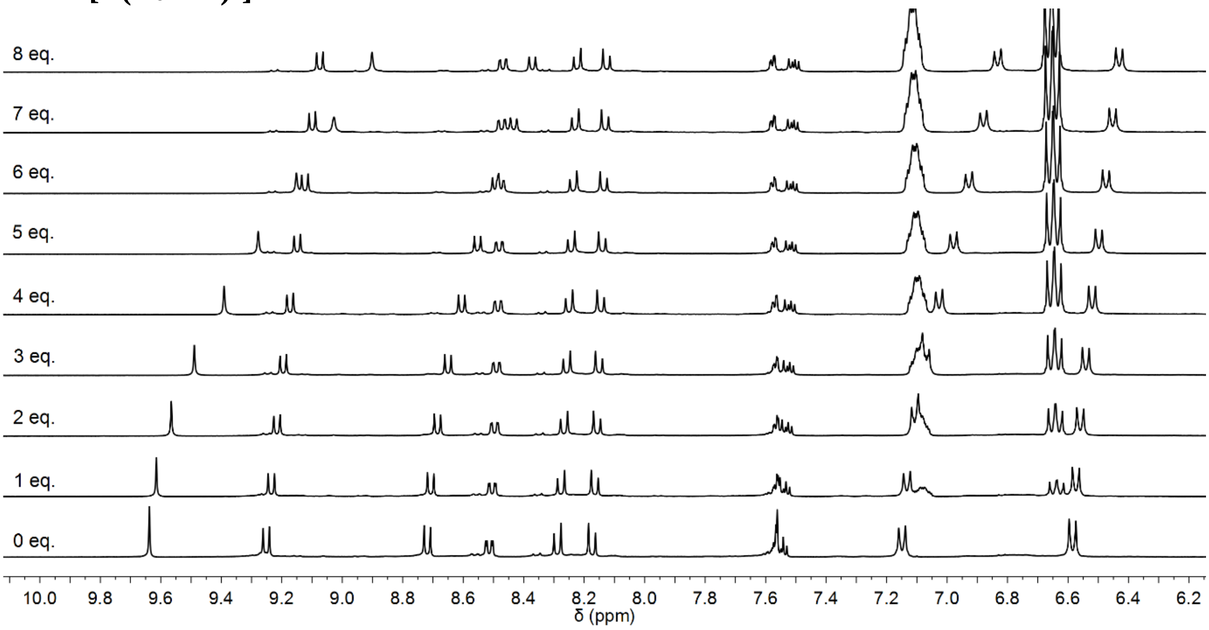

Figure S31. ^{1}H NMR titration (400 MHz, 298 K) of Na[B(C_6H_4F)$_4$] into a solution of **1** in CD_3CN (equivalents of anion are labeled on individual spectra).

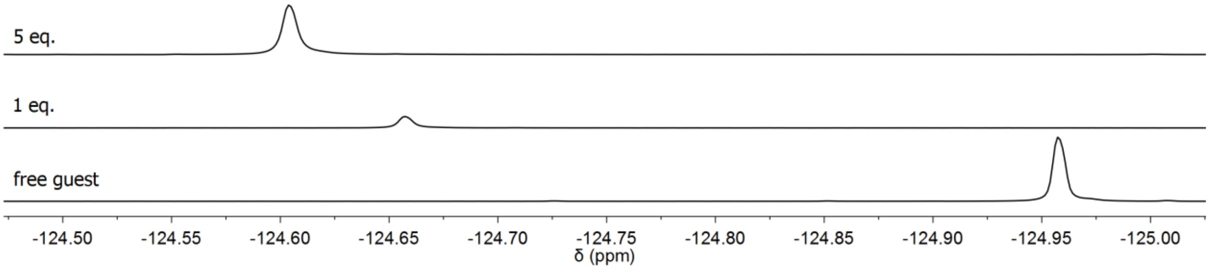

Figure S32. ^{19}F NMR spectra (376 MHz, 298 K, CD_3CN) of **1** with 1 and 5 equivalents of Na[B(C_6H_4F)$_4$], compared to the ^{19}F NMR spectrum of the free B(C_6H_4F)$_4^-$ anion.

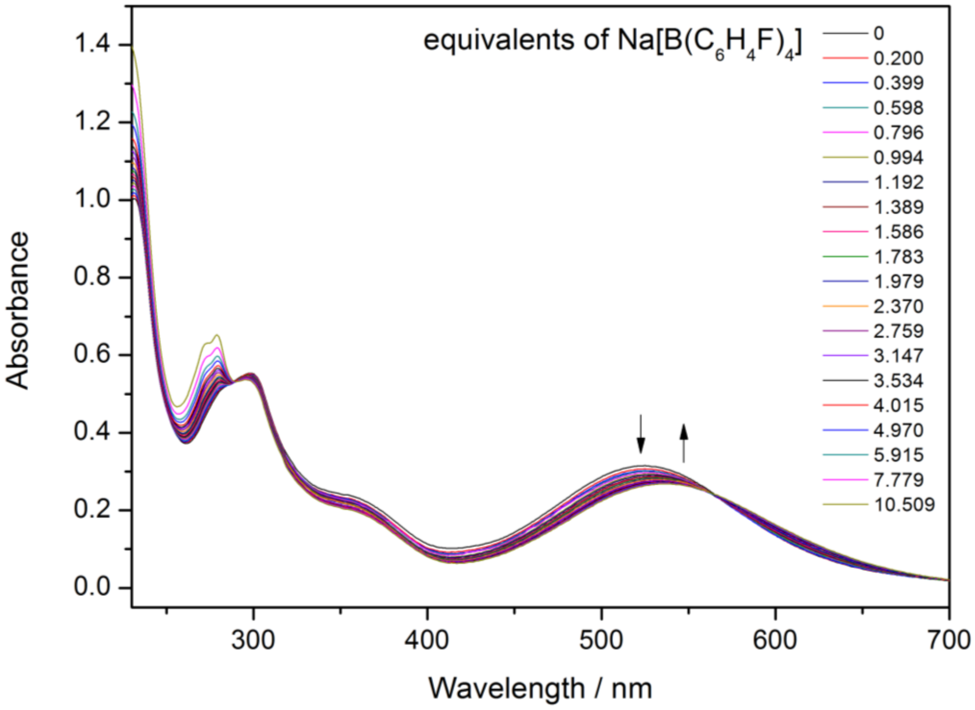

Figure S33. UV-Vis titration of $Na[B(C_6H_4F)_4]$ into a solution of **1** in CH_3CN at 298 K.

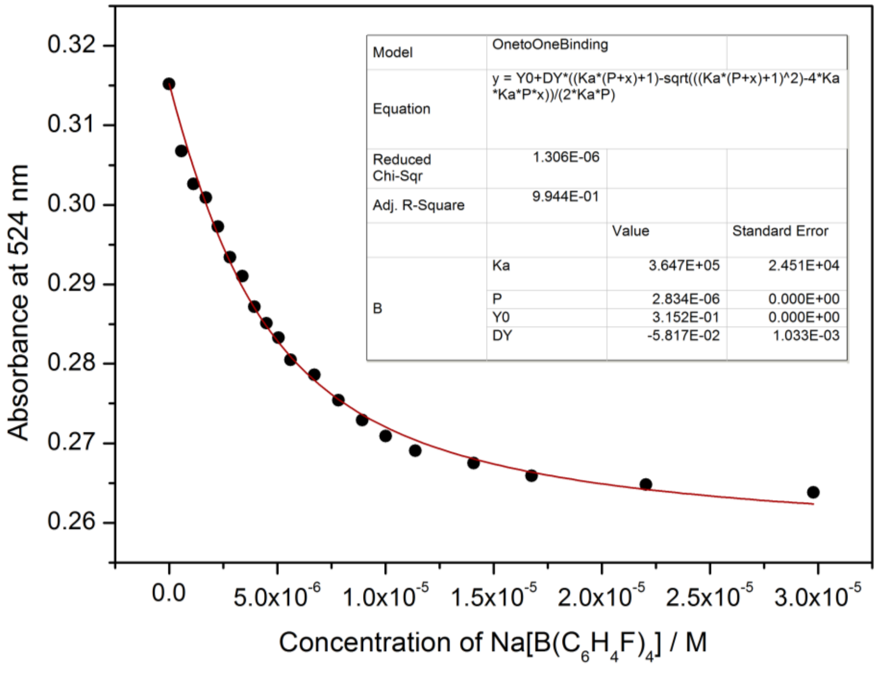

Figure S34. Binding isotherm (1:1 system) fit to the absorbance of **1** at 524 nm *vs.* the concentration of $Na[B(C_6H_4F)_4]$ added to determine binding affinity (K_a).

7.3 K[B(C_6H_4Cl)$_4$]

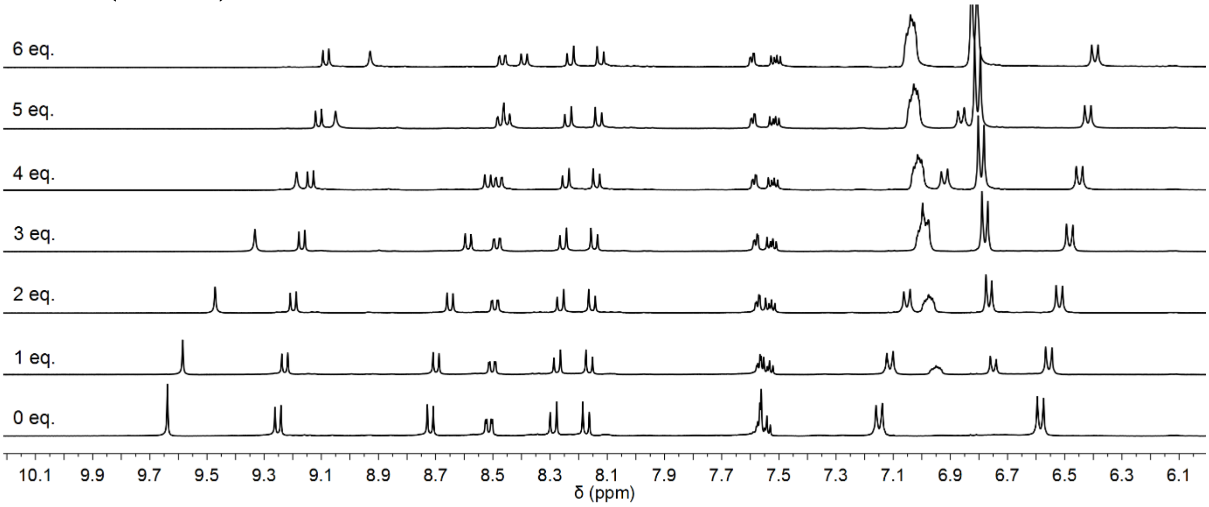

Figure S35. ^{1}H NMR titration (400 MHz, 298 K) of K[B(C_6H_4Cl)$_4$] into a solution of **1** in CD_3CN (equivalents of anion are labeled on individual spectra).

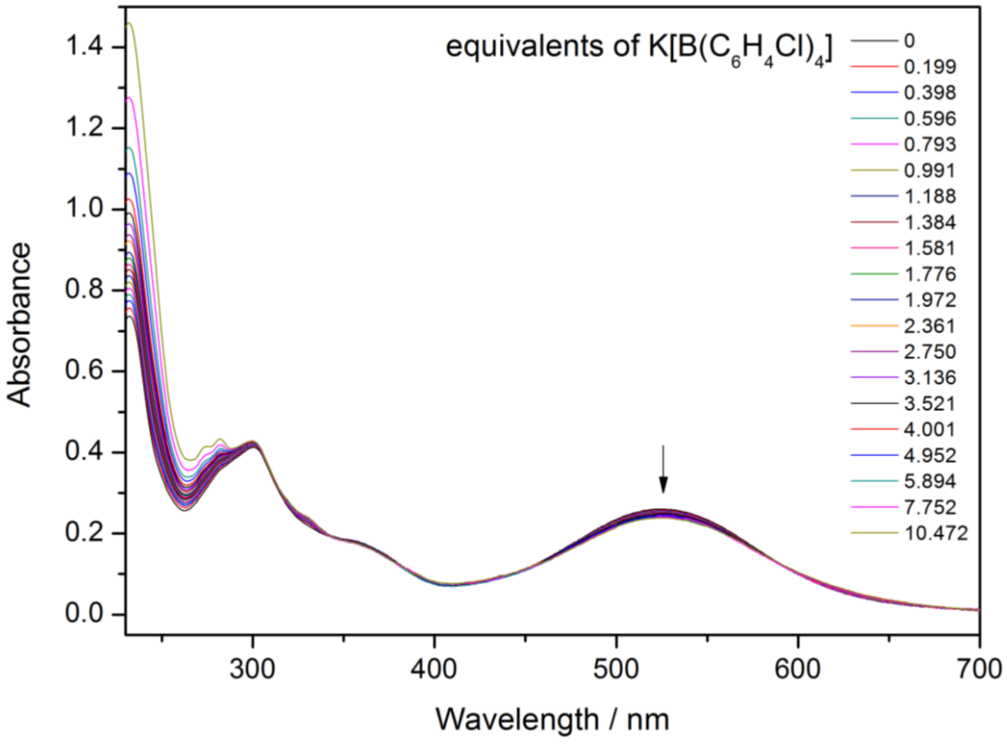

Figure S36. UV-Vis titration of K[B(C_6H_4Cl)$_4$] into a solution of **1** in CH_3CN at 298 K.

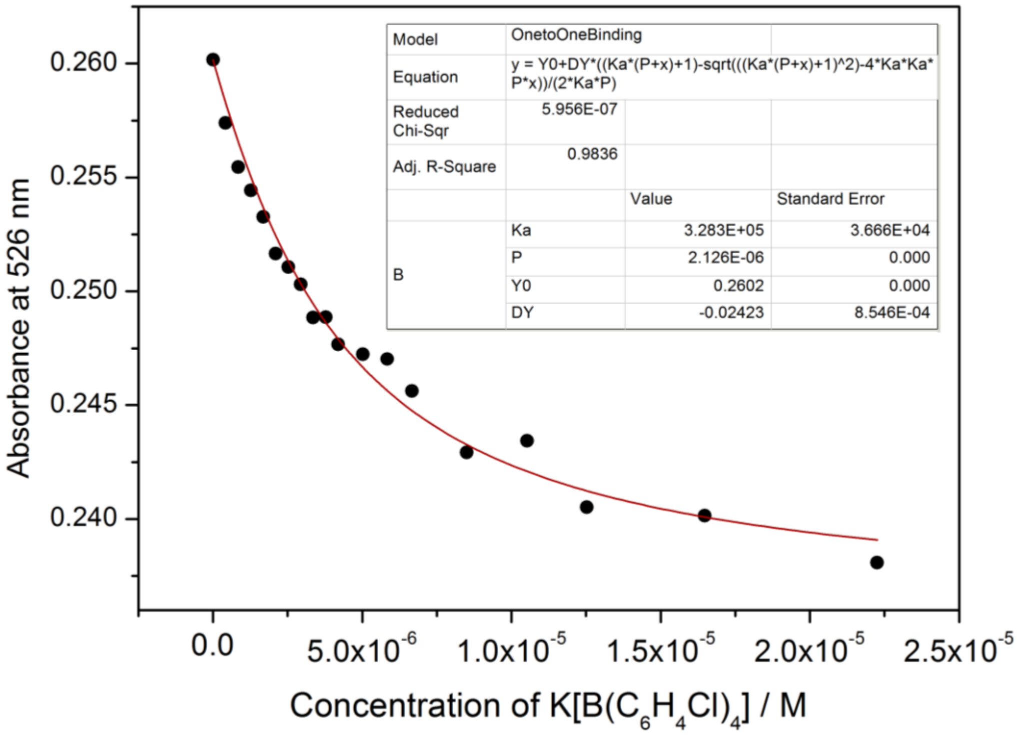

Figure S37. Binding isotherm (1:1 system) fit to the absorbance of **1** at 526 nm *vs.* the concentration of $K[B(C_6H_4Cl)_4]$ added to determine binding affinity (K_a).

8. Host-guest chemistry of **2**

8.1 ^{n}Bu$_4$NBPh$_4$

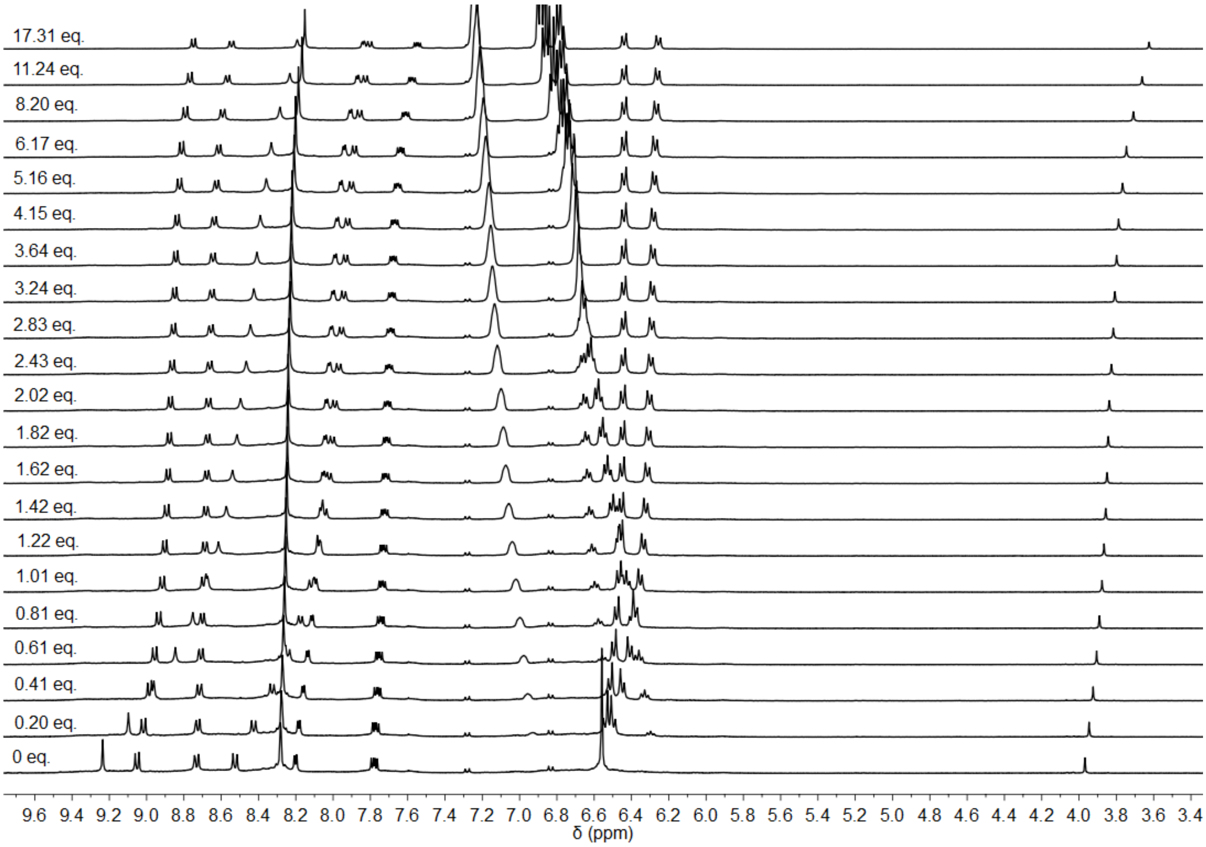

Figure S38. ^{1}H NMR titration (400 MHz, 298 K) of ^{n}Bu$_4$NBPh$_4$ into a solution of **2** in CD$_3$CN (equivalents of anion are labeled on individual spectra).

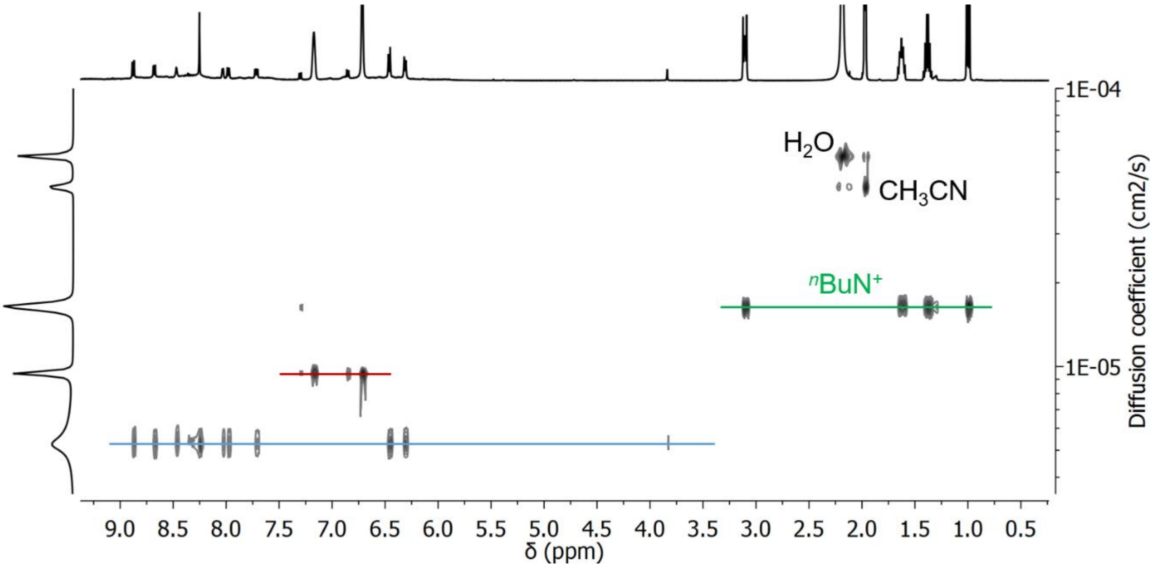

Figure S39. ^{1}H DOSY spectrum (500 MHz, 298 K, CD$_3$CN) of BPh$_4^-$•**2**. The diffusion coefficient of cage **2** was measured to be 5.2 × 10^{-6} cm^2 s^{-1} (blue). The occluded BPh$_4^-$ displayed a diffusion coefficient of 9.4 x 10^{-6} cm^2 s^{-1} (red), slower than the literature value for free BPh$_4^-$ in CD$_3$CN (1.7 x 10^{-5} cm^2 s^{-1} [13]).

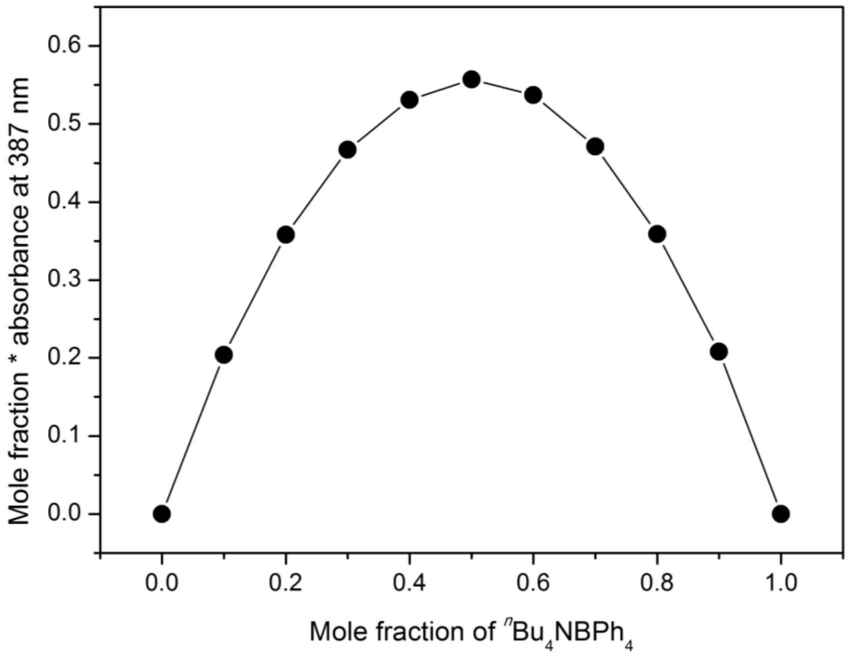

Figure S40. Job plot of $^{n}Bu_4NBPh_4$ with **2** in CH_3CN to determine the 1:1 binding stoichiometry, measured by UV-Vis spectroscopy.

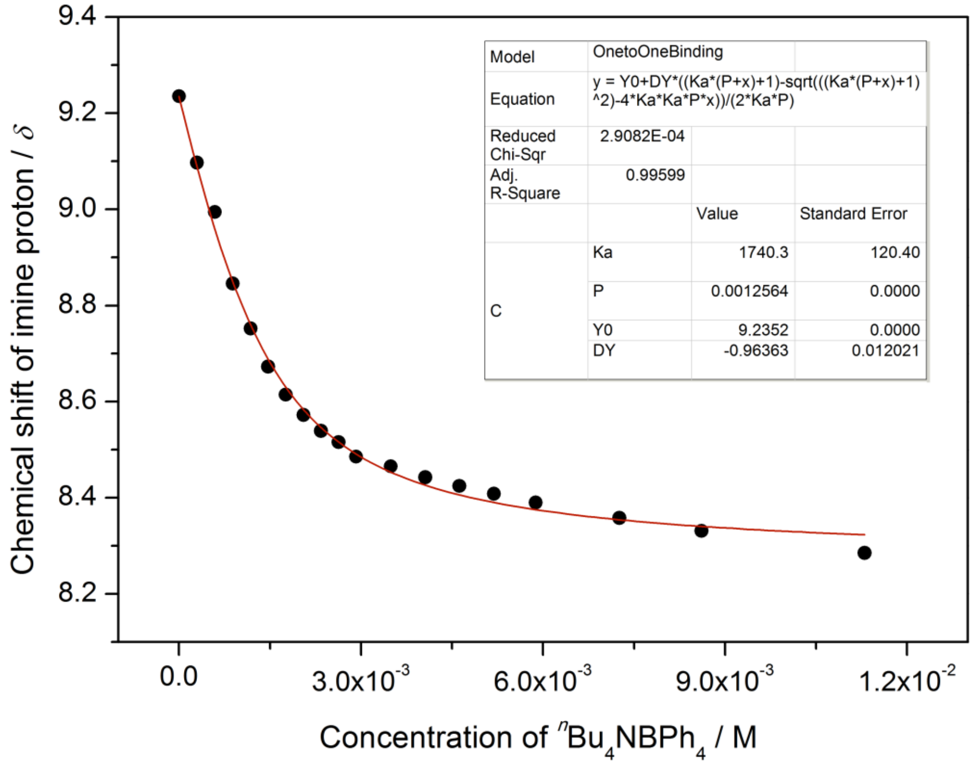

Figure S41. Binding isotherm (1:1 system) fit to the chemical shift of the imine proton *vs.* the concentration of $^{n}Bu_4NBPh_4$ added to determine binding affinity (K_a).

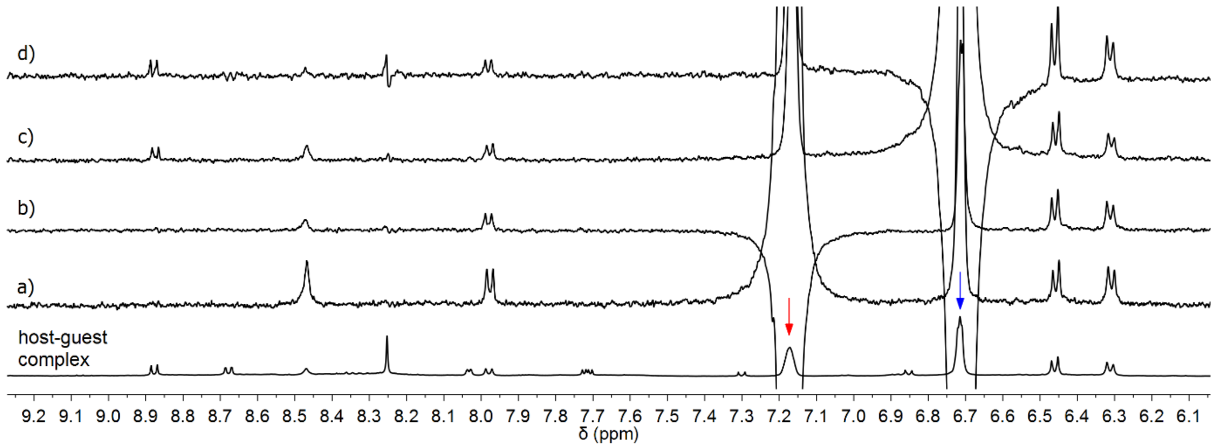

Figure S42. 1D selective ^{1}H NOESY and ROESY experiments (500 MHz, 298 K, CD_3CN) on BPh_4^-•**2**: a) ^{1}H NOESY and b) ^{1}H ROESY spectra irradiated at the *ortho* position; c) ^{1}H NOESY and d) ^{1}H ROESY spectra irradiated at the *meta/para* position (red arrow = *ortho* protons, blue arrow = coincident *meta/para* protons).

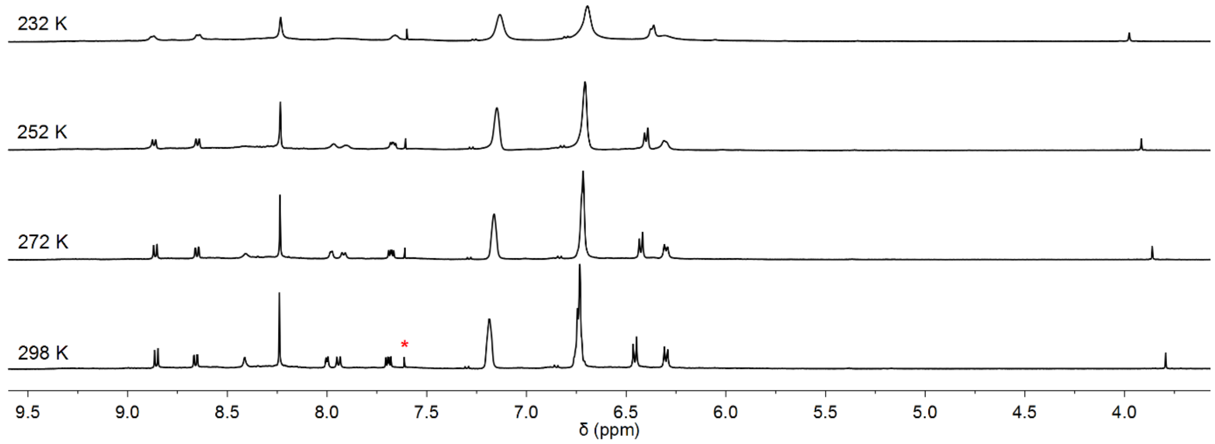

Figure S43. Variable temperature ^{1}H NMR spectra (500 MHz) of BPh_4^-•**2** in CD_3CN (asterisk marks a small portion of $CHCl_3$; spectra are labeled at individual temperatures). Broadening of the environments closest to BPh_4^-, rather than spectral desymmetrization, was observed for the host-guest species.

8.2 Na[B(C_6H_4F)$_4$]

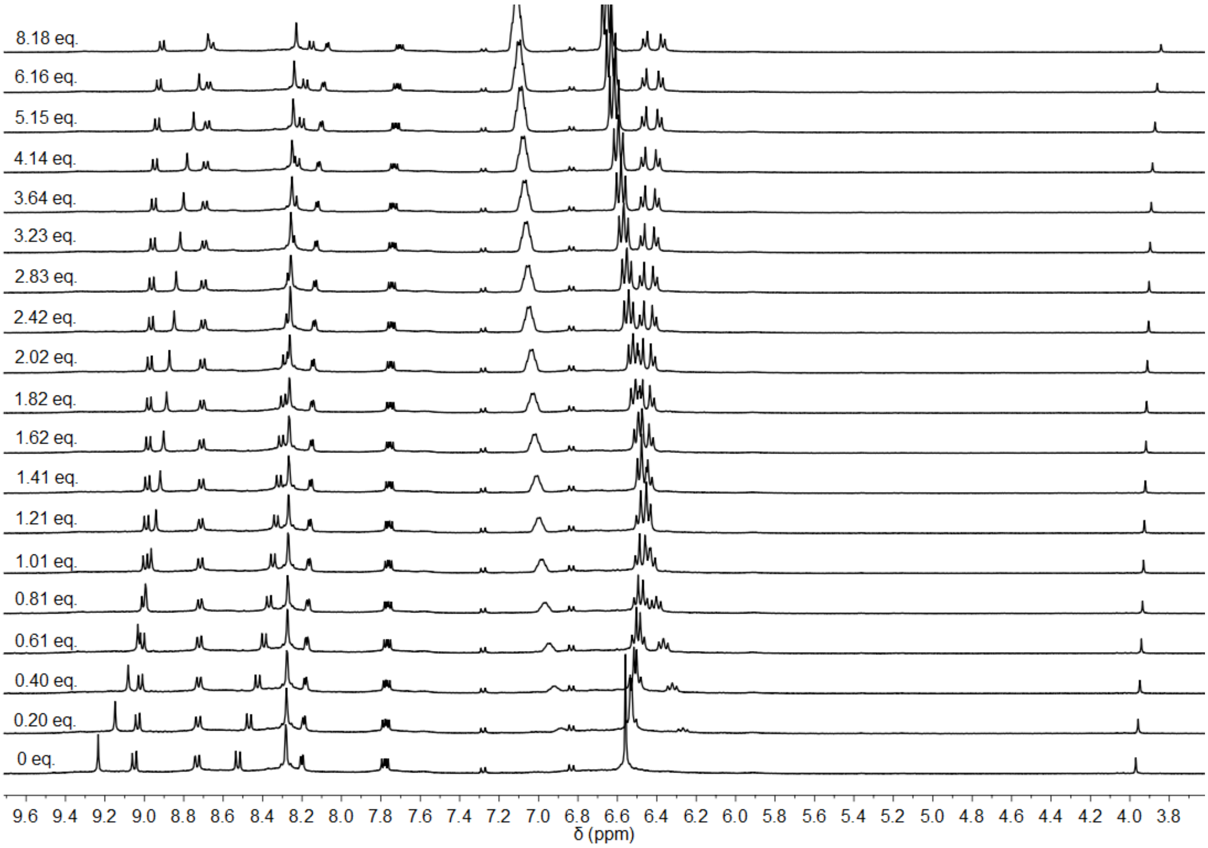

Figure S44. ^{1}H NMR titration (400 MHz, 298 K) of Na[B(C_6H_4F)$_4$] into a solution of **2** in CD_3CN (equivalents of anion are labeled on individual spectra).

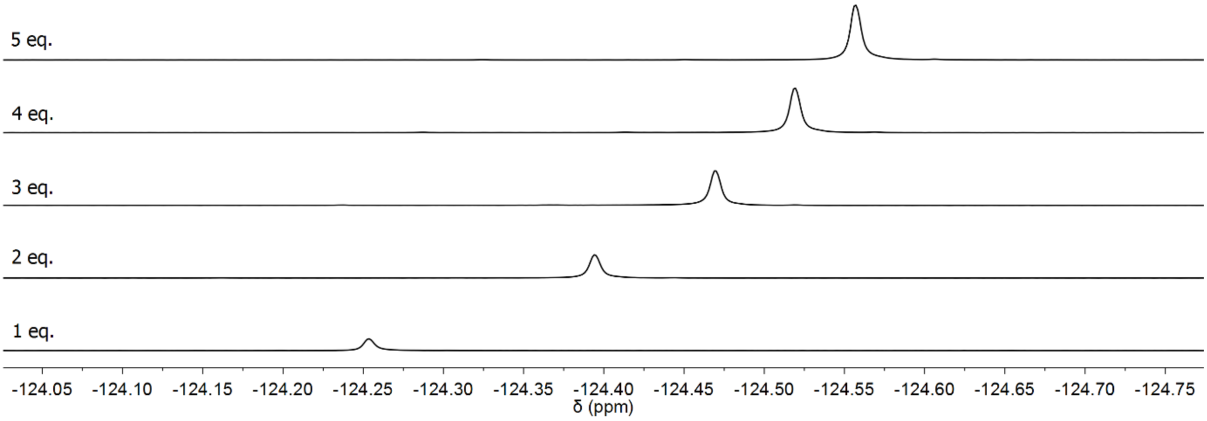

Figure S45. ^{19}F NMR titration (376 MHz, 298 K) of Na[B(C_6H_4F)$_4$] into a solution of **2** in CD_3CN (equivalents of anion are labeled on individual spectra).

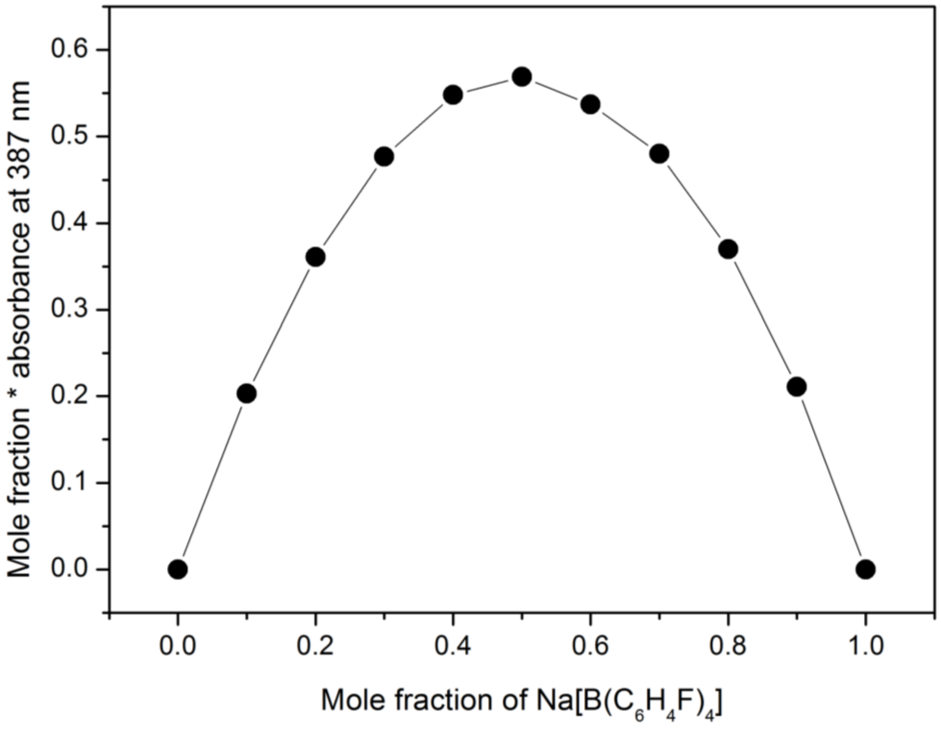

Figure S46. Job plot of $Na[B(C_6H_4F)_4]$ with **2** in CH_3CN to determine the 1:1 binding stoichiometry, measured by UV-Vis spectroscopy.

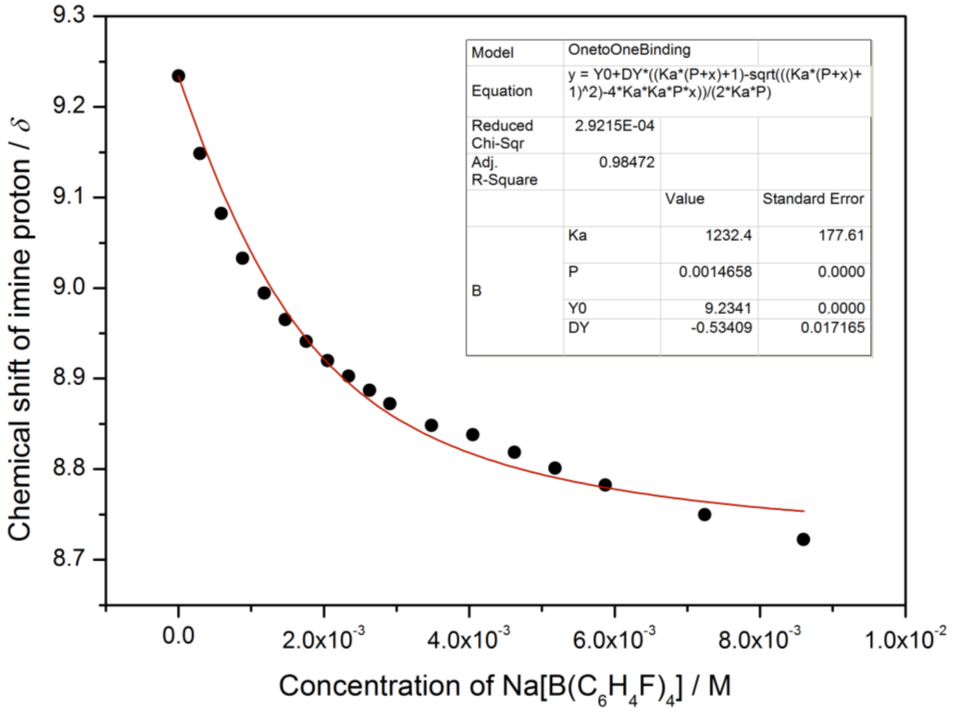

Figure S47. Binding isotherm (1:1 system) fit to the chemical shift of the imine proton *vs.* the concentration of $Na[B(C_6H_4F)_4]$ added to determine binding affinity (K_a).

8.3 K[B(C_6H_4Cl)$_4$]

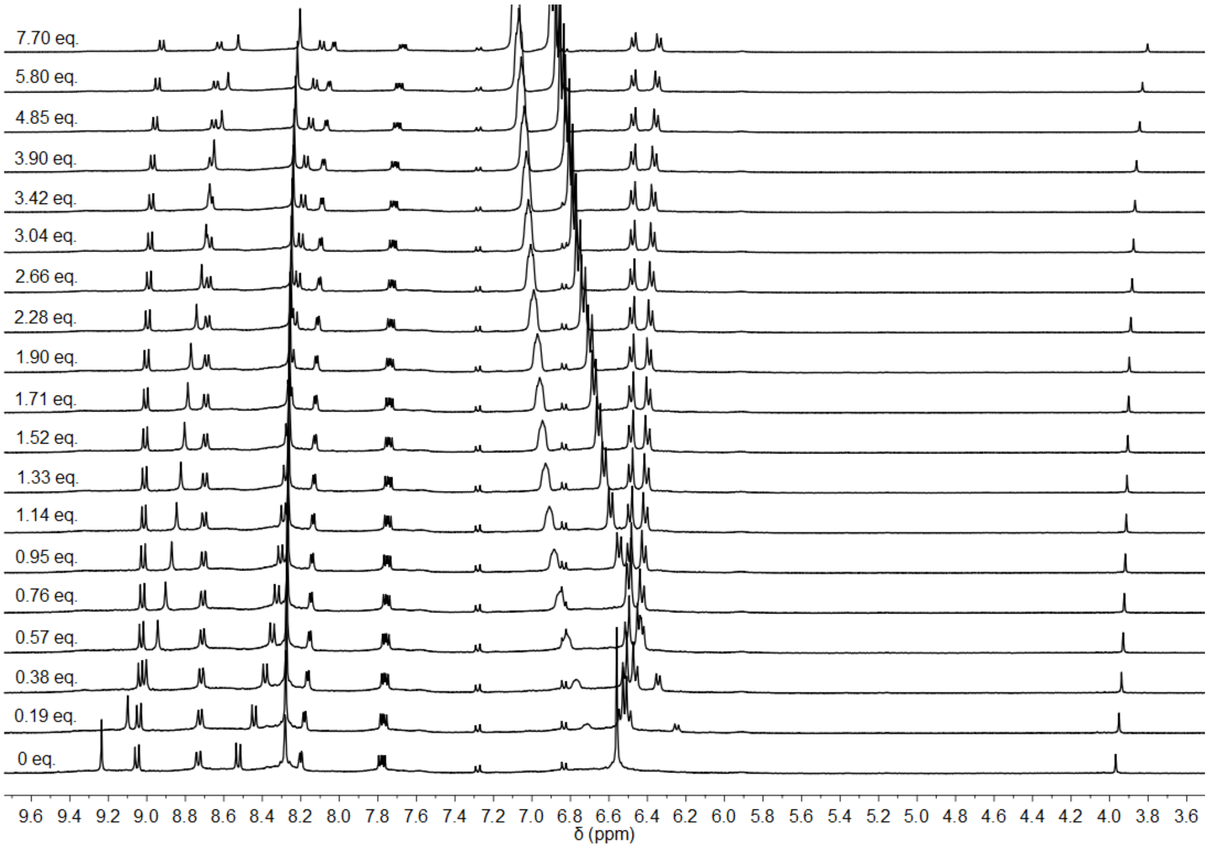

Figure S48. ^{1}H NMR titration (400 MHz, 298 K) of KB[(C_6H_4Cl)$_4$] into a solution of **2** in CD_3CN (equivalents of anion are labeled on individual spectra).

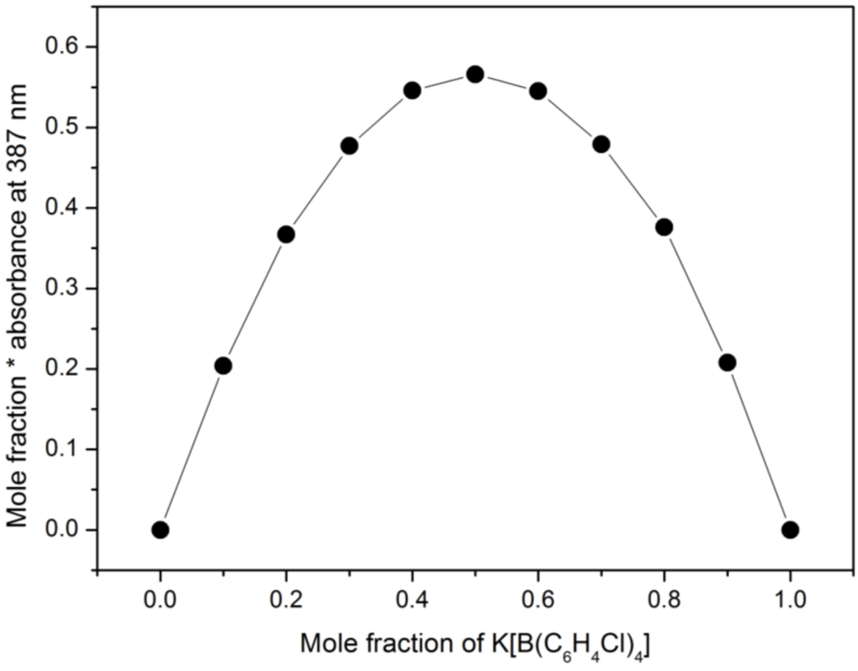

Figure S49. Job plot of K[B(C_6H_4Cl)$_4$] with **2** in CH_3CN to determine the 1:1 binding stoichiometry, measured by UV-Vis spectroscopy.

(a)

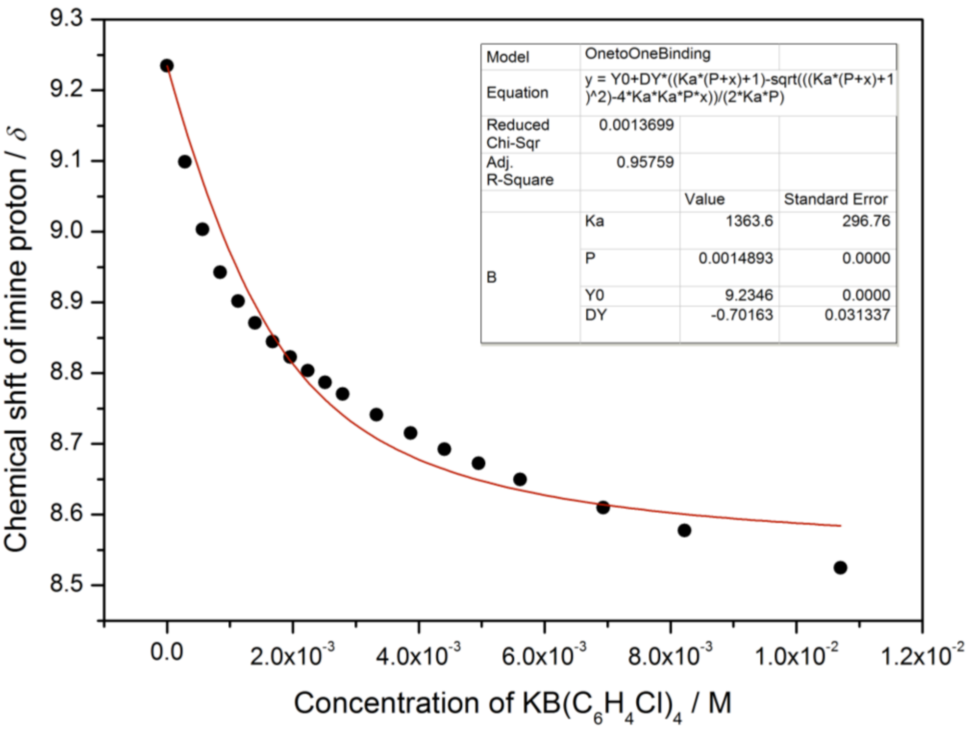

(b)

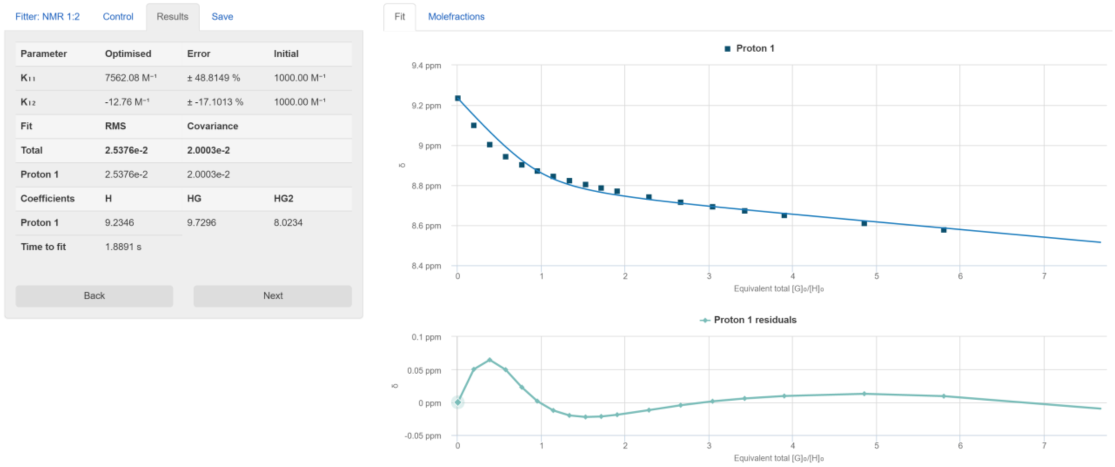

Figure S50. (a) Binding isotherm (1:1 system) fit to the chemical shift of the imine proton *vs.* the concentration of BPh_4^- added to determine binding affinity (K_a). (b) Binding isotherm (1:2 system) calculated using Bindfit.[14] High and sigmoidal residuals, along with large errors and a negative value of K_{12}, indicate that a 1:2 model does not fit the data better than a 1:1 binding isotherm.[15] We hypothesise that, while the binding is approximately 1:1 at low concentrations of guest, the cage may begin to bind more than one guest when a large excess of guest is added (necessary to perform an adequate data fit). The resulting isotherm may be a combination of 1:1 and 1:2 (and potentially 1:3 and 1:4) binding events.

8.4 CI_4 (tetraiodomethane)

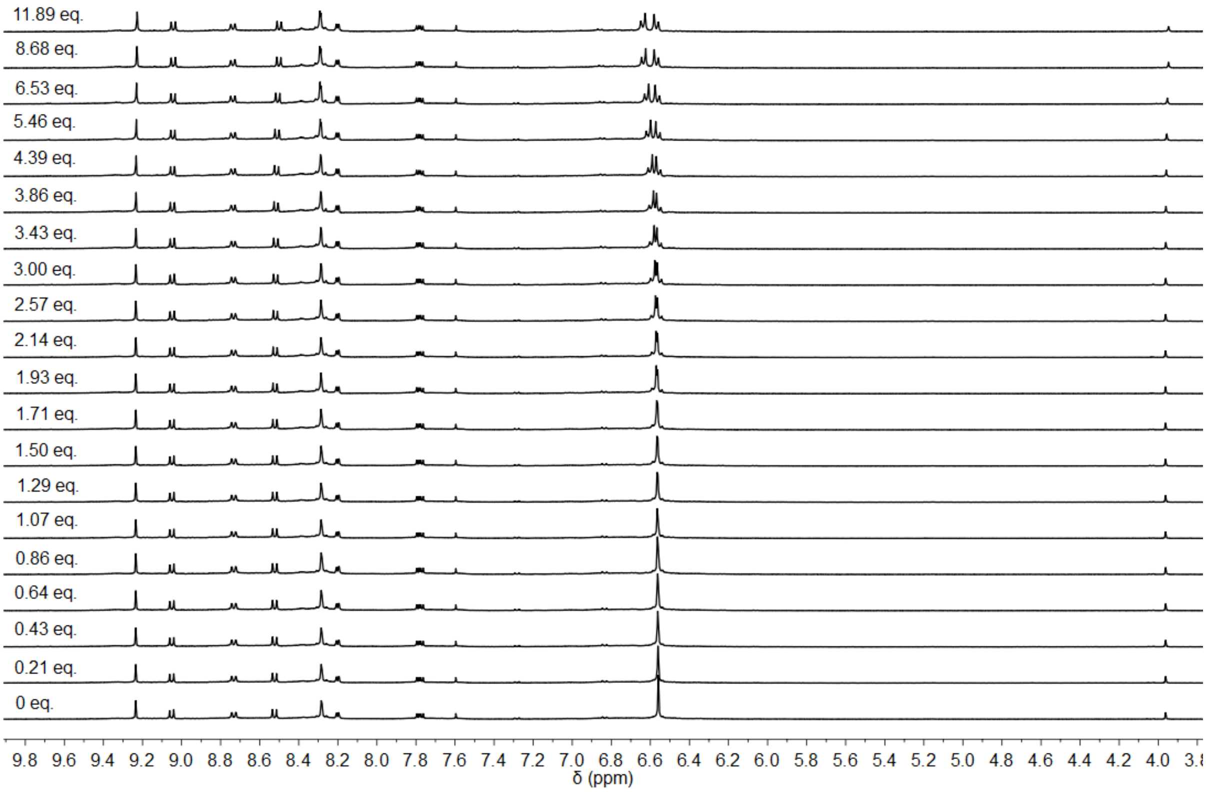

Figure S51. ^{1}H NMR titration (400 MHz, 298 K) of CI_4 into a solution of **2** in CD_3CN (equivalents of the neutral molecule are labeled on individual spectra).

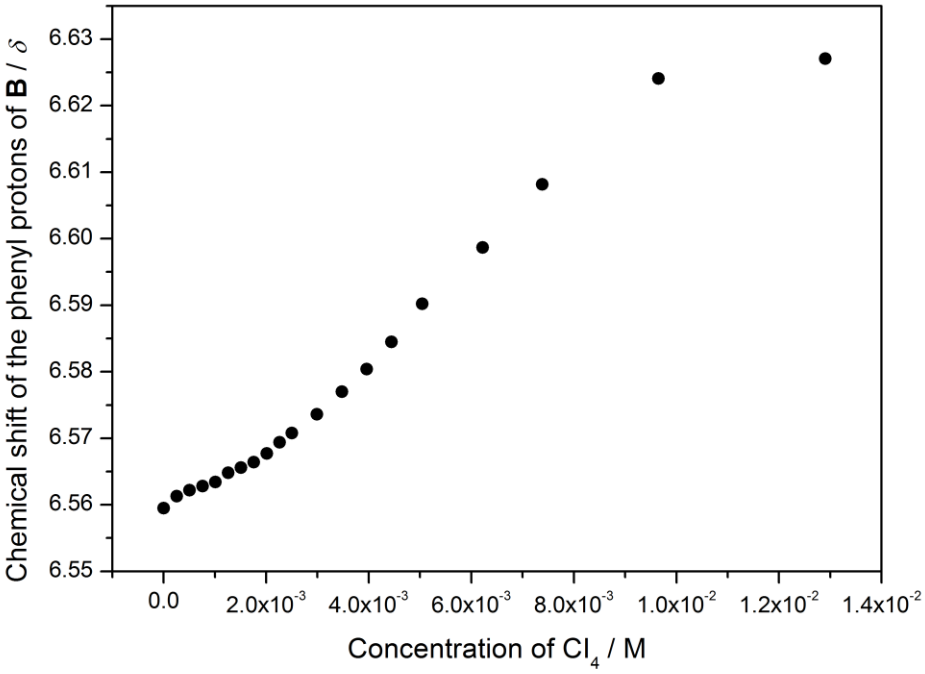

Figure S52. A plot of the chemical shift of the phenyl protons of **B** *vs.* the concentration of CI_4 added. The data could not be fitted to a 1:1 binding model, appearing to correspond to multiple (>2) binding events.

8.5 CBr_4 (tetrabromomethane)

No significant changes in the spectrum of **2** were observed with up to 8 equivalents of CBr_4. The addition of 15 and 25 equivalents of CBr_4 produced the spectral changes noted in Figure S53.

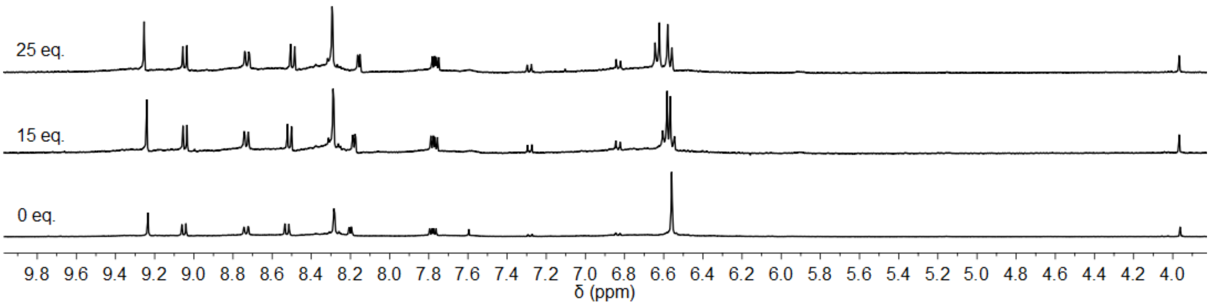

Figure S53. ^{1}H NMR titration (400 MHz, 298 K) of CBr_4 into a solution of **2** in CD_3CN (equivalents of the neutral molecule are labeled on individual spectra).

8.6 CHI_3 (iodoform)

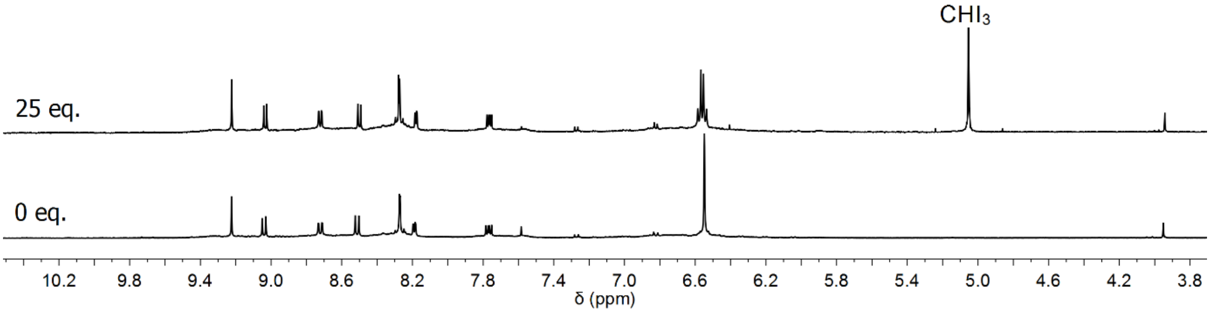

Figure S54. ^{1}H NMR spectra (500 MHz, 298 K, CD_3CN) of free **2** (bottom, 0 eq.) compared to **2** with 25 equivalents of CHI_3 (top).

8.7 Other halogenated guests

No significant changes in the spectrum of **2** were observed upon the addition of up to 50 equivalents of CCl_4, $CHBr_3$, CH_2Cl_2, $CHCl_3$ or CH_2BrI. The addition of 1 mL of CCl_4 led to slight spectral changes, which we attribute to solvent changes, rather than direct encapsulation of the guest.

8.8 $CsCB_{11}H_{12}$

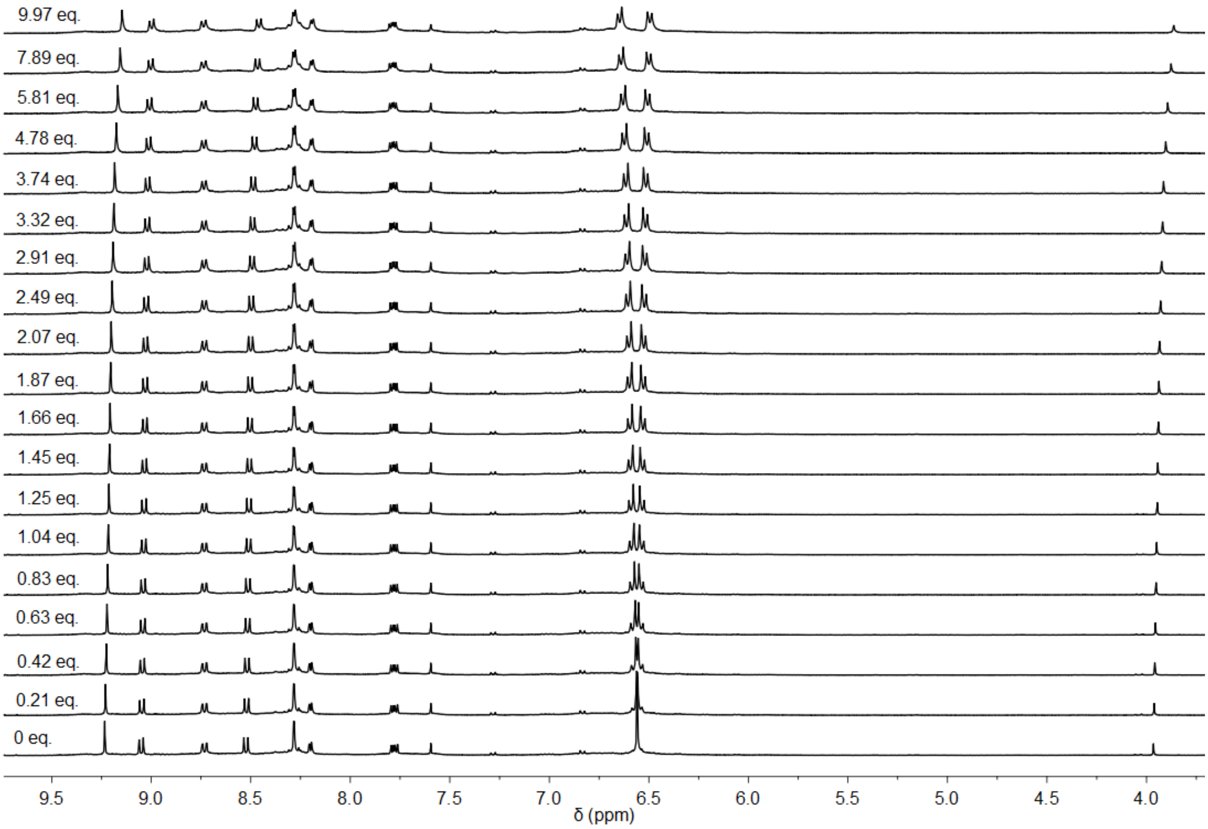

Figure S55. ^{1}H NMR titration (400 MHz, 298 K) of $CsCB_{11}H_{12}$ into a solution of **2** in CD_3CN (equivalents of anion are labeled on individual spectra).

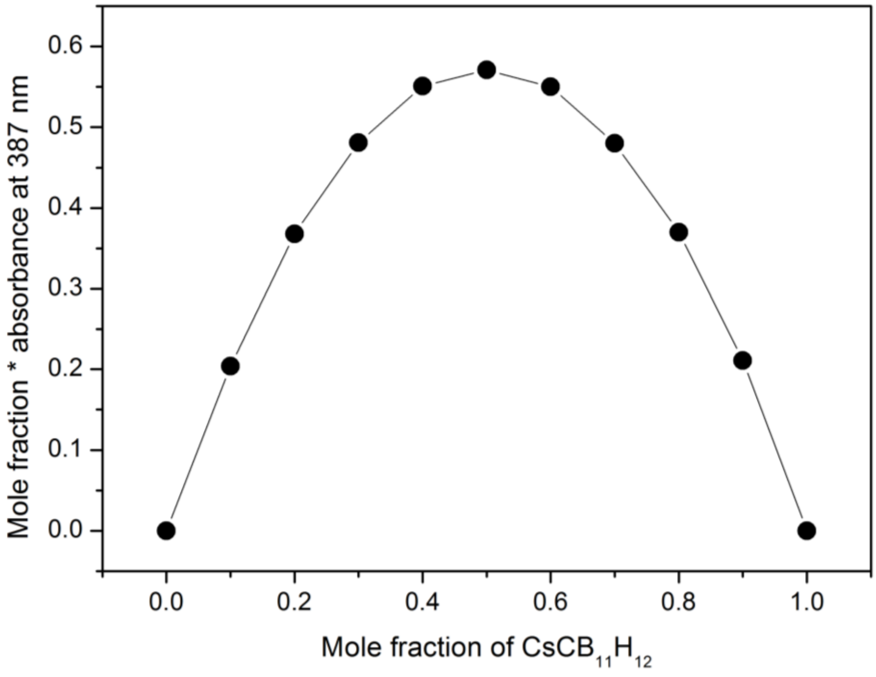

Figure S56. Job plot of $CsCB_{11}H_{12}$ with **2** in CH_3CN to determine the 1:1 binding stoichiometry, measured by UV-Vis spectroscopy.

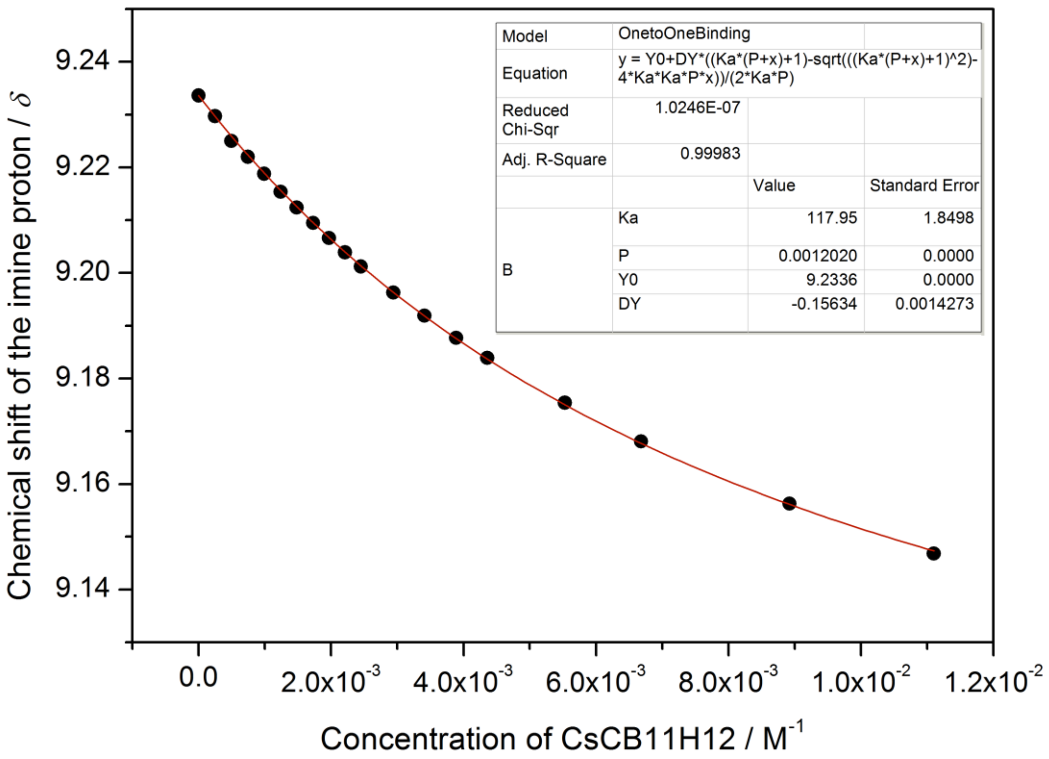

Figure S57. Binding isotherm (1:1 system) fit to the chemical shift of the imine proton *vs.* the concentration of $CsCB_{11}H_{12}$ added to determine binding affinity (K_a).

8.9 $K_2B_{12}F_{12}$

Significant broadening of the signals was observed past the addition of more than 2.05 equivalents of anion. Precipitation was observed past 3.29 equivalents, even at UV-Vis concentrations (10^{-5} M), thus preventing determination of a binding constant.

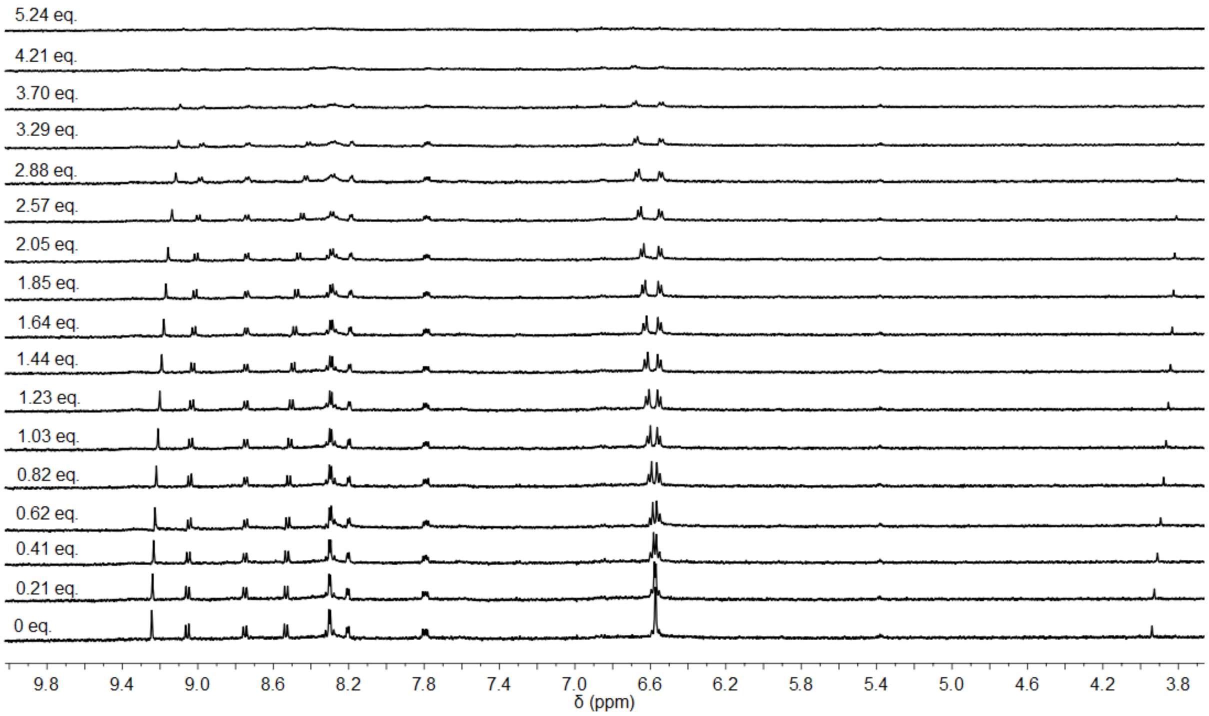

Figure S58. ^{1}H NMR titration (500 MHz, 298 K) of $K_2B_{12}F_{12}$ into a solution of **2** in CD_3CN (equivalents of anion are labeled on individual spectra).

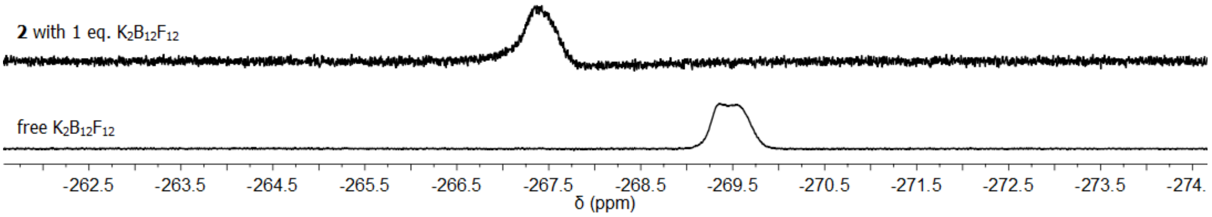

Figure S59. ^{19}F NMR spectra (376 MHz, 298 K, CD_3CN) of free $K_2B_{12}F_{12}$ (bottom) and a solution of **2** containing one equivalent of $K_2B_{12}F_{12}$.

8.10 $Cs_2B_{12}H_{12}$

The guest was insoluble in CH_3CN, preventing determination of a binding constant.

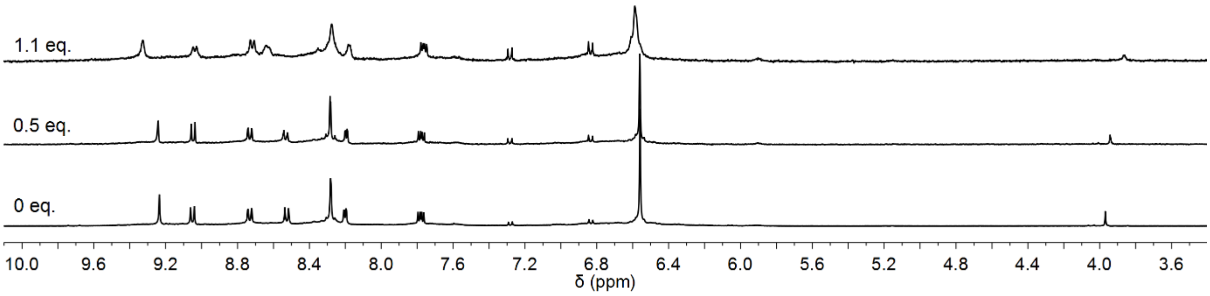

Figure S60. ^{1}H NMR spectra (400 MHz, 298 K) of **2** with 0.5 and 1.1 equivalents of $Cs_2B_{12}H_{12}$, compared to the unoccupied host (0 eq.). Significant precipitation was observed when more than 1.5 equivalents of guest were added.

8.11 ^{n}Bu$_4$NPF$_6$

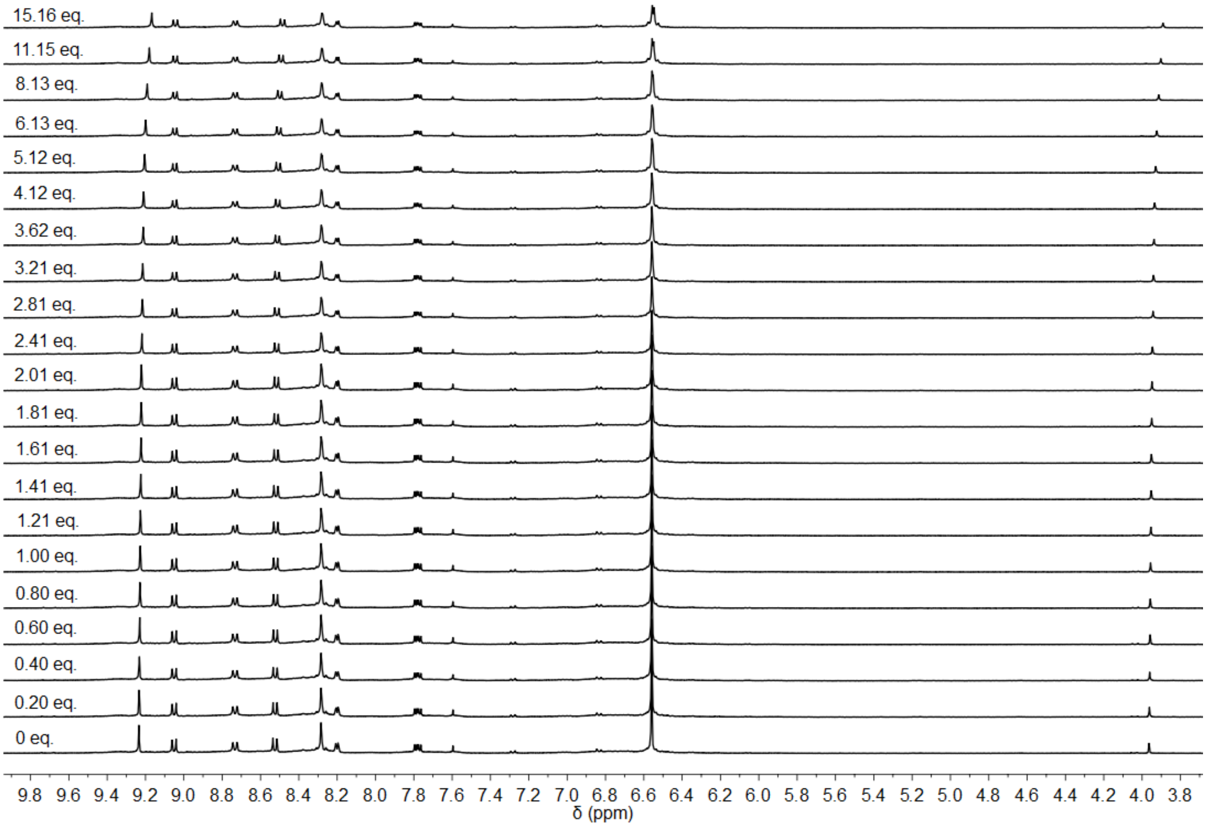

Figure S61. ^{1}H NMR titration (400 MHz, 298 K) of ^{n}Bu$_4$NPF$_6$ into a solution of **2** in CD$_3$CN (equivalents of anion are labeled on individual spectra).

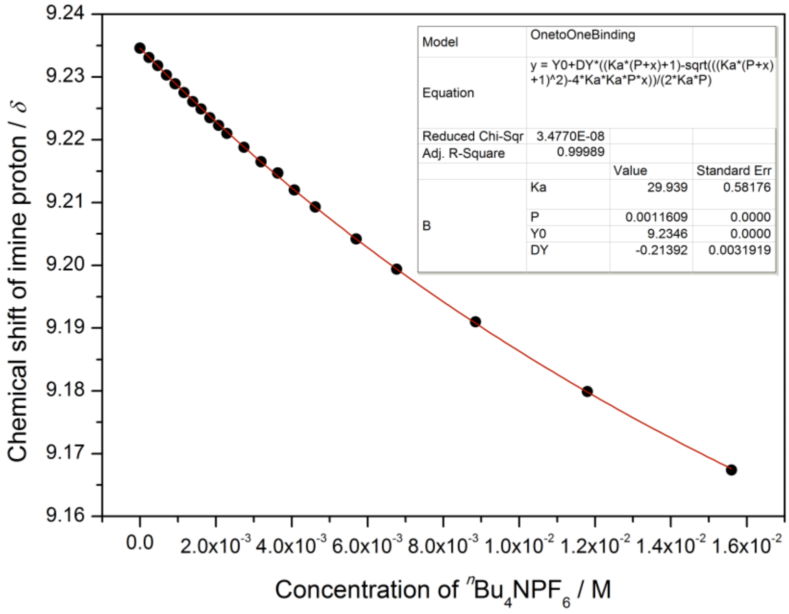

Figure S62. Binding isotherm (1:1 system) fit to the chemical shift of the imine proton *vs.* the concentration of ^{n}Bu$_4$NPF$_6$ added to determine binding affinity (K_a).

8.12 $KAsF_6$

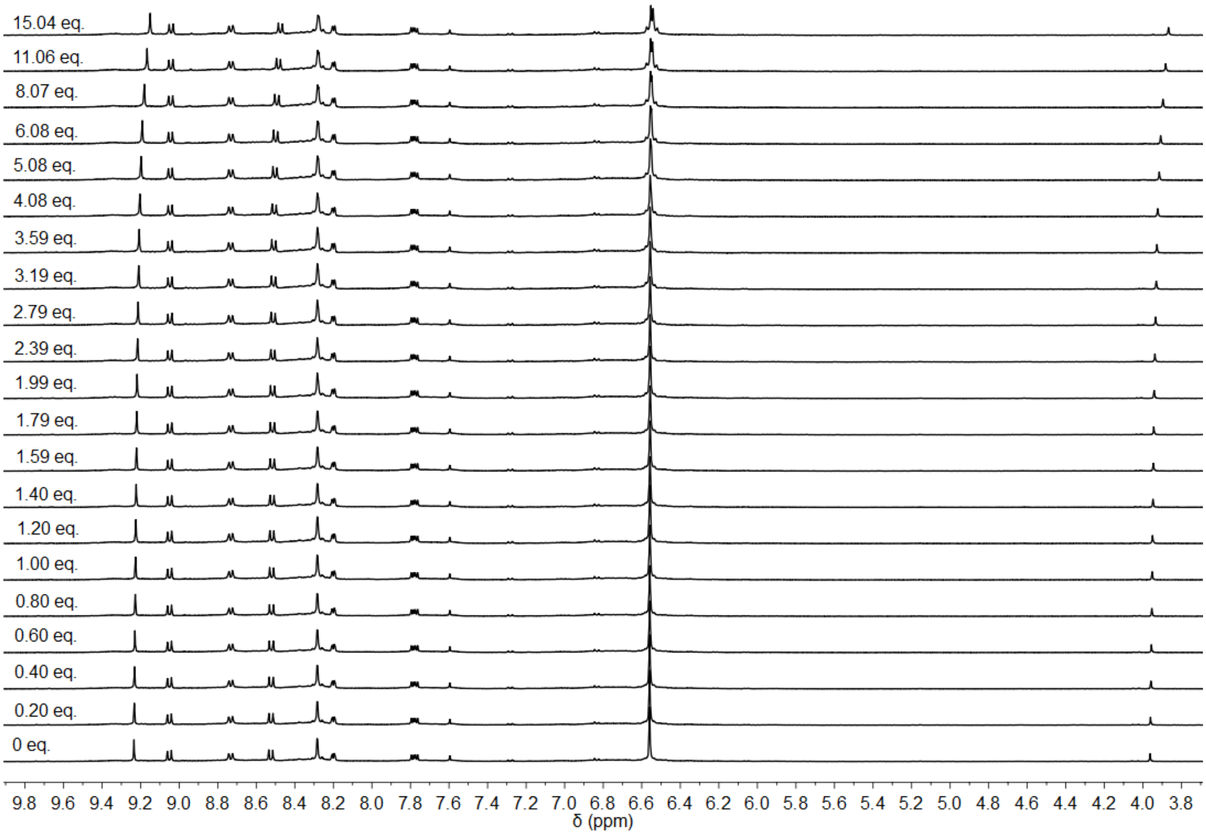

Figure S63. ^1H NMR titration (400 MHz, 298 K) of $KAsF_6$ into a solution of **2** in CD_3CN (equivalents of anion are labeled on individual spectra).

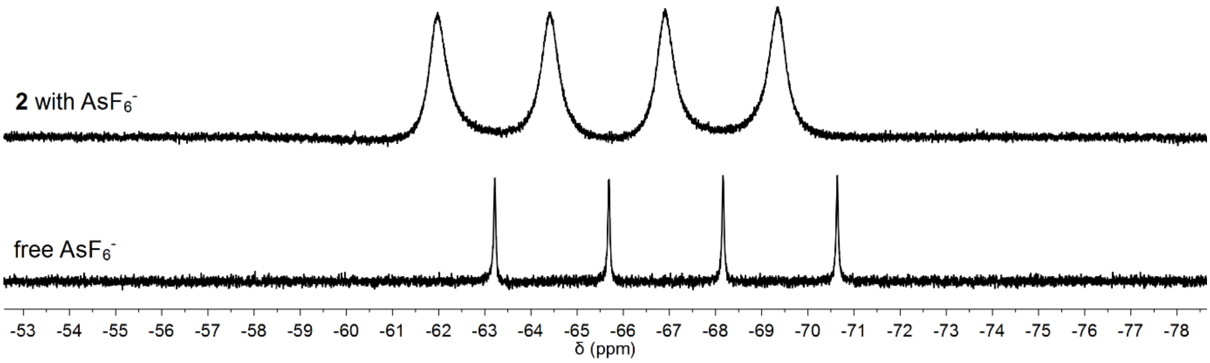

Figure S64: ^{19}F NMR spectra (376 MHz, 298 K, CD_3CN) of free $KAsF_6$ (bottom) and a solution of **2** containing 10 equivalents of $KAsF_6$ (top).

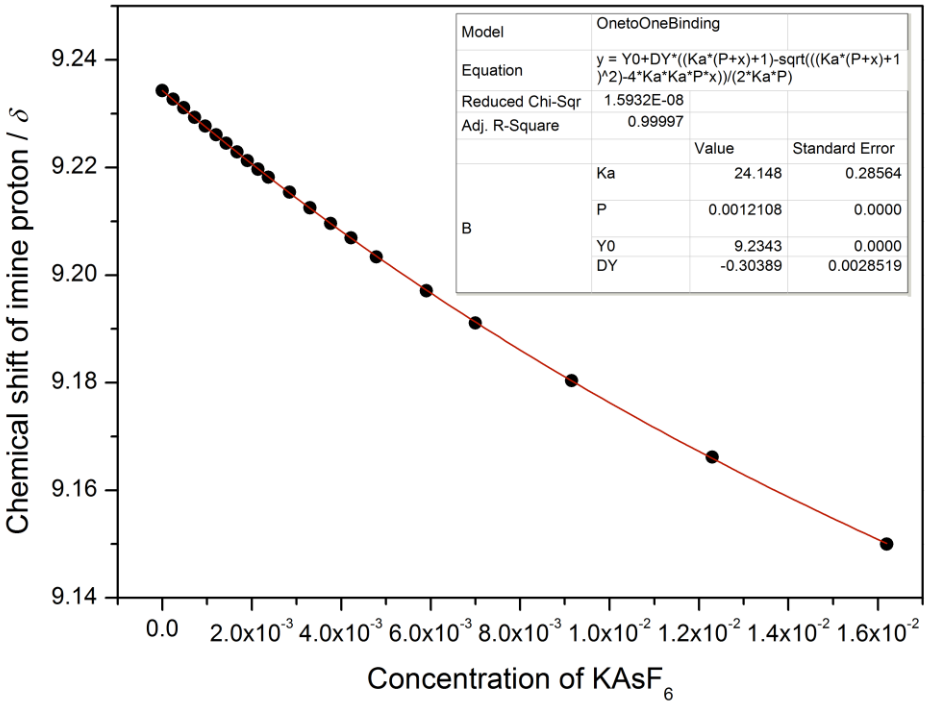

Figure S65. Binding isotherm (1:1 system) fit to the chemical shift of the imine proton vs. the concentration of $KAsF_6$ added to determine binding affinity (K_a).

8.13 $NaSbF_6$

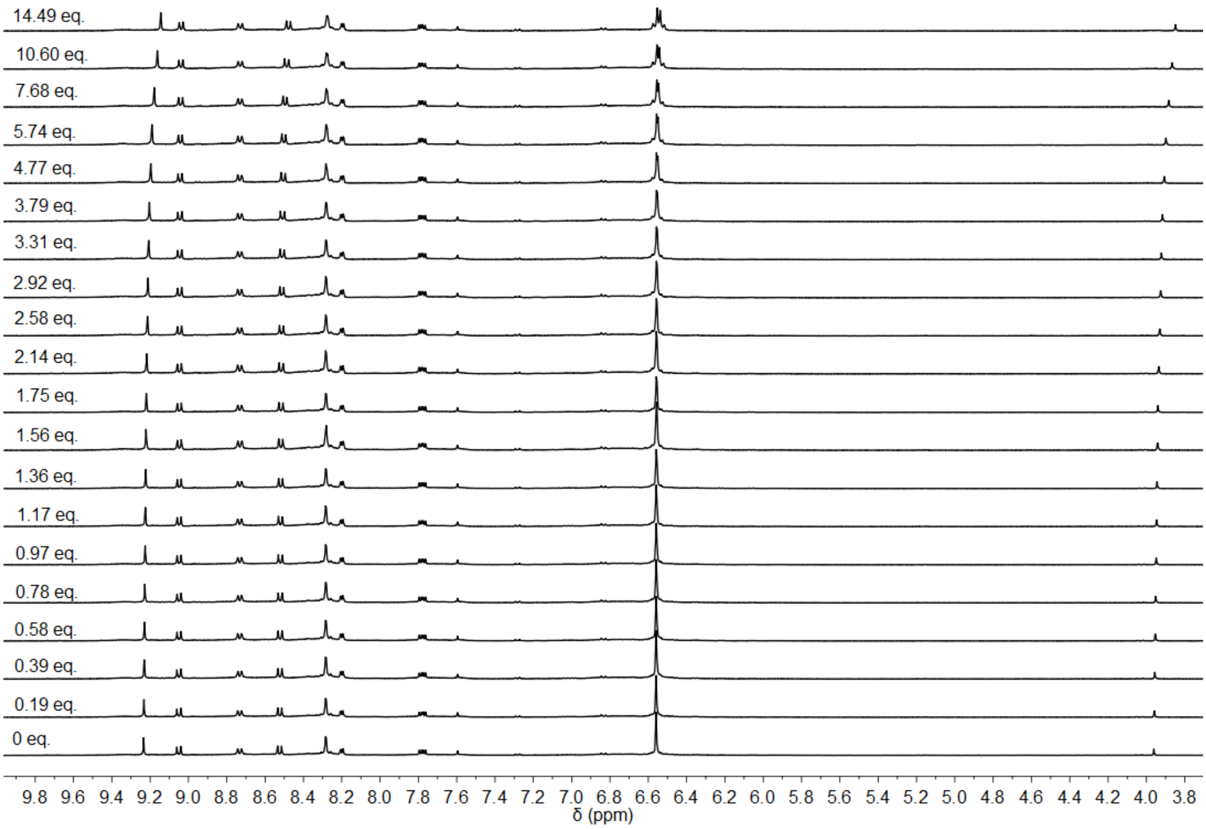

Figure S66. ^{1}H NMR titration (400 MHz, 298 K) of $NaSbF_6$ into a solution of **2** in CD_3CN (equivalents of anion are labeled on individual spectra).

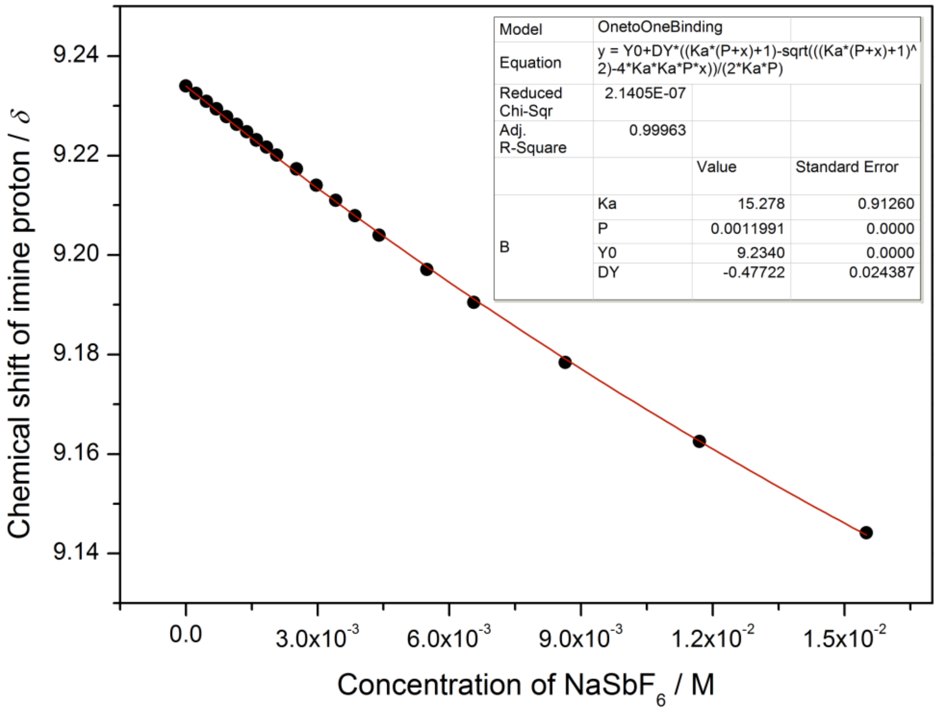

Figure S67. Binding isotherm (1:1 system) fit to the chemical shift of the imine proton *vs.* the concentration of $NaSbF_6$ added to determine binding affinity (K_a).

9. Host-guest chemistry of **3**

9.1 ^{n}Bu$_4$NBPh$_4$

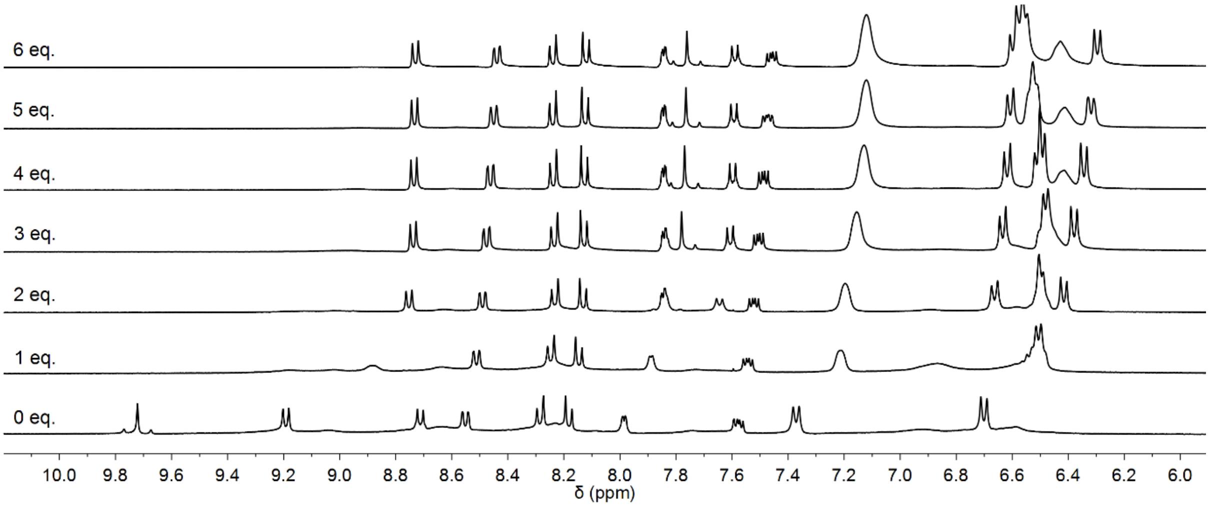

Figure S68. ^{1}H NMR titration (400 MHz, 298 K) of ^{n}Bu$_4$NBPh$_4$ into a solution of **3** in CD_3CN (equivalents of anion are labeled on individual spectra).

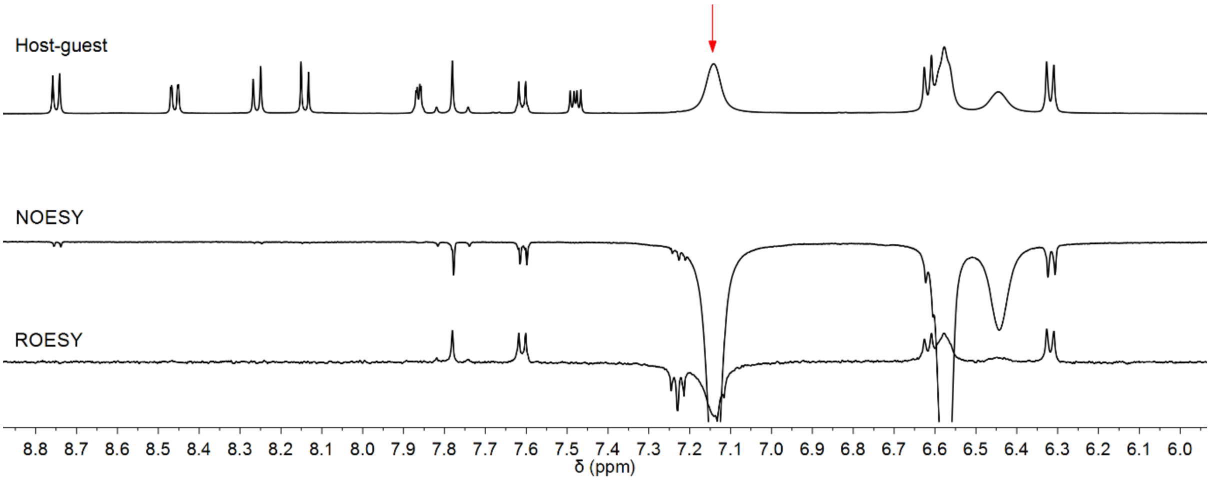

Figure S69. 1D selective ^{1}H NOESY and ROESY spectra (500 MHz, 298 K, CD_3CN) irradiating the *ortho* proton (red arrow) of BPh_4^- in BPh_4^-•**3**.

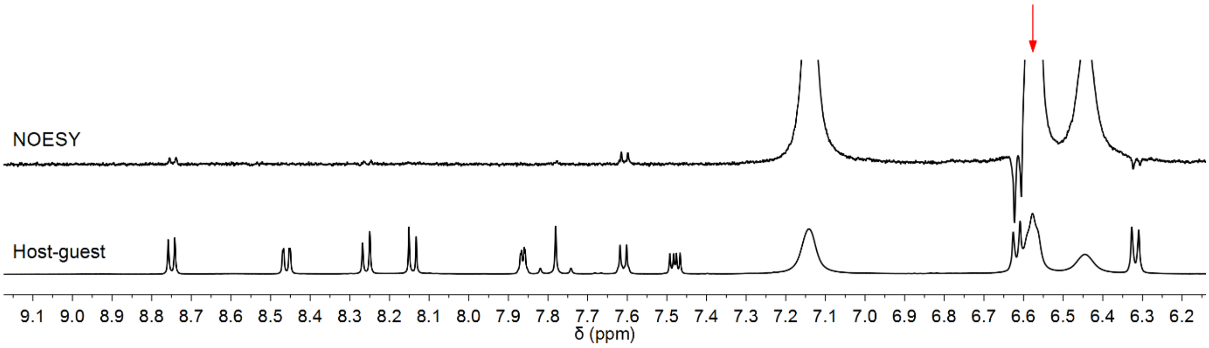

Figure S70. 1D selective ^{1}H NOESY spectrum (500 MHz, 298 K, CD_3CN) irradiating the *meta* proton (red arrow) of BPh_4^- in BPh_4^-•**3**.

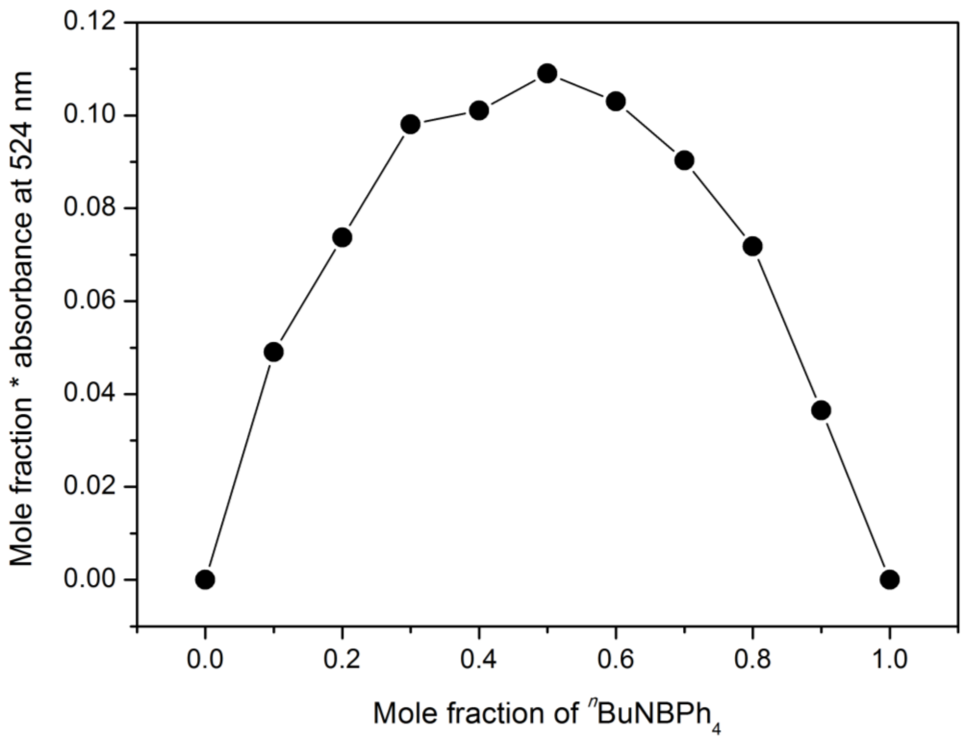

Figure S71. Job plot of ^{n}BuNBPh$_4$ with **3** in CH$_3$CN to determine the 1:1 binding stoichiometry, measured by UV-Vis spectroscopy.

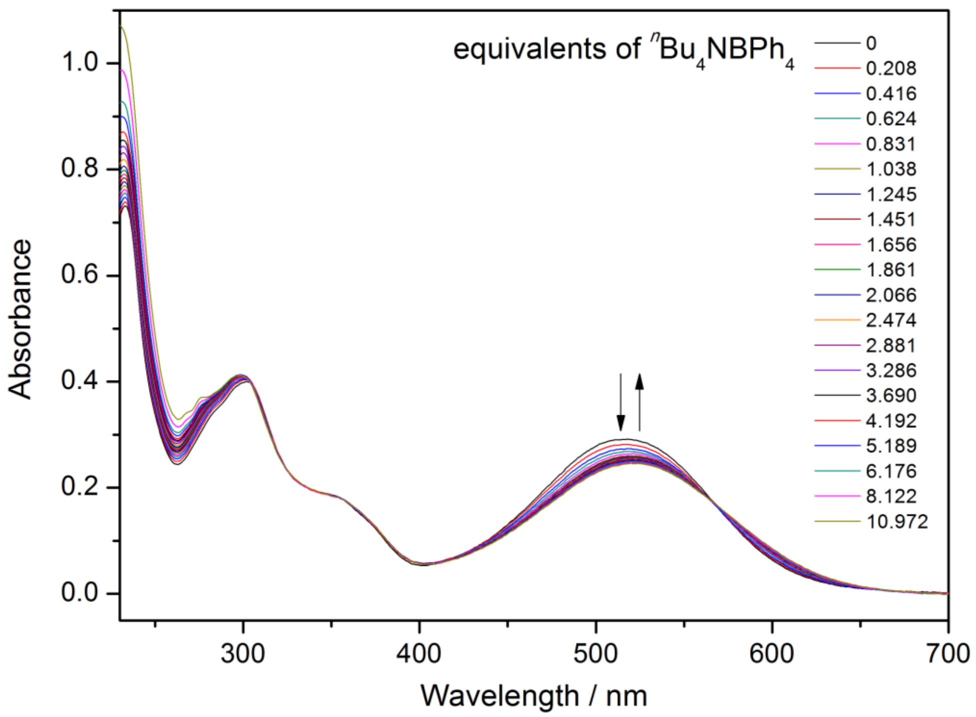

Figure S72. UV-Vis titration of ^{n}Bu$_4$NBPh$_4$ into a solution of **3** in CH$_3$CN at 298 K.

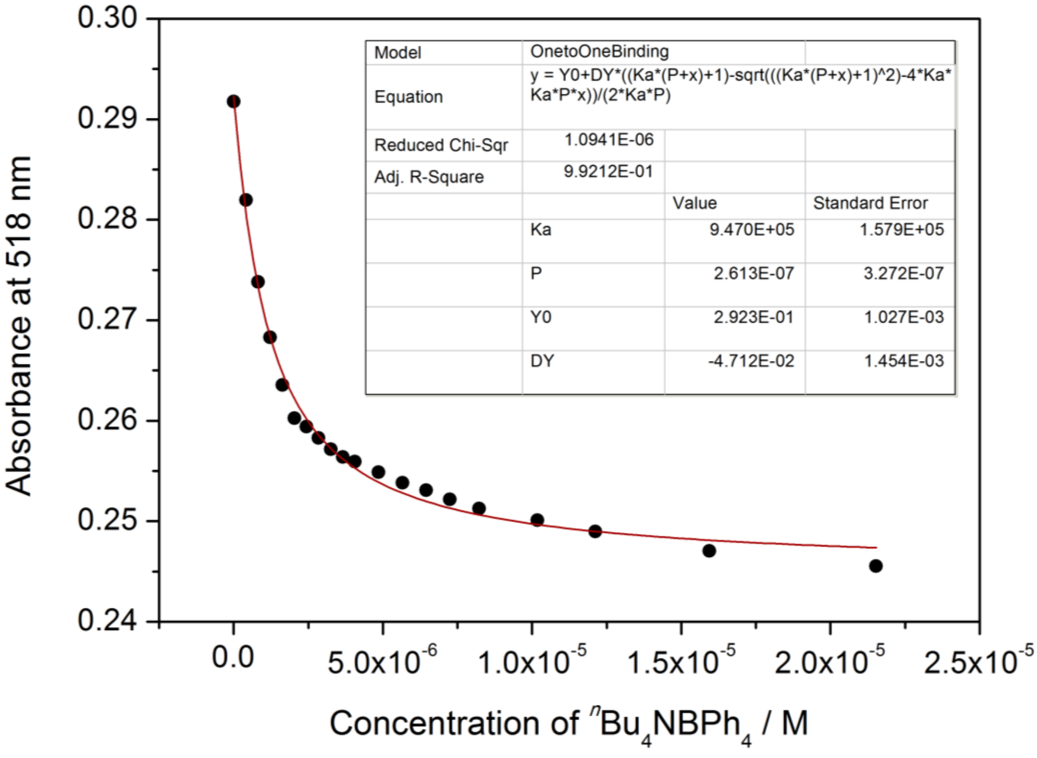

Figure S73. Binding isotherm (1:1 system) fit to the absorbance of **3** at 518 nm *vs.* the concentration of $^{n}Bu_4NBPh_4$ added to determine binding affinity (K_a).

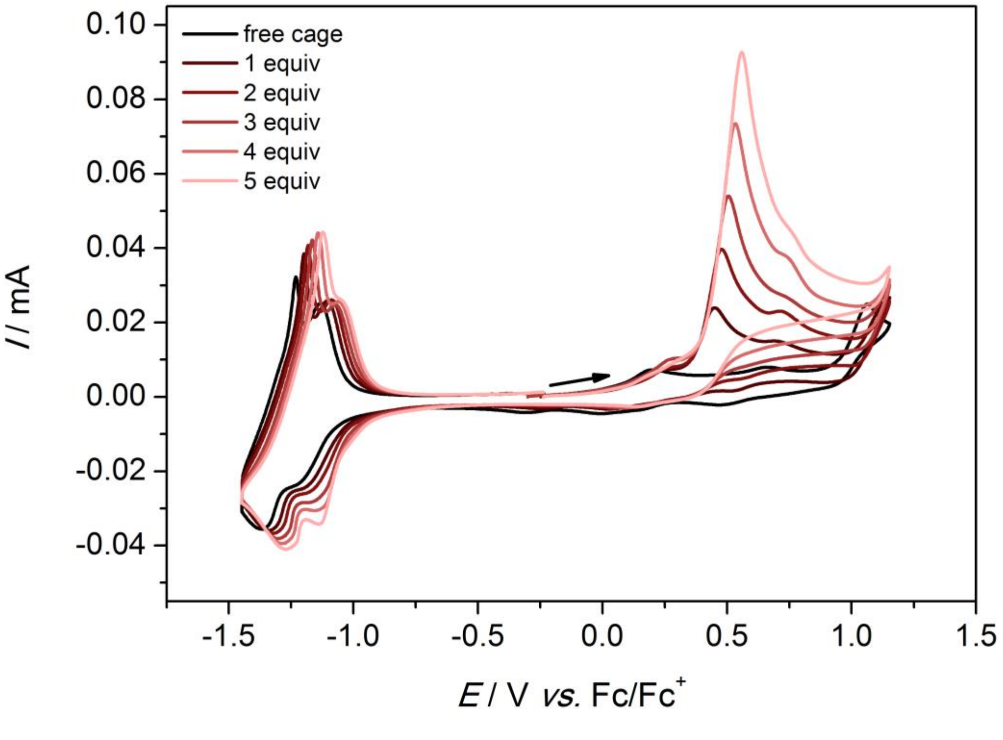

Figure S74. CV of **3** in 0.1 M $^{n}Bu_4NPF_6/CH_3CN$ electrolyte (scan rate = 100 mV s^{-1}), with increasing equivalents of $^{n}Bu_4NBPh_4$. The arrow indicates the direction of scan.

9.2 Na[B(C_6H_4F)$_4$]

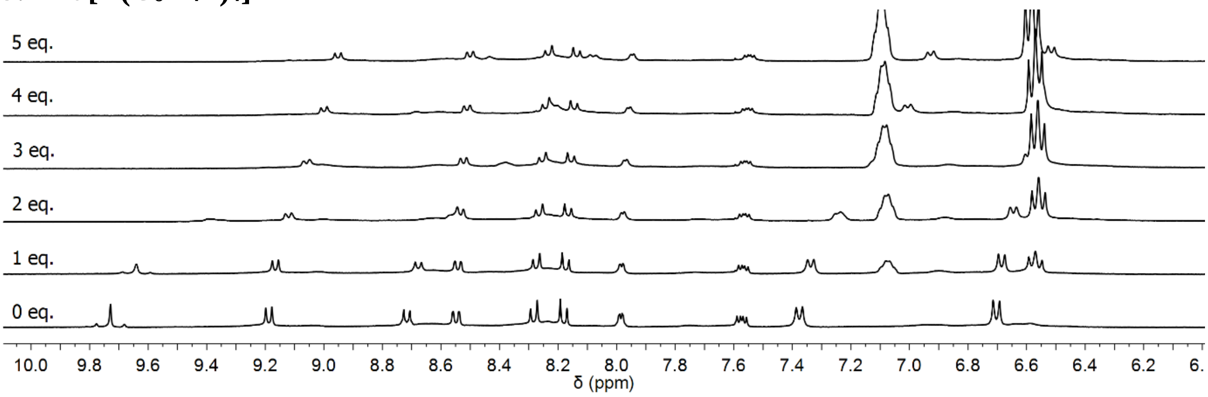

Figure S75. ^{1}H NMR titration (400 MHz, 298 K) of Na[B(C_6H_4F)$_4$] into a solution of **3** in CD_3CN (equivalents of anion are labeled on individual spectra).

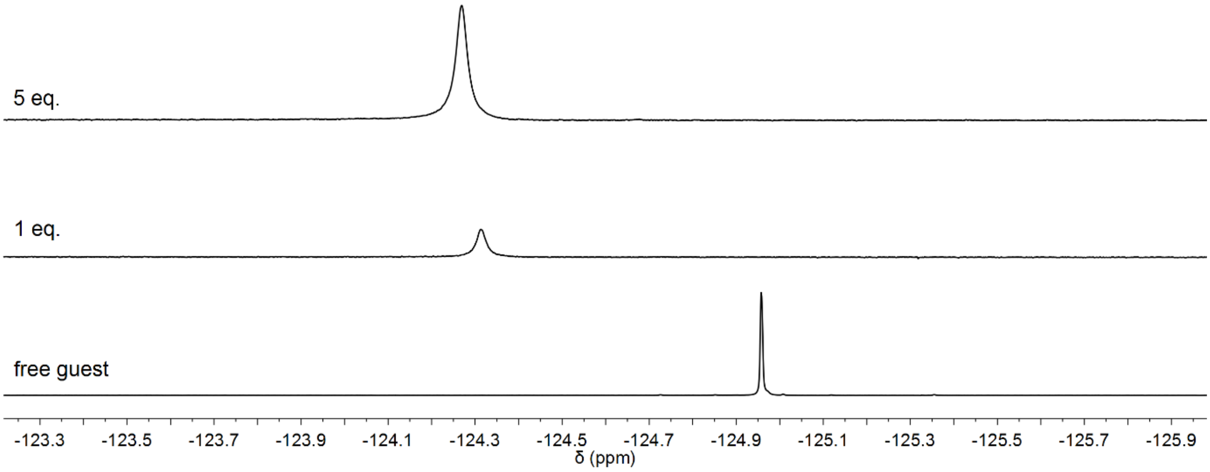

Figure S76. ^{19}F NMR spectra (376 MHz, 298 K, CD_3CN) of **3** with 1 and 5 equivalents of Na[B(C_6H_4F)$_4$], compared to the ^{19}F spectrum of the free B(C_6H_4F)$_4^-$ anion.

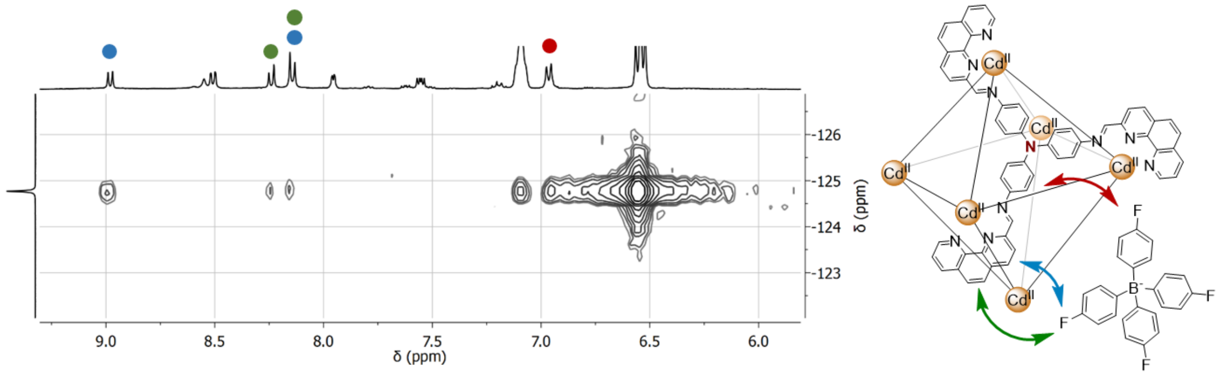

Figure S77. ^{1}H-^{19}F HOESY spectrum (400 MHz, 298 K, CD_3CN) of B(C_6H_4F)$_4^-$•**3**. The interactions corresponding to the HOEs are colour coded to the diagram on the right, showing an interaction between the *p*-F portion of the guest and multiple positions on the phenanthroline ring and the phenyl rings of the host.

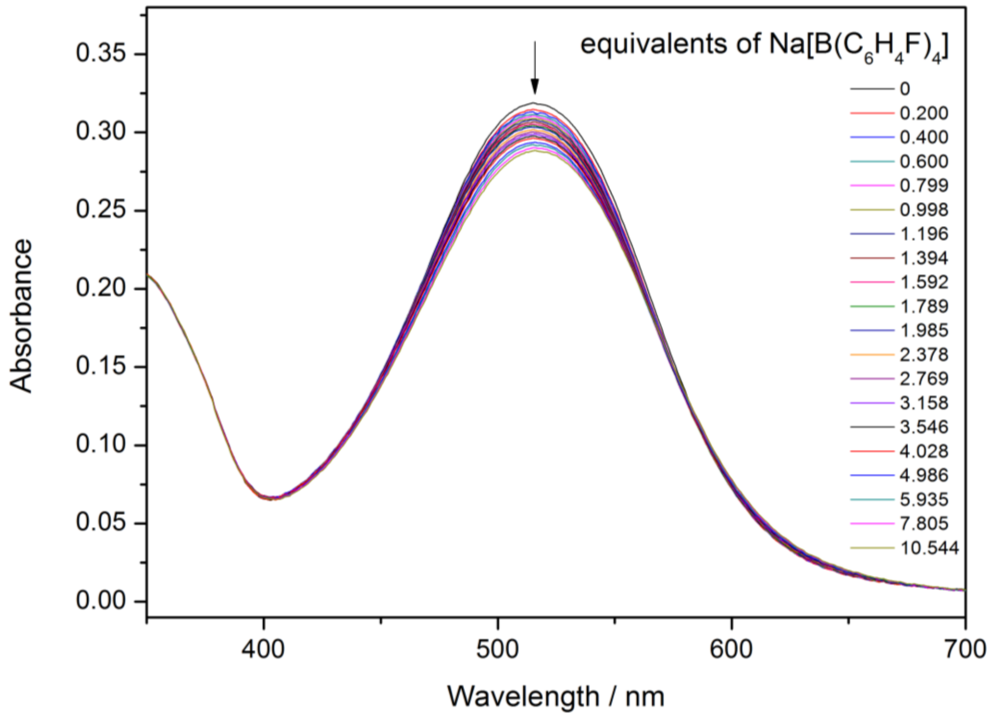

Figure S78. UV-Vis titration of $Na[B(C_6H_4F)_4]$ into a solution of **3** in CH_3CN at 298 K.

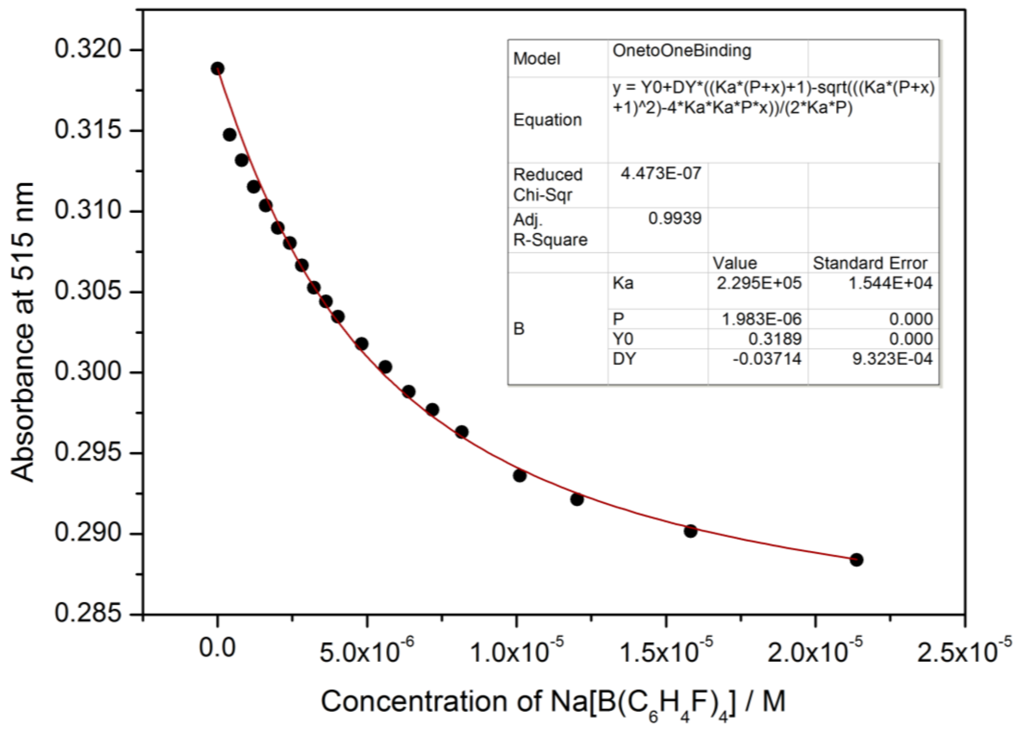

Figure S79. Binding isotherm (1:1 system) fit to the absorbance of **3** at 515 nm *vs.* the concentration of $NaB(C_6H_4F)_4$ added to determine binding affinity (K_a).

9.3 $K[B(C_6H_4Cl)_4]$

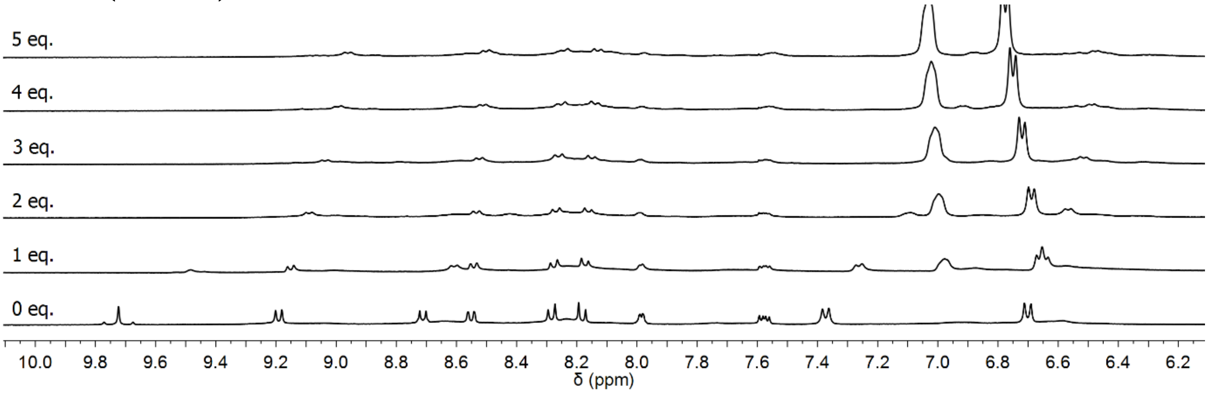

Figure S80. ^{1}H NMR titration (400 MHz, 298 K) of $NaB(C_6H_4F)_4$ into a solution of **3** in CD_3CN (equivalents of anion are labeled on individual spectra).

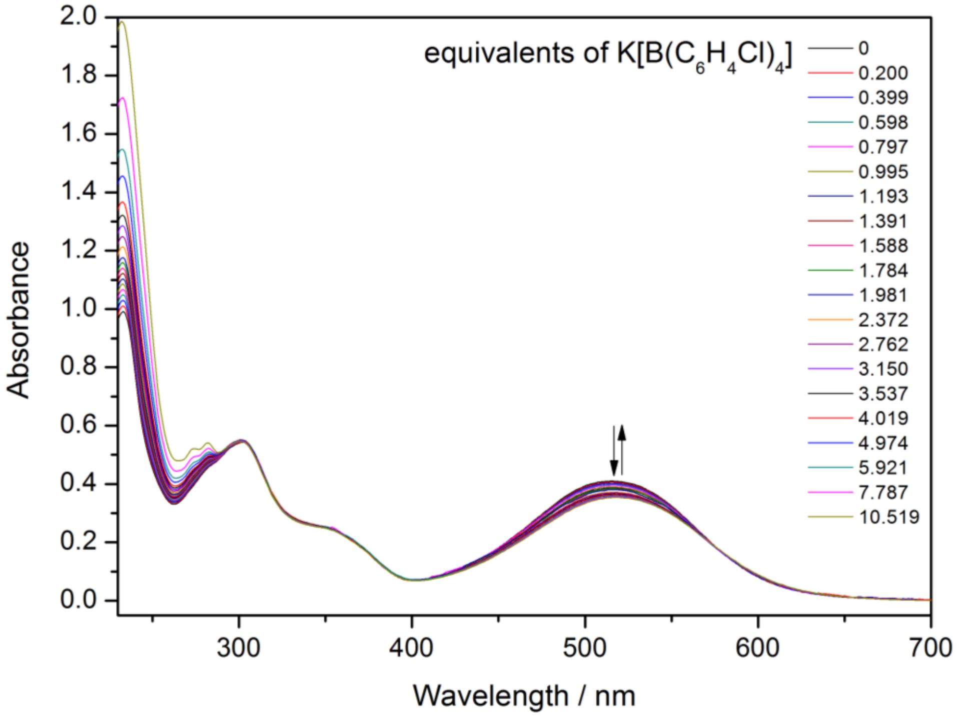

Figure S81. UV-Vis titration of $KB(C_6H_4Cl)_4$ into a solution of **3** in CH_3CN at 298 K.

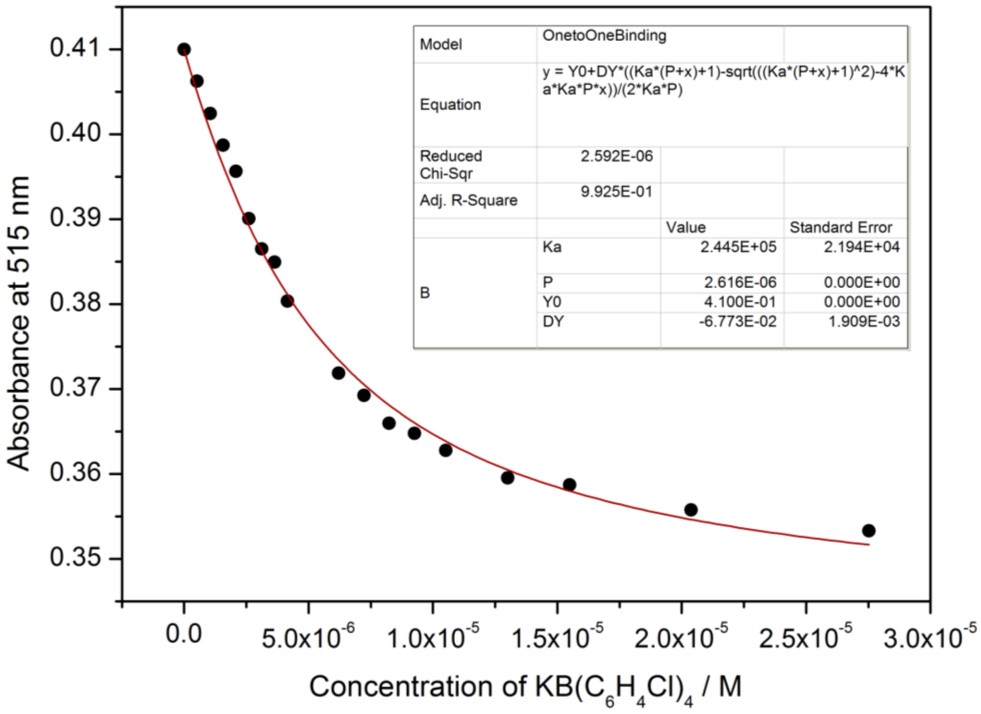

Figure S82. Binding isotherm (1:1 system) fit to the absorbance of **3** at 515 nm *vs.* the concentration of $KB(C_6H_4Cl)_4$ added to determine binding affinity (K_a).

10. Dual binding experiments on **2**

General procedure for $CsCB_{11}H_{12}$: To a 0.6 mL solution of **2** (1.18×10^{-3} M) in CD_3CN was added either 5 or 20 µL (corresponding to 1 or 4 equivalents per cage) of a concentrated solution of either $^{n}Bu_4NBPh_4$ (1.42×10^{-1} M) or $CsCB_{11}H_{12}$ (1.46×10^{-1} M). The resulting solution was then titrated against the other guest, as per the usual procedure.

General procedure for $K_2B_{12}F_{12}$: To a solution of **2** (4.0 mg) in CD_3CN (0.6 mL) was added approximately one equivalent of either $^{n}Bu_4NBPh_4$ (0.41 mg) or $K_2B_{12}F_{12}$ (0.32 mg) and the NMR spectra recorded. The other guest was then added to the solution, and the NMR spectra recorded again. (Note: no titration could be conducted due to the insolubility of the host with more than 2 equivalents of $B_{12}F_{12}^{2-}$).

10.1 $CsCB_{11}H_{12}$ into BPh_4^{-}•2 containing 1 equiv. of BPh_4^{-}

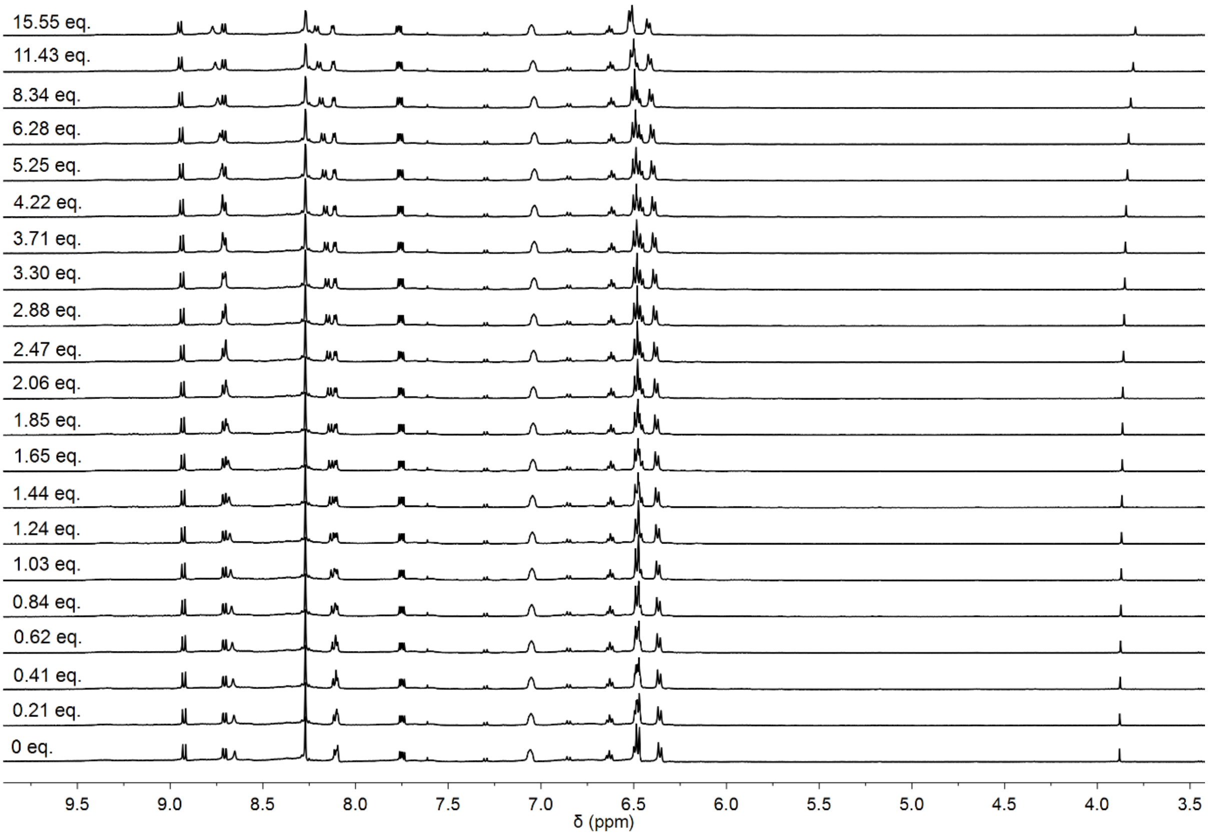

Figure S83. ^{1}H NMR titration (500 MHz, 298 K) of $CsCB_{11}H_{12}$ into a solution of BPh_4^{-}•**2** containing 1 equiv. of BPh_4^{-} in CD_3CN (equivalents of anion are labeled on individual spectra). Only slight downfield shifts of the *ortho* proton of BPh_4^{-} ($\Delta\delta$ = –0.02 ppm) were observed, indicating that BPh_4^{-} remains bound to **2**.

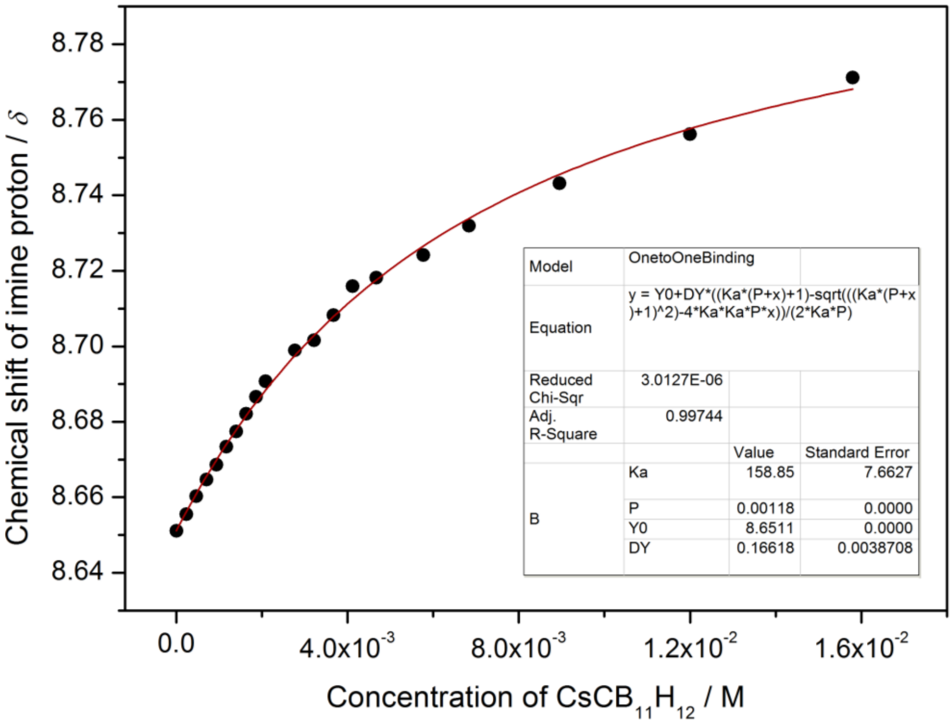

Figure S84. Binding isotherm (1:1 system) fit to the chemical shift of the imine proton *vs.* the concentration of $CsC_{11}H_{12}$ added to determine binding affinity (K_a).

10.2 $CsCB_{11}H_{12}$ into BPh_4^-•2 containing 4 equiv. of BPh_4^-

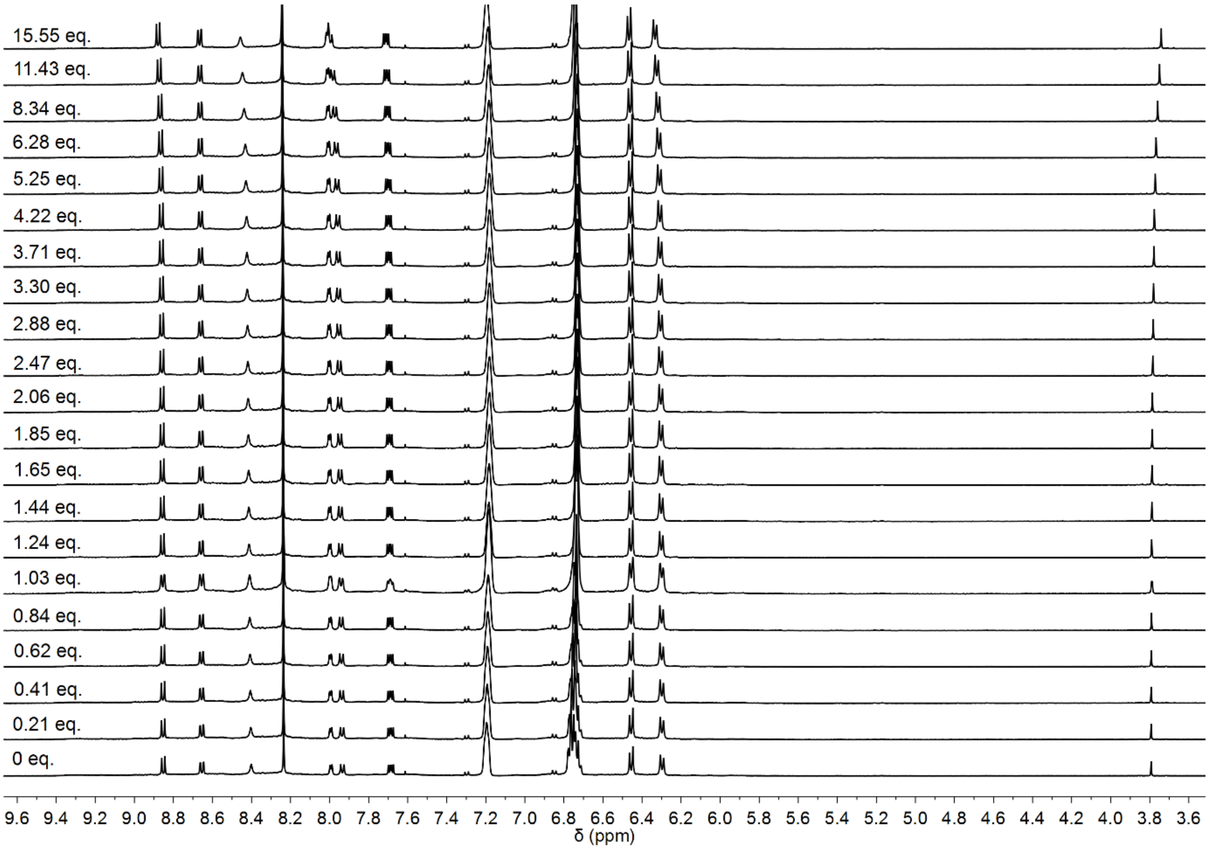

Figure S85. ^{1}H NMR titration (500 MHz, 298 K) of $CsCB_{11}H_{12}$ into a solution of BPh_4^-•**2** containing 4 equiv. of BPh_4^- in CD_3CN (equivalents of anion are labeled on individual spectra). Only slight downfield shifts of the *ortho* proton of BPh_4^- ($\Delta\delta$ = –0.01 ppm) were observed, indicating that BPh_4^- remains bound to **2**.

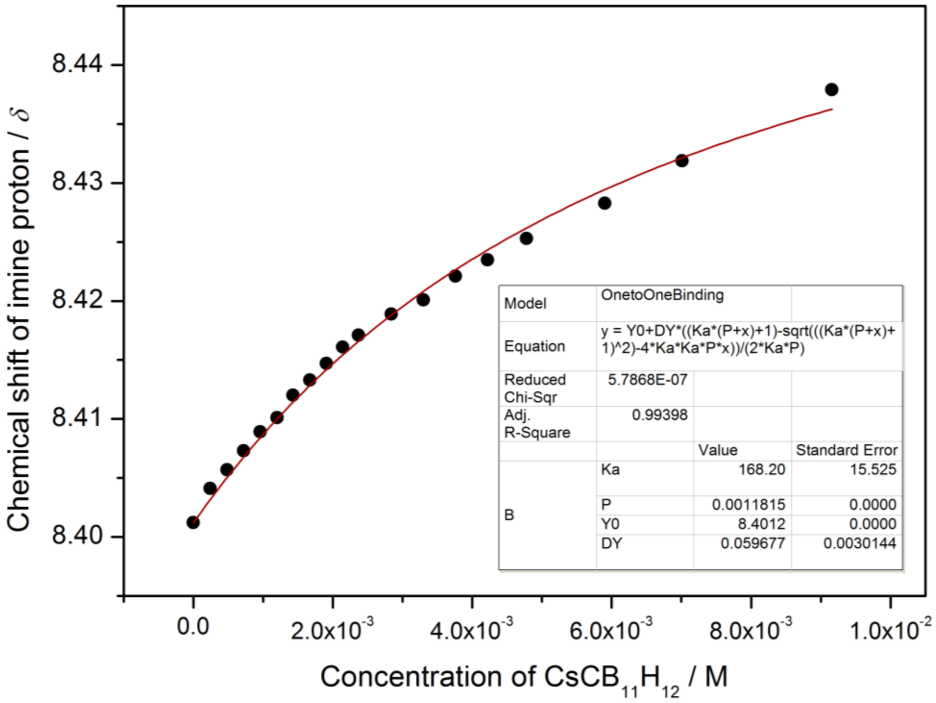

Figure S86. Binding isotherm (1:1 system) fit to the chemical shift of the imine proton *vs*. the concentration of $CsCB_{11}H_{12}$ added to determine binding affinity (K_a).

10.3 $^{n}Bu_4NBPh_4$ into $CB_{11}H_{12}^{-}\subset$**2** containing 1 equiv. of $CB_{11}H_{12}^{-}$

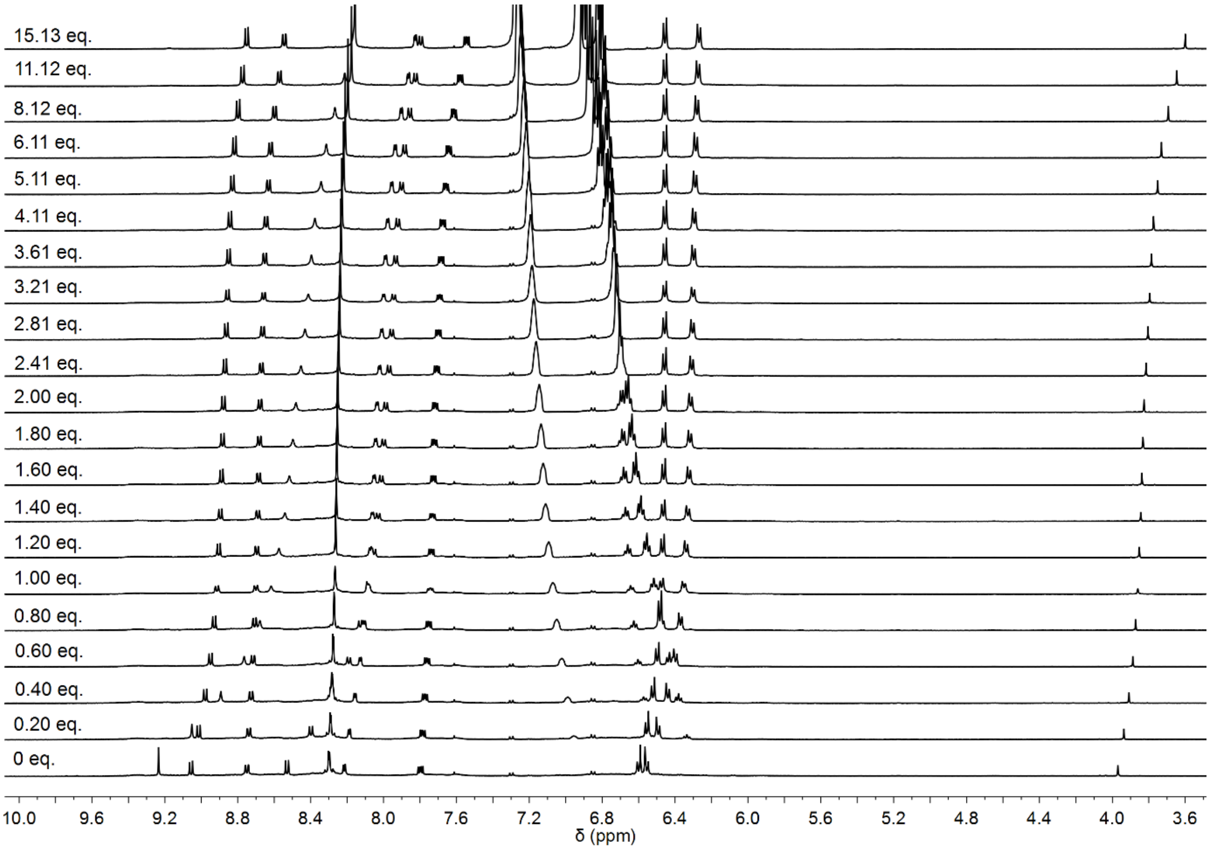

Figure S87. ^{1}H NMR titration (500 MHz, 298 K) of $^{n}Bu_4NBPh_4$ into a solution of $CB_{11}H_{12}^{-}\subset$**2** containing 1 equiv. of $CB_{11}H_{12}^{-}$ in CD_3CN (equivalents of anion are labeled on individual spectra).

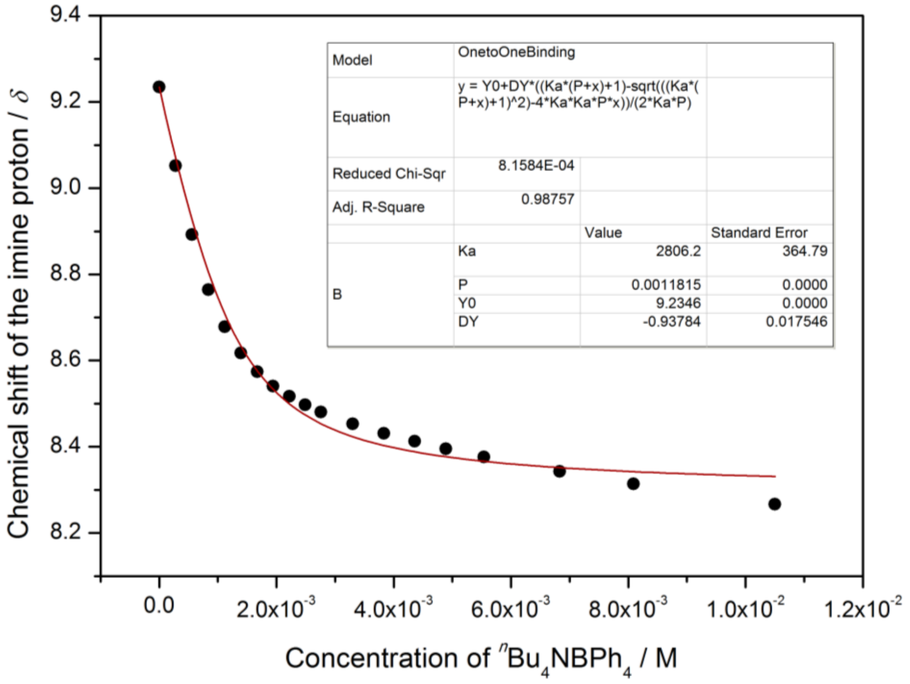

Figure S88. Binding isotherm (1:1 system) fit to the chemical shift of the imine proton *vs.* the concentration of $^{n}Bu_4NBPh_4$ added to determine binding affinity (K_a).

10.4 ${}^{n}Bu_4NBPh_4$ into $CB_{11}H_{12}^{-}\subset$2 containing 4 equiv. of $CB_{11}H_{12}^{-}$

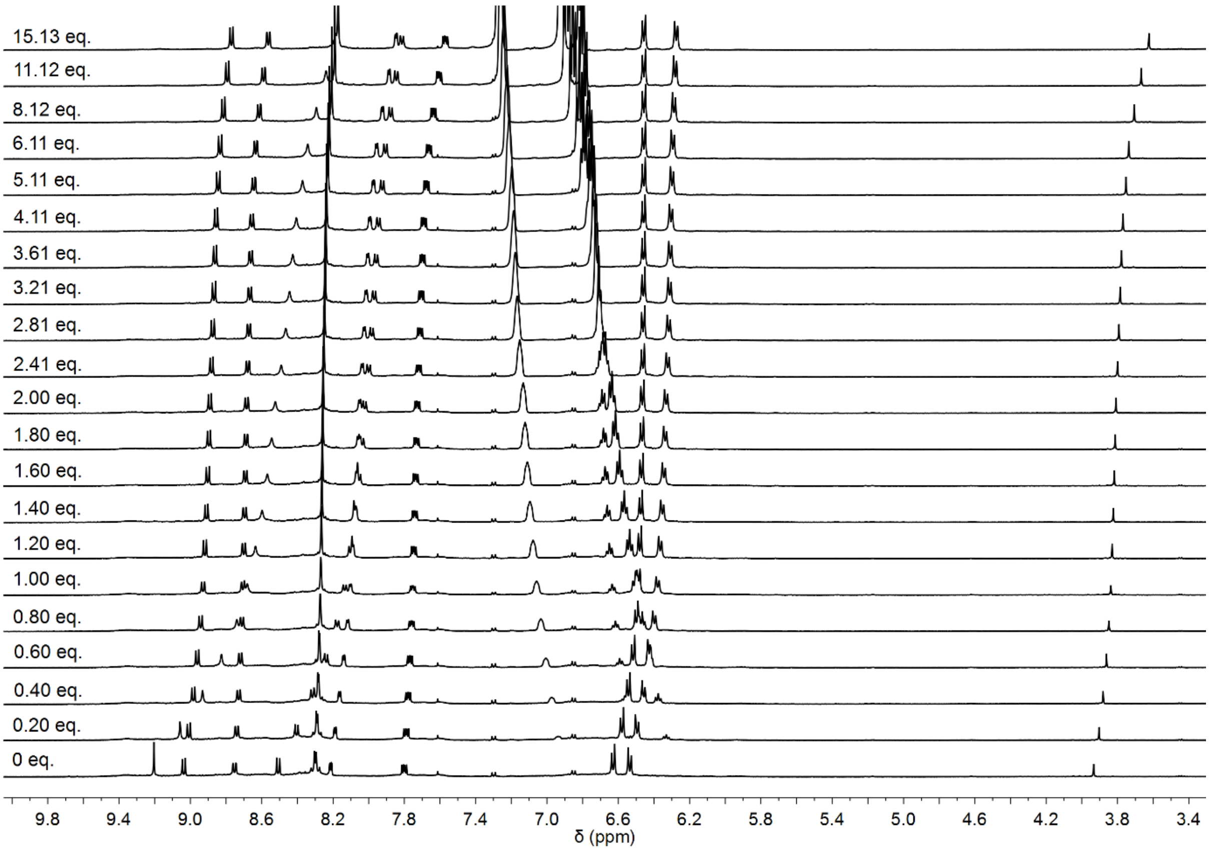

Figure S89. ^{1}H NMR titration (500 MHz, 298 K) of ${}^{n}Bu_4NBPh_4$ into a solution of $CB_{11}H_{12}^{-}\subset$**2** containing 4 equiv. of $CB_{11}H_{12}^{-}$ in CD_3CN (equivalents of anion are labeled on individual spectra).

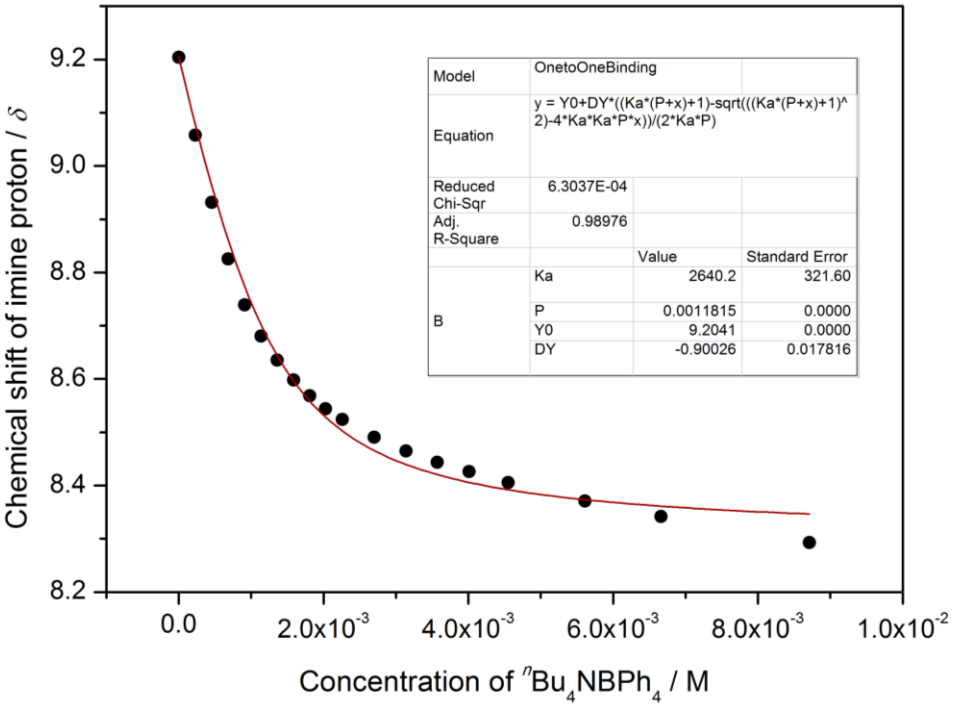

Figure S90. Binding isotherm (1:1 system) fit to the chemical shift of the imine proton *vs*. the concentration of ${}^{n}Bu_4NBPh_4$ added to determine binding affinity (K_a).

10.5 $K_2B_{12}F_{12}$ into BPh_4^-•2

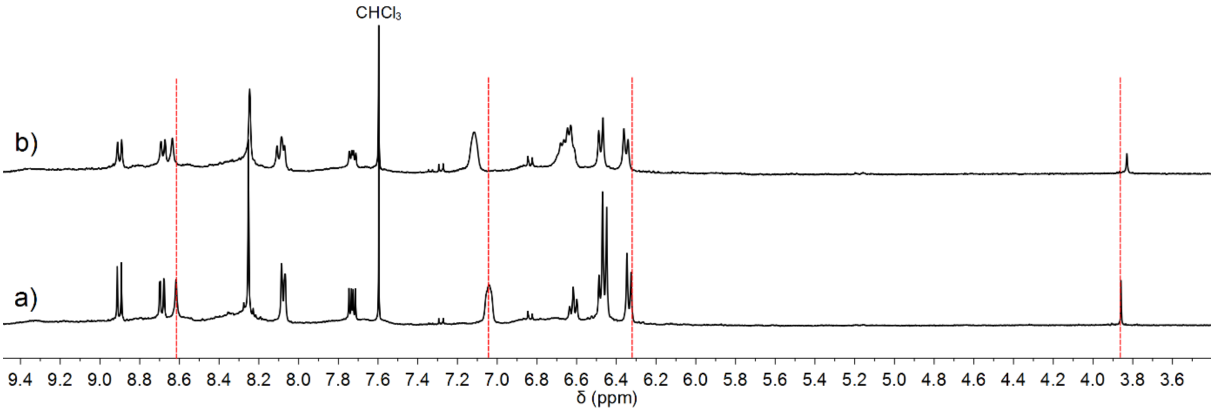

Figure S91. ^{1}H NMR spectra (400 MHz, 298 K, CD_3CN) showing the concurrent binding of BPh_4^- and $B_{12}F_{12}^{2-}$. (a) **2** with one equivalent of ^{n}BuNBPh$_4$; (b) the host-guest complex BPh_4^-•**2** with one equivalent of $K_2B_{12}F_{12}$. Red lines mark the most pronounced resonance shifts, indicating the concurrent encapsulation of BPh_4^- and $B_{12}F_{12}^{2-}$.

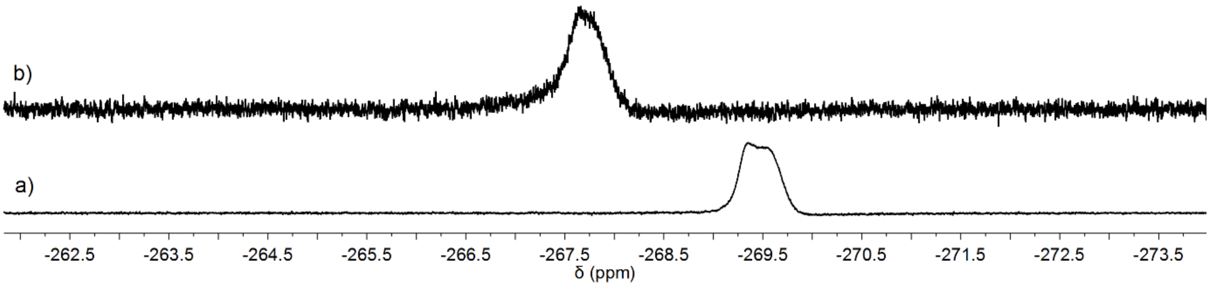

Figure S92. ^{19}F NMR spectra (376 MHz, 298 K, CD_3CN) of (a) free $K_2B_{12}F_{12}$ and (b) **2** with one equivalent of ^{n}BuNBPh$_4$ and one equivalent of $K_2B_{12}F_{12}$.

10.6 ^{n}BuNBPh$_4$ into $B_{12}F_{12}^{2-}$⊂**2**

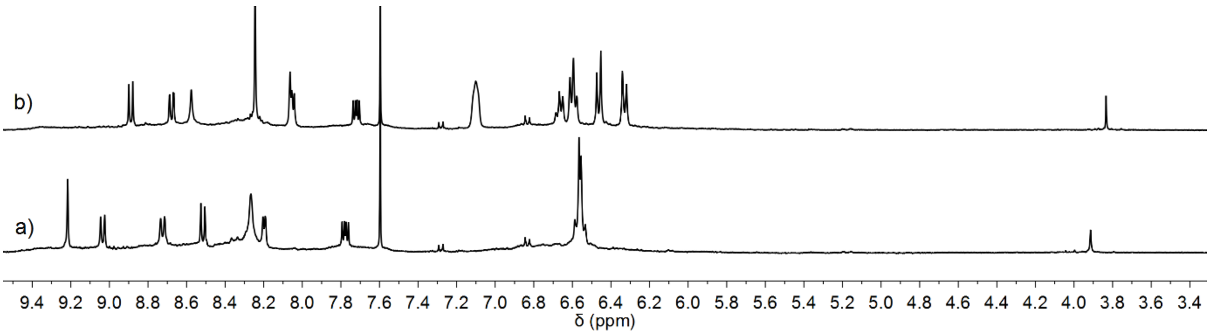

Figure S93. ^{1}H NMR spectra (400 MHz, 298 K, CD_3CN) showing the concurrent binding of BPh_4^- and $B_{12}F_{12}^{2-}$: (a) **2** with one equivalent of $K_2B_{12}F_{12}$; (b) the host-guest complex $B_{12}F_{12}^{2-}$⊂**2** with one equivalent of ^{n}BuNBPh$_4$.

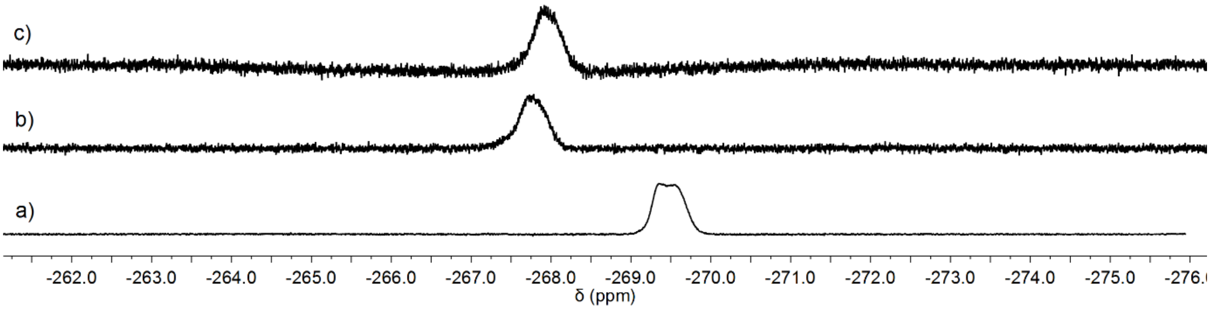

Figure S94. ^{19}F NMR spectra (376 MHz, 298 K, CD_3CN) of (a) free $K_2B_{12}F_{12}$; (b) **2** with one equivalent of $K_2B_{12}F_{12}$ and (c) $B_{12}F_{12}^{2-}$⊂**2** with one equivalent of ^{n}BuNBPh$_4$, showing that $B_{12}F_{12}^{2-}$ is not ejected from the capsule upon the exterior binding of BPh_4^-.

11. Synthesis and characterisation of BPh_4^-•**4**

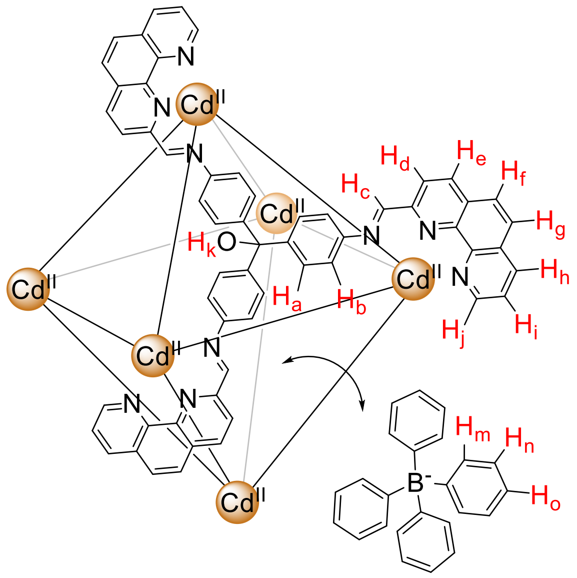

Pararosaniline base **B** (4.90 mg, 1.60 × 10^{-5} mmol, 4 equiv), $Cd(OTf)_2$ (9.86 mg, 2.40 × 10^{-5} mmol, 6 equiv), 2-formylphenanthroline (10.0 mg, 4.80 × 10^{-5} mmol, 12 equiv) and tetrabutylammonium tetraphenylborate (4.50 mg, 8.00 × 10^{-6} mmol, 2 equiv.) were combined in CH_3CN and stirred at 50 °C for 16 hours. The solvent was evaporated and the remaining solid washed alternatively with CH_2Cl_2 (3 × 15 mL) and EtOAc (2 × 15 mL). The residue was dried *in vacuo* to yield a purple crystalline solid (19.6 mg, 3.11 × 10^{-5} mmol, 77%).

Note: (a) While dilute samples were pure by ^{1}H NMR, some portion of subcomponent **B** was always found to persist in solution in more concentrated samples, despite numerous purification cycles. We attribute these signals to dissociation of the cage in solution; however, the cage was stable in its solid form. Thus, 2D NMR and ^{13}C NMR spectra all contain a portion of free **B**. Resonances attributed to the free subcomponents are indicated as such with an asterisk. (b) $^n Bu_4N(OTf)$ was observed to be washed out of the reaction mixture during work-up.

^{1}H NMR (500 MHz, 298 K, CD_3CN): δ 8.74 (d, J = 8.3 Hz, 12H, H_e), 8.73 – 8.70 (m, 12H, H_h), 8.58 (s, 12H, H_c), 8.46 (d, J = 4.7 Hz, 12H, H_j), 8.24 (d, J = 9.1 Hz, 12H, H_g), 8.20 (d, J = 9.1 Hz, 12H, H_f), 7.88 (d, J = 8.3 Hz, 12H, H_d), 7.82 (dd, J = 8.3, 4.7 Hz, 12H, H_i), 7.09 (b, 16H, H_m), 6.62 (m, 48H, H_a & H_b), 6.60 (b, 8H, H_o), 6.59 – 6.47 (m, 16H, H_n), 3.85 (s, 4H, H_k) ppm.

LR-ESI-MS [charge, calculated for $C_{232}H_{148}N_{36}O_4Cd_6(CF_3SO_3)_{10}(BC_{24}H_{20})_2$]: m/z = 1427.9 [**4**$(OTf)_6(BPh_4)_2^{4+}$, 1427.8], 1112.2 [**4**$(OTf)_5(BPh_4)_2^{5+}$, 1112.4], 1080.1 [**4**$(OTf)_6(BPh_4)^{5+}$, 1080.2], 901.8 [**4**$(OTf)_4(BPh_4)_2^{6+}$, 902.2], 873.8 [**4**$(OTf)_5(BPh_4)^{6+}$, 874.0], 751.9 [**4**$(OTf)_3(BPh_4)_2^{7+}$, 752.0], 727.5 [**4**$(OTf)_4(BPh_4)^{7+}$, 727.5], 639.3 [**4**$(OTf)_2(BPh_4)_2^{8+}$, 639.4], 617.8 [**4**$(OTf)_3(BPh_4)^{8+}$, 617.9].

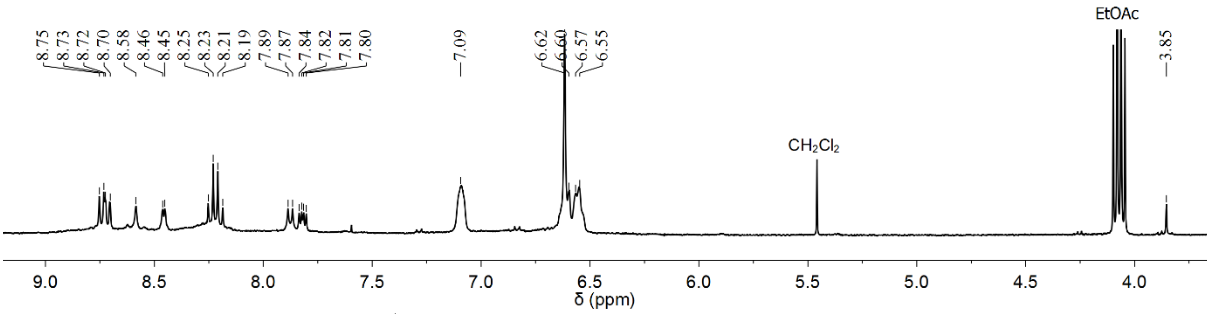

Figure S95. Aromatic region of the ^{1}H NMR spectrum (500 MHz, 298 K, CD_3CN) of BPh_4^-•**4**.

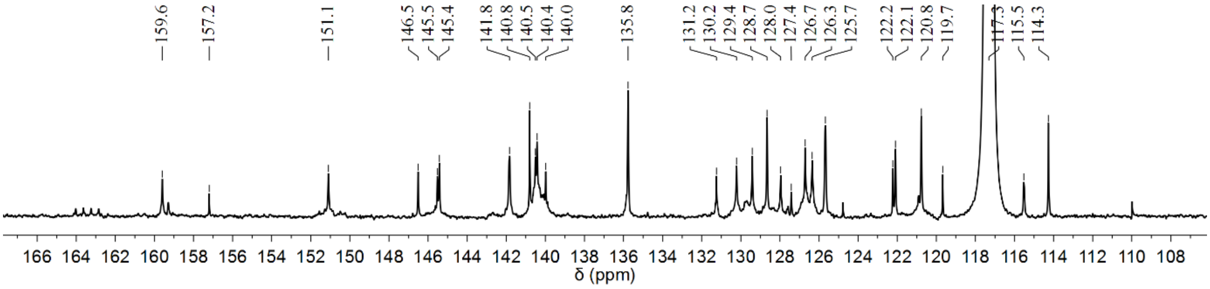

Figure S96. Aromatic region of the ^{13}C NMR spectrum (125 MHz, 298 K, CD_3CN) of BPh_4^-•**4**.

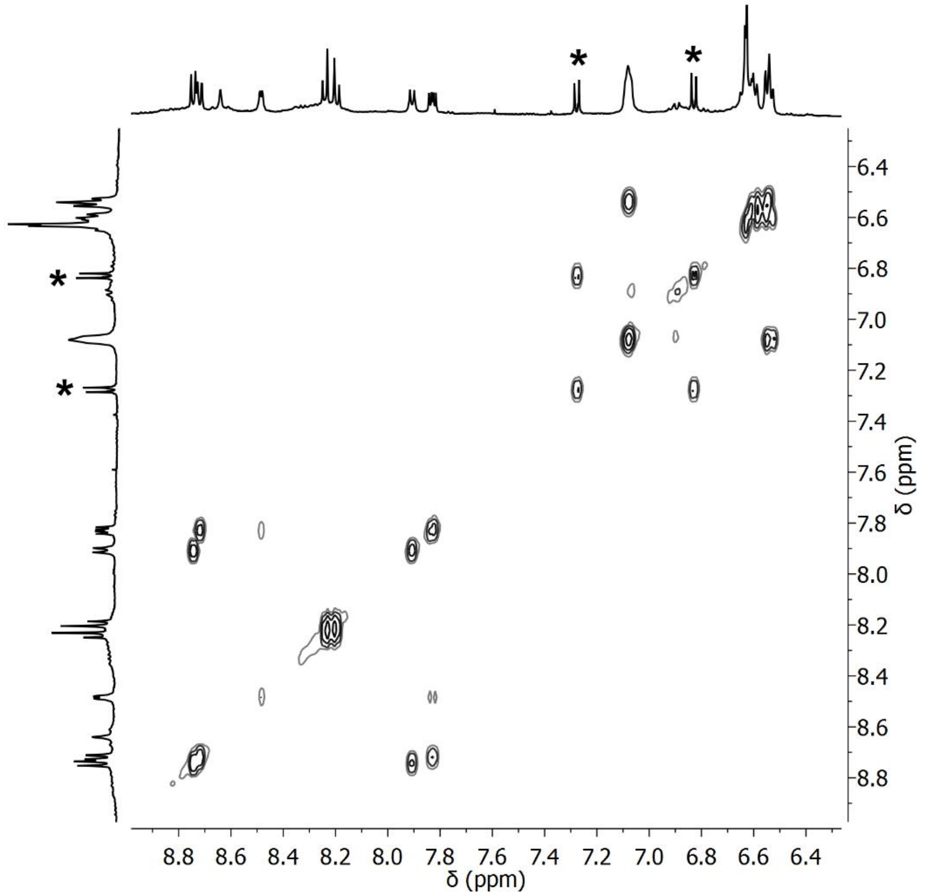

Figure S97. Aromatic region of the ^{1}H-^{1}H COSY spectrum (500 MHz, 298 K, CD_3CN) of BPh_4^-•**4**.

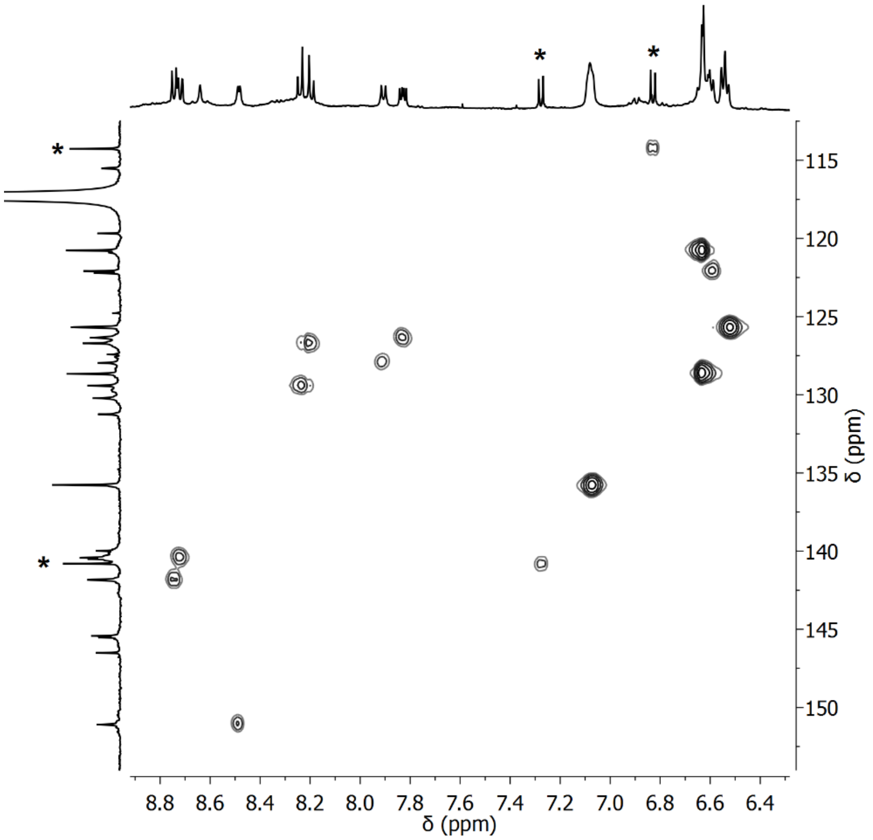

Figure S98. Aromatic region of the ^{1}H-^{1}H HSQC spectrum (500 MHz, 298 K, CD_3CN) of BPh_4^-•**4**.

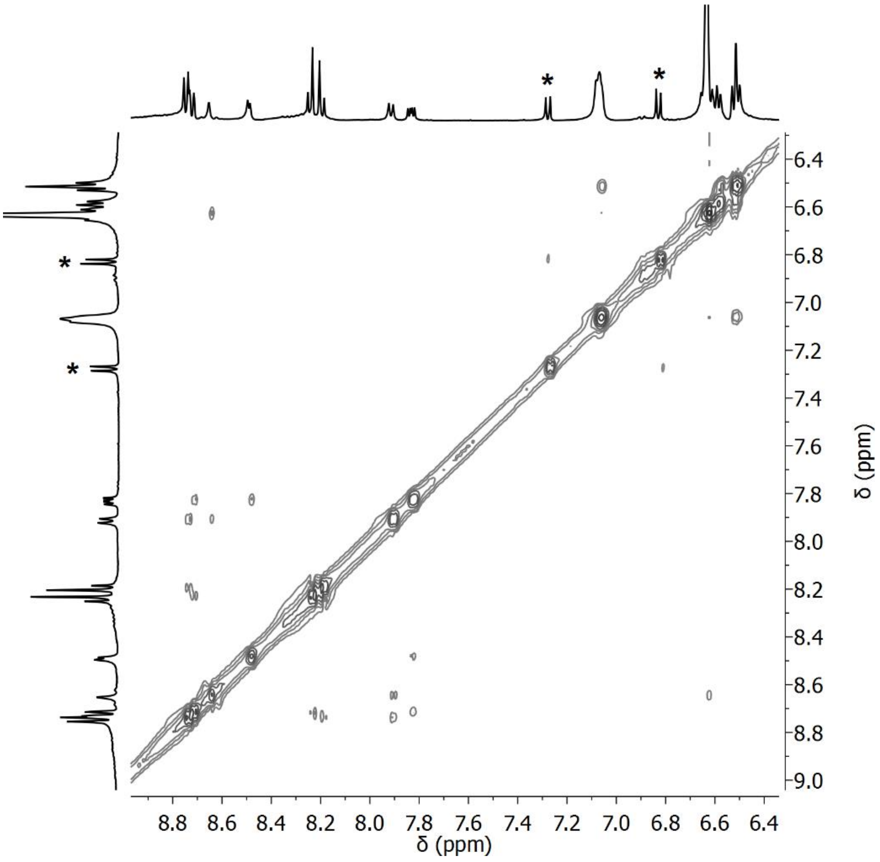

Figure S99. Aromatic region of the ^{1}H-^{1}H NOESY spectrum (500 MHz, 298 K, CD_3CN) of BPh_4^-•**4**.

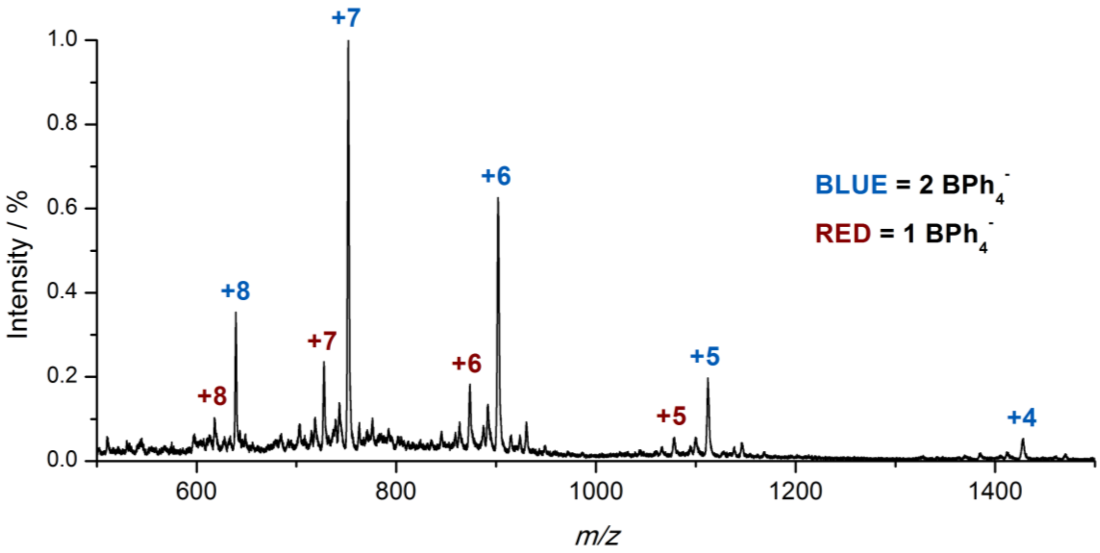

Figure S100. Low resolution ESI mass spectrum of BPh_4^-•**4**.

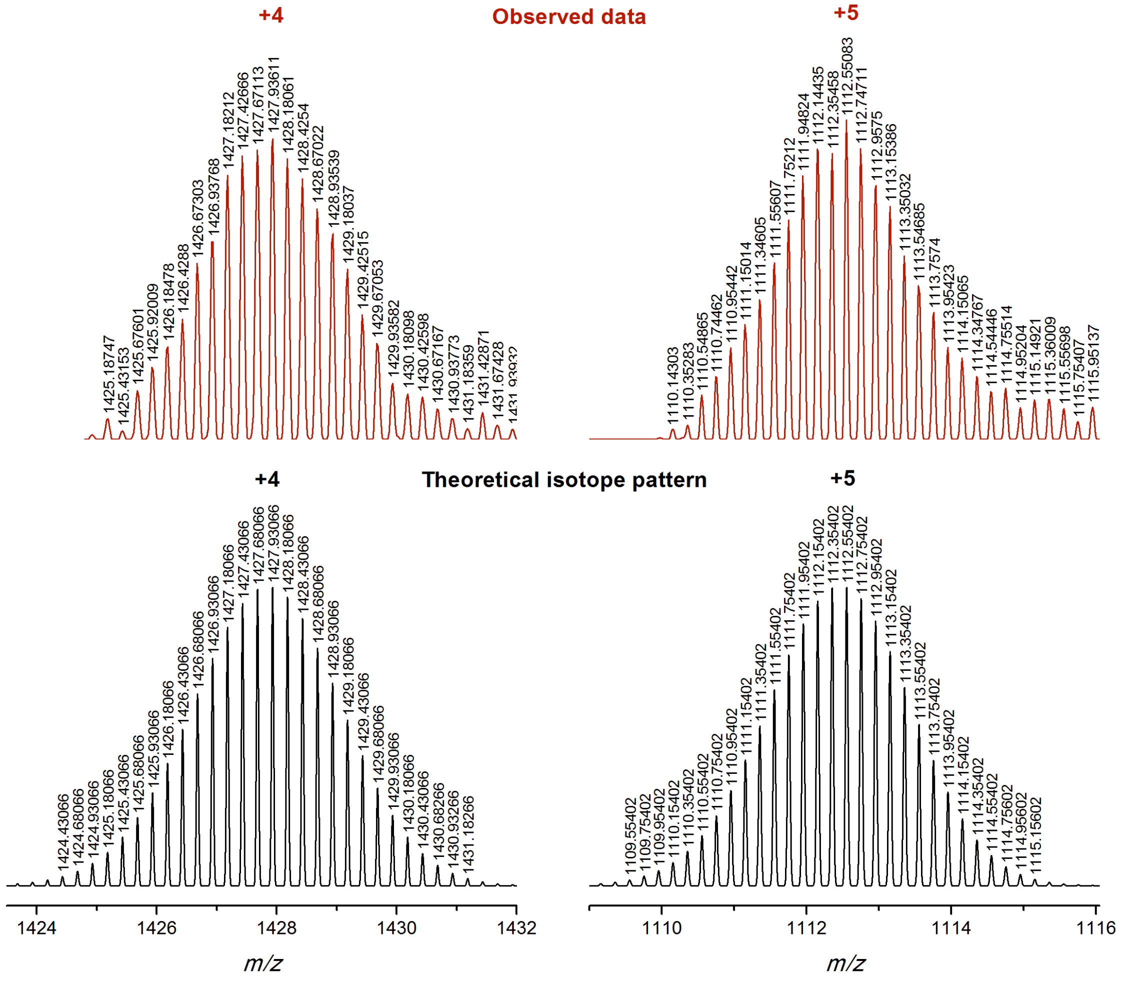

Figure S101. High resolution ESI mass spectra of **3**, showing the observed z = +4 (corresponding to **4**$(OTf)_6(BPh_4)_2^{4+}$) and +5 (corresponding to **4**$(OTf)_5(BPh_4)_2^{5+}$) charges (top), compared to the theoretical isotope patterns for each (bottom).

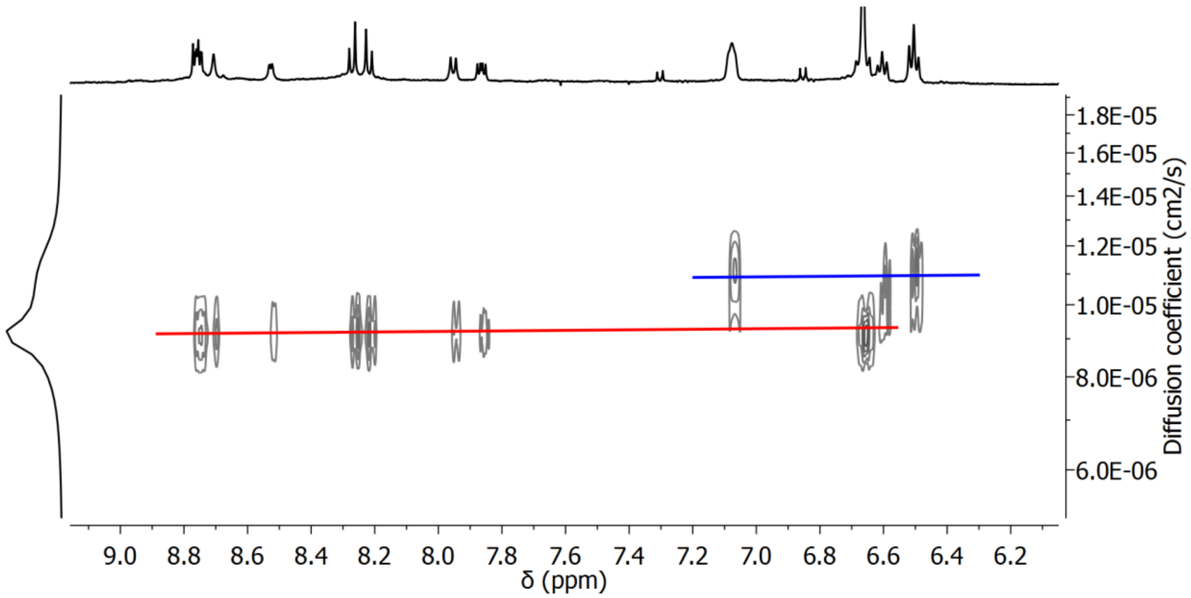

Figure S102. ^{1}H DOSY spectrum (500 MHz, 298 K, CD_3CN) of BPh_4^-•**4**. The cage **4** displayed a diffusion coefficient of 9.2 × 10^{-6} cm^2 s^{-1} (red line). The occluded BPh_4^- displayed a diffusion coefficient of 1.1 × 10^{-5} (blue line). This is slower than the literature value for free BPh_4^- (1.7 × 10^{-5} cm^2 s^{-1})[13], indicating binding to **4**.

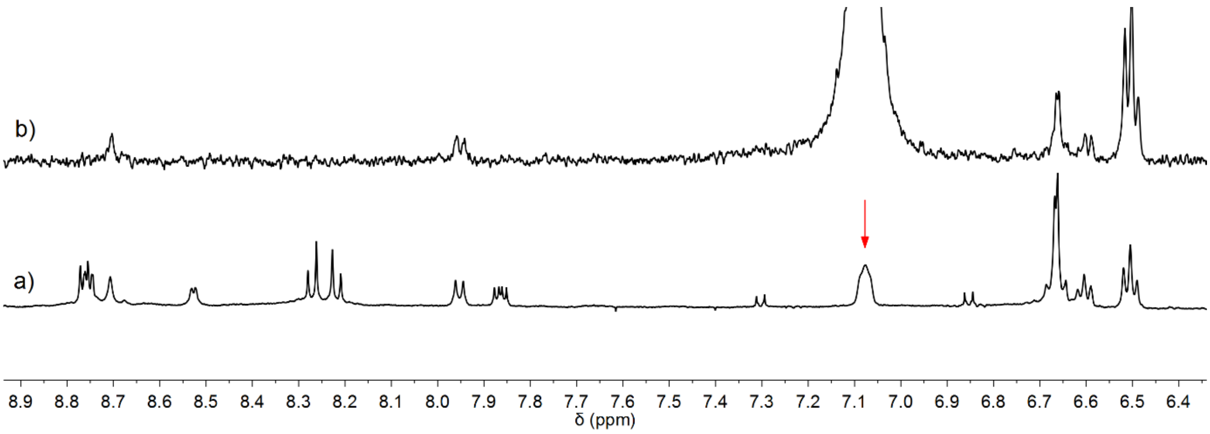

Figure S103. (a) ^{1}H NMR spectrum (500 MHz, 298 K, CD_3CN) of BPh_4^-•**4**. (b) 1D selective ^{1}H NOESY spectrum (500 MHz, 298 K, CD_3CN) of BPh_4^-•**4**, irradiating the *ortho* proton of the BPh_4^- guest. From left to right, NOE peaks to the imine, proximal phenanthroline and phenyl protons were observed.

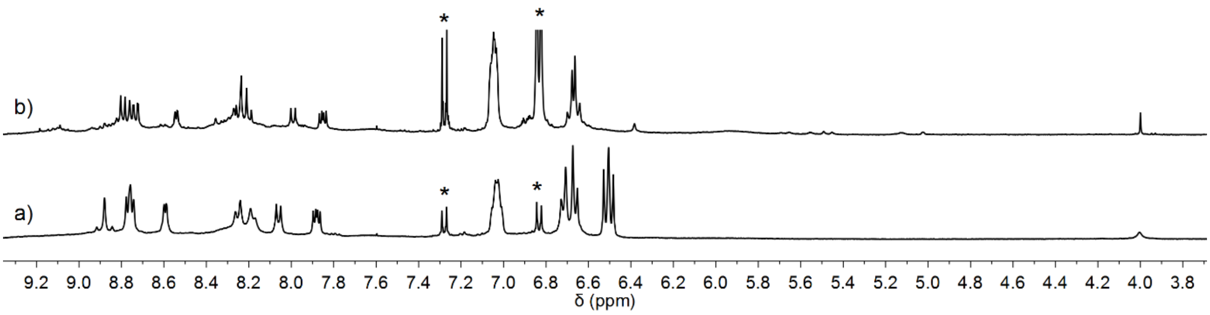

Figure S104. Templation of **4** with *para*-substituted tetraphenylborates (a) $B(C_6H_4F)_4^-$•**4** and (b) $B(C_6H_4Cl)_4^-$•**4**. Resonances corresponding to the free subcomponent **B** are marked with an asterisk.

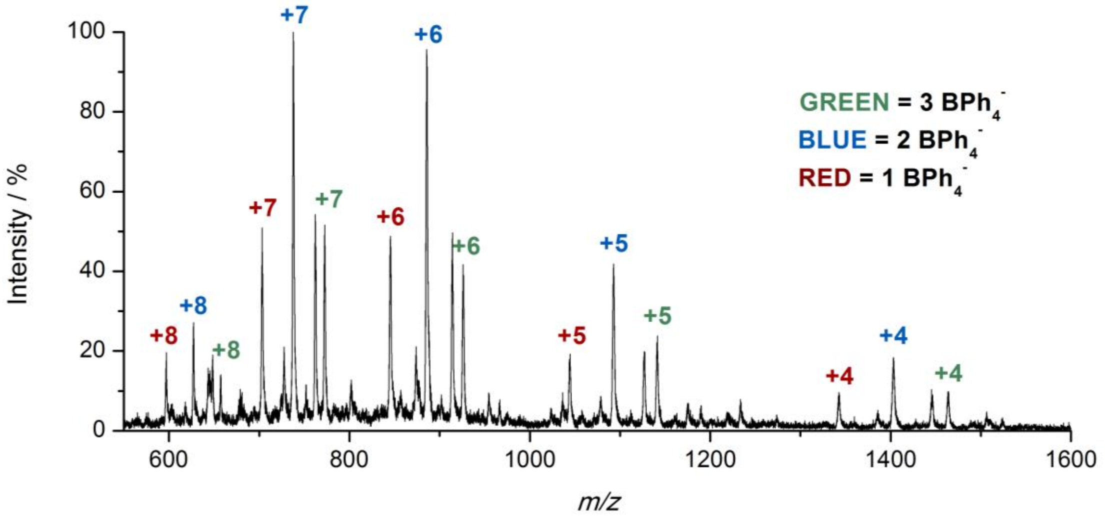

Figure S105. Low resolution ESI mass spectrum of $B(C_6H_4F)_4^{-}$•**4**.

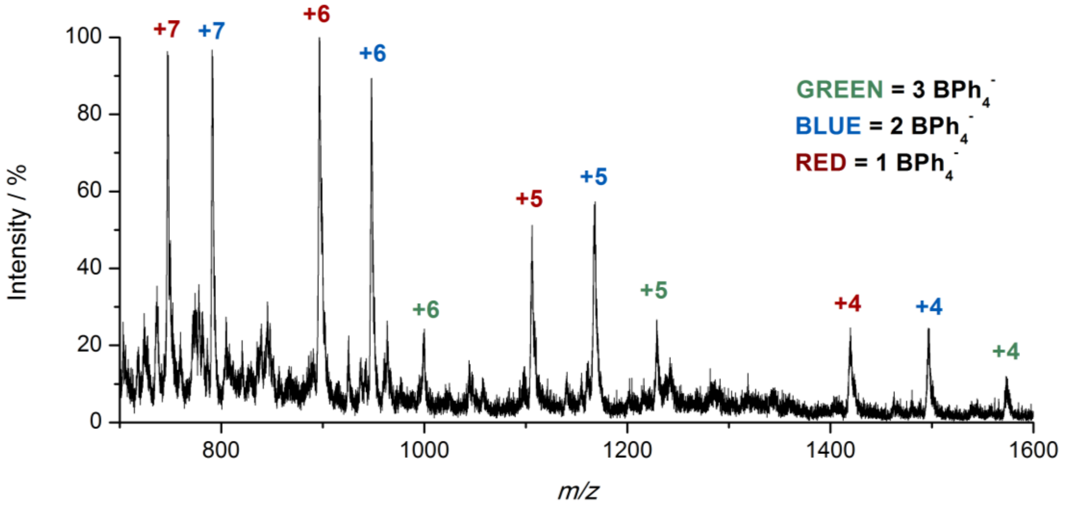

Figure S106. Low resolution ESI mass spectrum of $B(C_6H_4Cl)_4^{-}$•**4**.

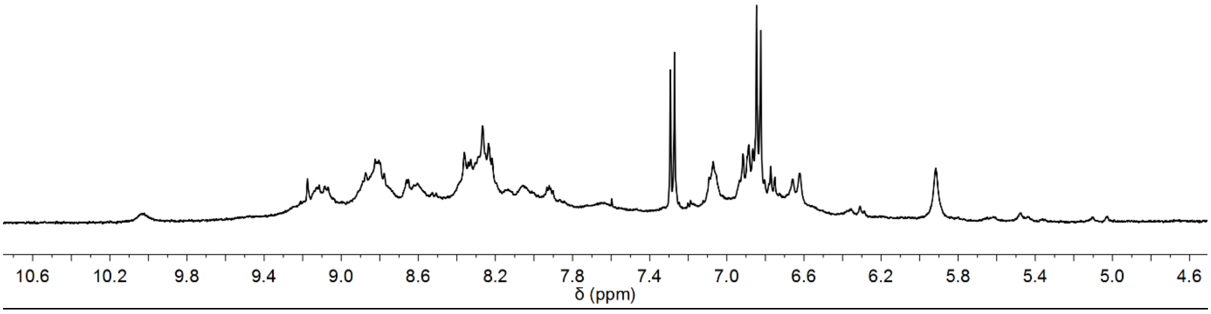

Figure S107. ^{1}H NMR spectrum (400 MHz, 298 K, CD_3CN) of a mixture of **B**, $Cd(OTf)_2$ and 2-formylphenanthroline heated to 50°C for 16 hours, showing no clean formation of **4**.

12. Host-guest chemistry of BPh_4^-•**4**

12.1 $CsCB_{11}H_{12}$

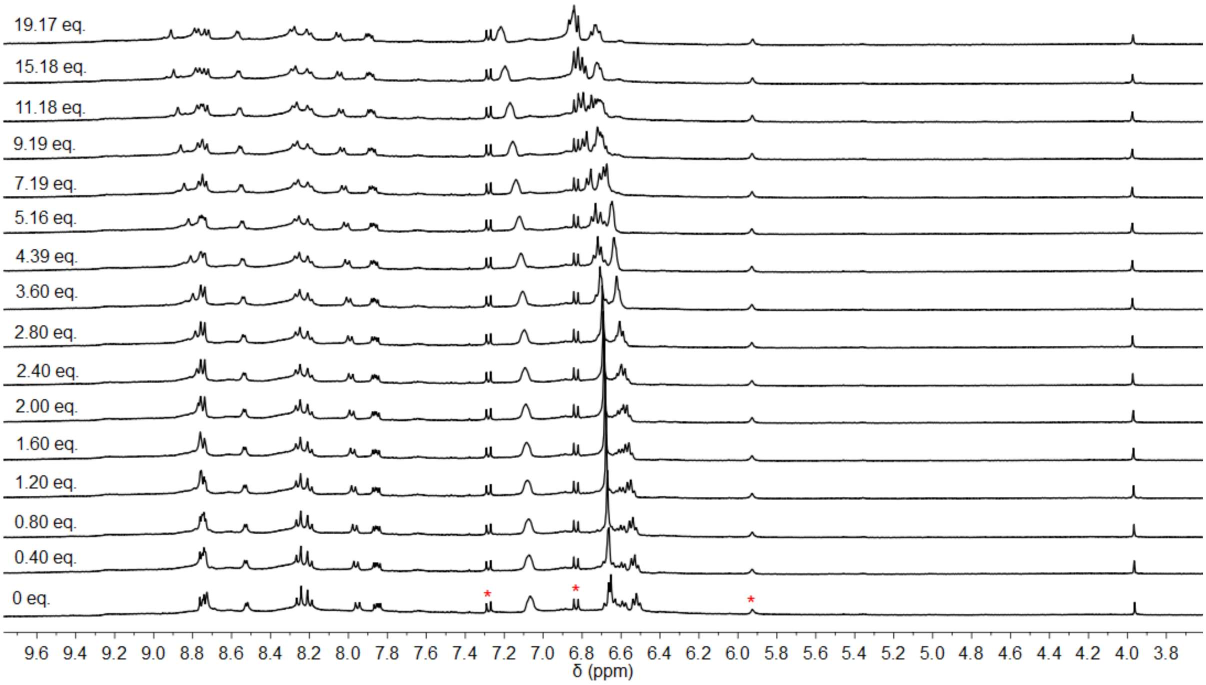

Figure S108. ^{1}H NMR titration (500 MHz, 298 K) of $CsCB_{11}H_{12}$ into a solution of BPh_4^-•**4** in CD_3CN (equivalents of anion are labeled on individual spectra, red asterisks marks a small portion of **B**).

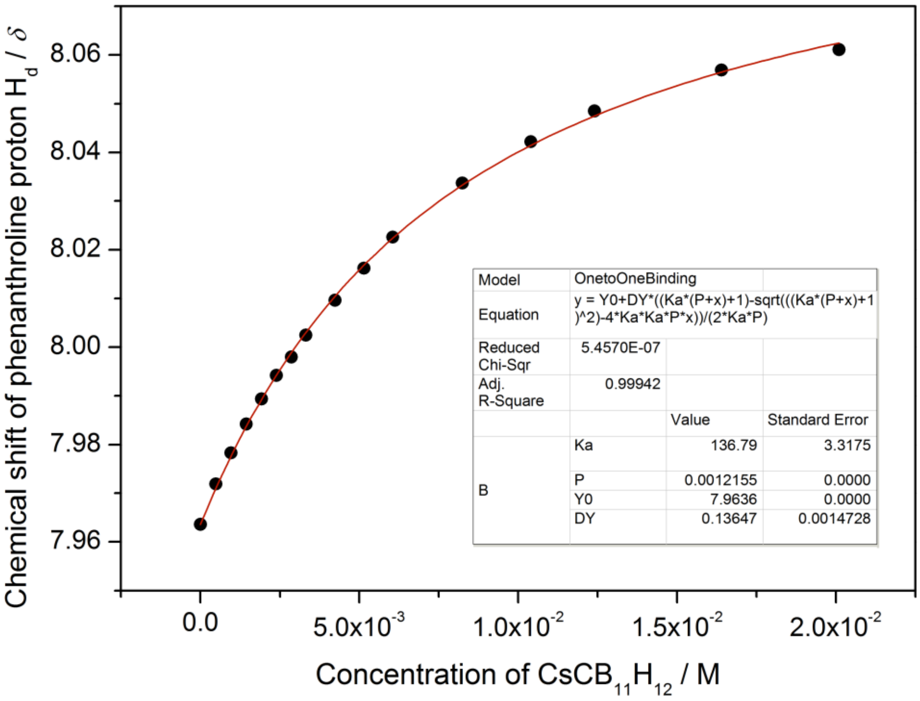

Figure S109. Binding isotherm (1:1 system) fit to the chemical shift of the imine proton *vs.* the concentration of $^nBu_4NBPh_4$ added to determine binding affinity (K_a).

13. References

[1] M. Zhang, R. Gao, X. Hao, W.-H. Sun, *J. Organomet. Chem.* **2008**, *693*, 3867-3877.

[2] W. Meng, T. K. Ronson, J. K. Clegg, J. R. Nitschke, *Angew. Chem. Int. Ed.* **2013**, *52*, 1017-1021.

[3] D. Maity, T. Govindaraju, *Chem. Commun.* **2010**, *46*, 4499-4501.

[4] Bruker, *SAINT*, Bruker ASX Inc., Madison, Wisconsin, USA, **2012**.

[5] Agilent, *Crysalis PRO*, Agilent Technologies, Ltd., Yarnton, Oxfordshire, England, **2014**.

[6] Bruker, *SADABS*, Bruker AXS, Inc., Madison, Wisconsin, USA, **2001**.

[7] L. Farrugia, *J. Appl. Crystallogr.* **2012**, *45*, 849-854.

[8] G. M. Sheldrick, *Acta Crystallogr. Sect. A: Found. Crystallogr.* **2015**, *71*, 3-8.

[9] A. L. Spek, *Acta Crystallogr. Sect. C: Cryst. Struct. Commun.* **2015**, *71*, 9-18.

[10] A. L. Spek, *Acta Crystallogr. Sect. D. Biol. Crystallogr.* **2009**, *65*, 148-155.

[11] G. J. Kleywegt, T. A. Jones, *Acta Crystallogr. Sect. D. Biol. Crystallogr.* **1994**, *50*, 178-185.

[12] Y. R. Hristova, M. M. J. Smulders, J. K. Clegg, B. Breiner, J. R. Nitschke, *Chem. Sci.* **2011**, *2*, 638-641.

[13] W. J. Ramsay, J. R. Nitschke, *J. Am. Chem. Soc.* **2014**, *136*, 7038-7043.

[14] Thordarson, K., Bindfit, http://app.supramolecular.org/bindfit/, accessed 23rd March 2016.

[15] F. Ulatowski, K. Dąbrowa, T. Bałakier, J. Jurczak, *J. Org. Chem.* **2016**, *81*, 1746-1756.
